# Cu(I)-Catalyzed Alkynylation of Quinolones

**DOI:** 10.1021/acs.orglett.2c00020

**Published:** 2022-01-31

**Authors:** Aitor Maestro, Sebastien Lemaire, Syuzanna R. Harutyunyan

**Affiliations:** † Stratingh Institute for Chemistry, University of Groningen, 9747 AG Groningen,The Netherlands; ‡ Janssen Pharmaceutica, Chemical Process Research & Development, Turnhoutseweg 30, B-2340 Beerse, Belgium

## Abstract

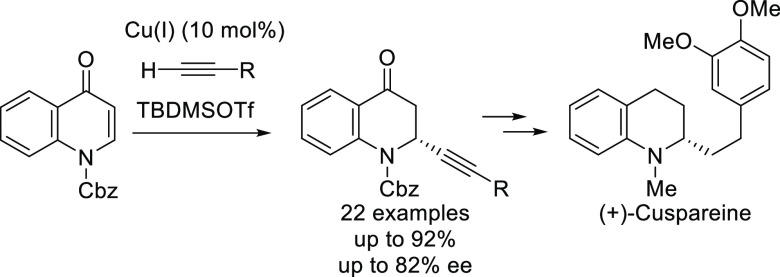

Herein we report the first alkynylation of quinolones with terminal alkynes under mild reaction conditions. The reaction is catalyzed by Cu(I) salts in the presence of a Lewis acid, which is essential for the reactivity of the system. The enantioselective version of this transformation has also been explored, and the methodology has been applied in the synthesis of the enantioenriched tetrahydroquinoline alkaloid cuspareine.

Quinolone (**A**) derivatives such as ciprofloxacin **B** are well known as broad-spectrum bacteriocidal agents^1−6^ (Figure 1), which consequently has prompted the development of several methods for their synthesis.^7−9^ However, molecules with abundant sp^3^ carbons in their structure, such as dihydroquinolone derivatives, are becoming increasingly attractive for the development of potential drug candidates.^10^ In this context, dihydroquinolone derivative **C** has been reported as a 5-HT6 serotonin receptor,^11^ and other dihydroquinolones have been shown to be applicable as crucial intermediates in the production of martinellic acid **D**(12−14) and (+)-angustureine **E**.^15−17^ Therefore, the development of new efficient methodologies for the synthesis of dihydroquinolones would improve the chemical toolbox for the synthesis of biologically relevant molecules.

**Figure 1:**
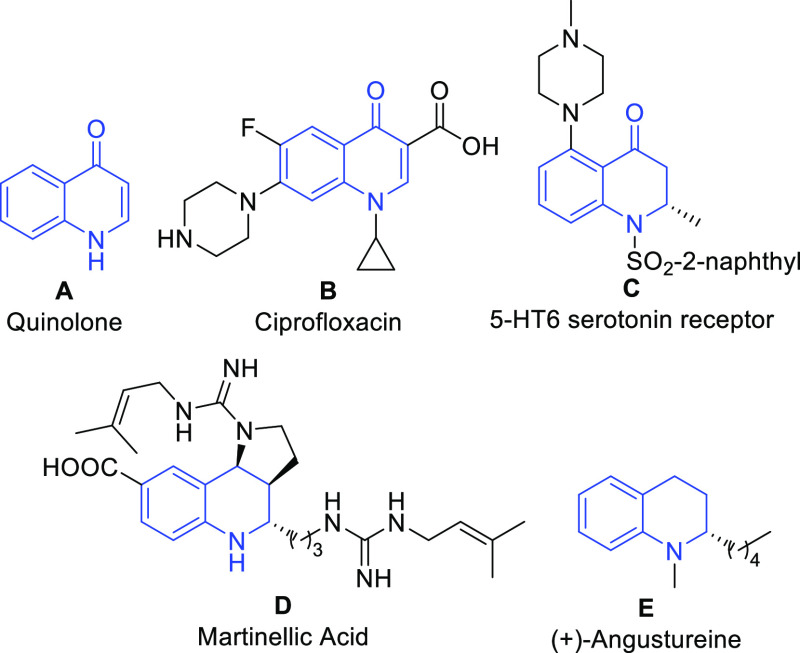
Relevant quinolones and their derivatives.

During the last couple of decades, several examples of dihydroquinoline synthesis based on the functionalization of quinolones have been reported. These quinolone functionalizations, including Pd- and Rh-catalyzed arylations (Scheme 1a)^18−20^ and, more recently, Cu(I)-catalyzed alkylations using organomagnesium and organoaluminum reagents (Scheme 1b),^15,21^ afford 4-oxo-2,3-dihydroquinolines. Despite this progress with arylations and alkylations, alkynylations of quinolones have not been reported. We were interested in exploring alkynylation reactions of quinolones to extend the structural variety of functionalized dihydroquinolones. The synthesis of two alkynylated 4-oxo-2,3-dihydroquinolines has been previously described,^22,23^ making use of 4-alkoxyquinolines and alkynylmagnesium bromides or organozinc chlorides as nucleophiles in a lengthy multistep procedure. The limited scope of readily available alkynylmagnesium bromides and the lengthy multistep procedure limit the potential of this method.

**Scheme 1:**
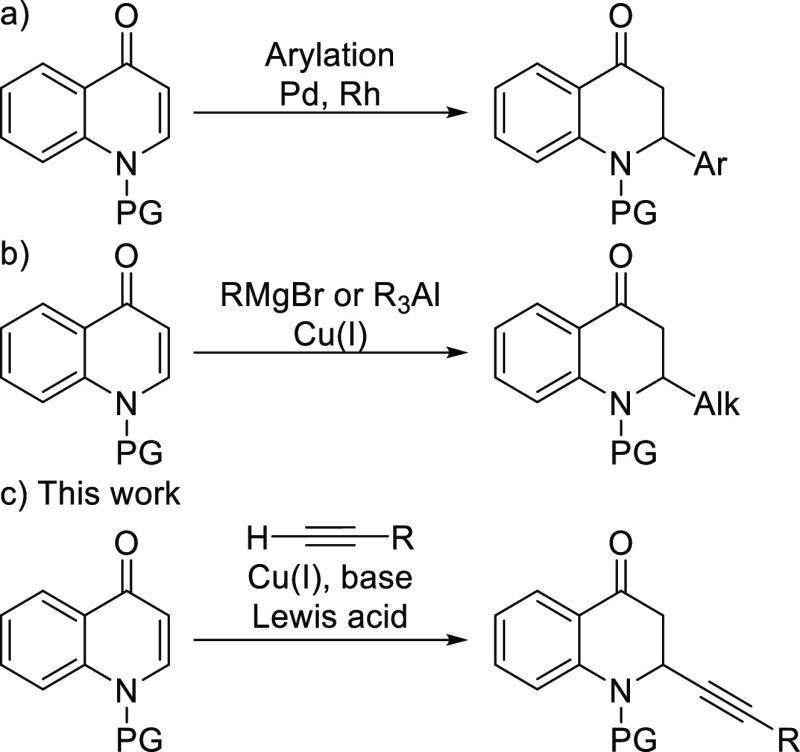
Functionalization of 4-Quinolones: (a) Arylation, (b) Alkylation, and (c) Alkynylation (This Work)

On the contrary, the use of readily available and structurally diverse terminal alkynes as pronucleophiles, along with the mild reaction conditions, offers an attractive strategy for the synthesis of structurally diverse quinolone derivatives. Several examples of this approach, including Cu(I)-catalyzed alkynylations of (thio)chromones^24−27^ and quinolines^13,28^ and allylic alkylations of terminal alkynes,^29^ have been published during the last several years, but the direct Cu(I)-catalyzed alkynylation of quinolones has not been accomplished so far.^30,31^

Herein we report the first example of the direct Cu(I)-catalyzed alkynylation of 4-quinolones with terminal alkynes as pronucleophiles (Scheme 1c). This methodology offers a new path for functionalizing quinolones with an alkynyl moiety that complements the existing synthetic routes toward 4-oxo-2,3-dihydroquinolines.

At the start of this work, the optimization studies were carried out for the alkynylation reaction between Cbz-protected quinolone **1a** and phenylacetylene **2a** in the presence of base DIPEA and catalytic amounts of Cu(I) salt. On the basis of our group’s experience with Lewis-acid-promoted Cu(I)-catalyzed conjugate additions,^15,32−35^ we evaluated the effect of several Lewis acids to enhance the electrophilicity of the quinolone substrate **1a**. Excellent conversion to the desired addition product **3a** was observed in the presence of a stoichiometric amount of *tert*-butyldimethylsilyl triflate (TBDMSOTf) after stirring overnight (Table 1, entry 1). Shortening the reaction time to 4 h had little effect on the substrate conversion (entry 2). Further optimization of the solvent and the base (see the Supporting Information) confirmed the conditions in entry 1 as the most optimal. Next, we evaluated the effect of the protecting group of the quinolone substrate on the reaction outcome.

**Table 1:**
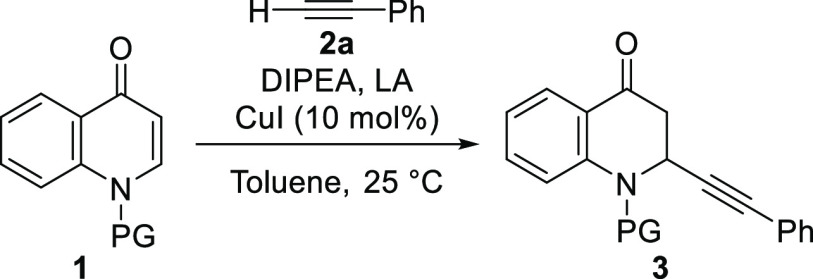
Optimization of Cu(I)-Catalyzed Alkynylation^a^

entry	Lewis acid	protecting group	*t* (h)	conv. (%)^b^
1	TBDMSOTf	Cbz (**1a**)	18	>99
2	TBDMSOTf	Cbz (**1a**)	4	96
3	TBDMSOTf	H (**1b**)	18	0
4	TBDMSOTf	Bn (**1c**)	18	0
5	TBDMSOTf	Boc (**1d**)	18	<10
6^c^	TBDMSOTf	Cbz (**1a**)	18	0
7^d^	TBDMSOTf	Cbz (**1a**)	18	20
8		Cbz (**1a**)	18	0
9	TMSBr	Cbz (**1a**)	18	<10
10	TMSI	Cbz (**1a**)	18	<10
11	TMSOTf	Cbz (**1a**)	18	30
12	TESOTf	Cbz (**1a**)	18	63
13	BF_3_·Et_2_O	Cbz (**1a**)	18	0

a Reaction conditions: quinolone **1** (0.1 mmol), CuI (10 mol %), toluene (1 mL), alkyne **2a** (1.3 equiv), DIPEA (1.6 equiv), LA (1.2 equiv).

b Conversion was determined by ^1^H NMR with respect to the quinolone.

c No CuI was used.

d 20 mol % of LA was used.

No conversion was observed when unprotected or benzyl-protected quinolones were used (entries 3 and 4). Moreover, replacing the Cbz protecting group on the quinolone substrate by a Boc group resulted in a significant drop in the conversion (entry 5). Further studies confirmed that the presence of a copper salt and a stoichiometric amount of a Lewis acid are mandatory to promote the reaction to completion. No conversion of quinolone was observed in the absence of copper salt or using only a catalytic amount of a Lewis acid (entries 6–8). With silyl-based Lewis acids other than TBDMSOTf, a lower substrate conversion was obtained (entries 9–13). Only traces of the addition product **3a** were obtained when trimethylsilyl halides were used instead (entries 9 and 10). The use of stronger silicon-based Lewis acids such as trimethylsilyl (TMS) and triethylsilyl (TES) triflates resulted in moderate reaction rates (entries 11 and 12), whereas the boron-based Lewis acid BF_3_·Et_2_O did not improve the reaction outcome either (entry 13). The superiority of TBDMSOTf over other explored silyl triflates can be rationalized by the higher stability of a possible TBDMS-enolate intermediate formed during the reaction.

Having the optimized conditions in hand (entry 1), we moved to study the scope of the reaction. For this purpose, various alkynes and quinolones were tested (Scheme 2).

**Scheme 2:**
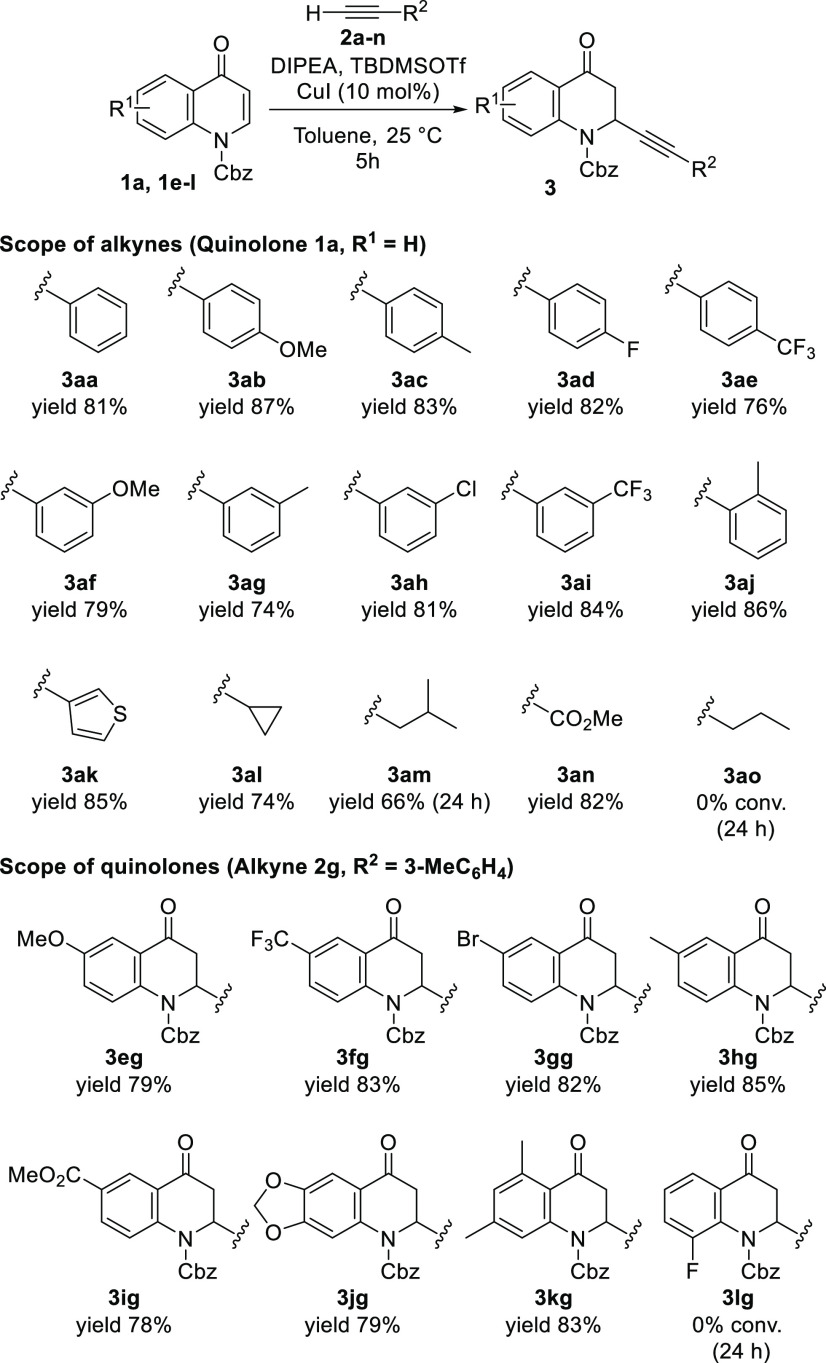
Scope of the Reaction Reaction conditions: quinolone **1** (0.1 mmol), CuI (10 mol %), toluene (1 mL), alkyne **2** (1.3 equiv), DIPEA (1.6 equiv), TBDMSOTf (1.2 equiv).

The reaction was successfully extended to several aromatic terminal alkynes bearing electron-donating and electron-withdrawing groups and four-, three-, and two-substituted aromatic rings (**3ab**–**3aj**). An excellent yield was also obtained with heteroaromatic alkyne **3ak**. Similar results were obtained when using cyclopropyl-, isobutyl-, and ester-substituted alkynes (**3al**–**3an**). Surprisingly, the linear terminal alkyne 1-pentyne was unreactive under the optimized reaction conditions (**3ao**). The limited reactivity of alkyl alkynes and the lack of reactivity of linear alkynes are consistent with the literature observations in other Cu-catalyzed reactions.^31,36,37^ Various quinolones can be used with this catalytic system. Excellent yields were obtained for quinolones both with activating and with deactivating groups present in the quinolone ring (**3eg**–**3ig**) and for those with disubstituted substrates (**3jg** and **3kg**).

Next, we envisaged that the use of a copper salt in combination with a chiral ligand could lead to enantioinduction through the binding of the chiral copper complex to the quinolone. After some optimization, we found that the copper complex of chiral diphenylphosphine ligand BPE catalyzes the alkynylation of several quinolone substrates with enantioselectivities in the range of 50–82% ee (Scheme 3), thus confirming the feasibility of the catalytic asymmetric synthesis of these molecules.

**Scheme 3:**
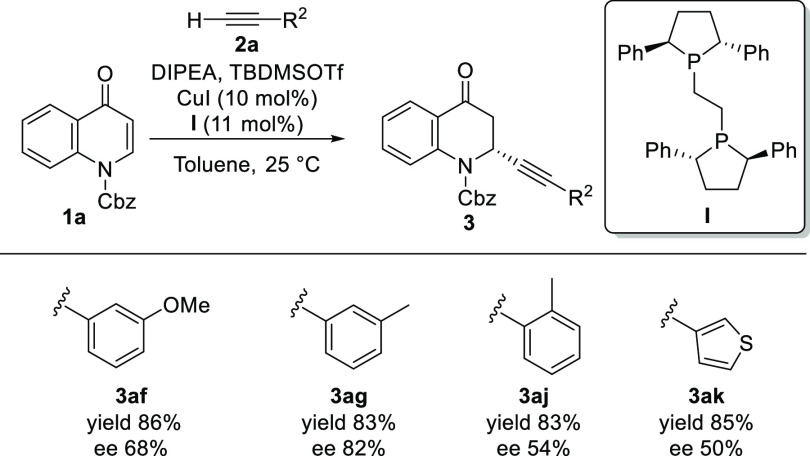
Enantioselective Alkynylation of Quinolones Reaction conditions: quinolone **1a** (0.1 mmol), CuI (10 mol %), **I** (11 mol %), toluene (1 mL), alkyne **2** (1.3 equiv), DIPEA (1.6 equiv), TBDMSOTf (1.2 equiv). ee values were determined by chiral high-performance liquid chromatography (HPLC) or supercritical fluid chromatography (SFC).

The robustness of the methodology was tested by scaling up the synthesis of **3aa** to 1 mmol (Scheme 4). Moreover, the selective deprotection of the Cbz group was successfully performed under basic conditions to afford dihydroquinolin-4-one **4** in 83% yield.

**Scheme 4:**
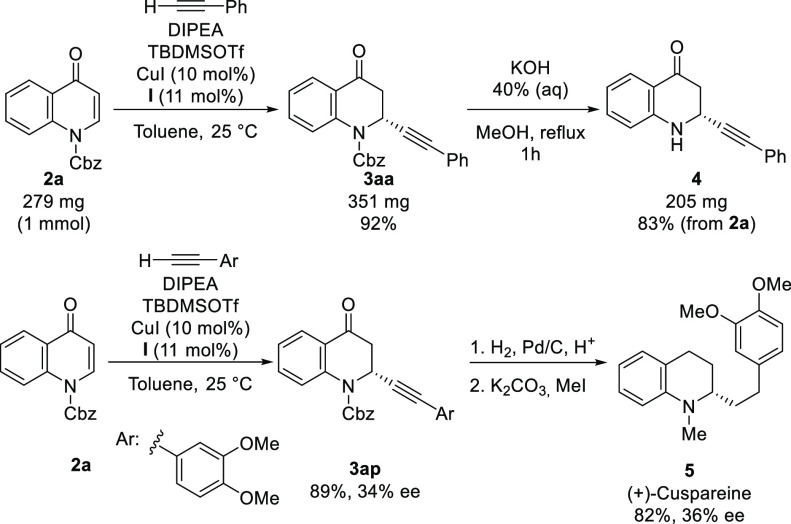
Scaling Up and Synthetic Applications of Quinolone Derivatives **3**

The hydrogenation of **3ap** with Pd on activated carbon under acidic conditions followed by the methylation of the nitrogen atom afforded the Hancock alkaloid (+)-cuspareine (**5**) without racemization, allowing the determination of the absolute configuration of the stereogenic carbon by comparing the optical rotation of cuspareine with literature data.^16,17^

In summary, an efficient methodology for the alkynylation of quinolones with readily available terminal alkynes has been accomplished. This methodology tolerates the presence of several functional groups in both the quinolone and alkyne reagents and complements the previously developed arylation and alkylation reactions of quinolones. We have also demonstrated the feasibility of an enantioselective version and applied the current methodology to the synthesis of the enantioenriched Hancock alkaloid (+)-cuspareine. Further studies are under way, aiming to improve the enantioselective variant and shed light on the underlying mechanism.

## Supporting Information Available

Full experimental procedures, characterization data, NMR spectra, and chiral HPLC (PDF)
