# Supplementary material for: Cu(I)-Catalyzed Alkynylation of Quinolones
Source: Org Lett. 2022 Jan 31;24(5):1228–31. doi: 10.1021/acs.orglett.2c00020 (PMC8845045; doi:10.1021/acs.orglett.2c00020)
Supplement: Supplementary file 1 — ol2c00020_si_001.pdf [file ol2c00020_si_001.pdf]

Cu(I)-catalyzed alkynylation of quinolones

Aitor Maestro[1], Sebastien Lemaire*[2], Syuzanna R. Harutyunyan*[1]

[1] Stratingh Institute forChemistry, University of Groningen, 9747 AG Groningen,The Netherlands.

[2] Janssen Pharmaceutica, Chemical Process Research & Development, Turnhoutseweg 30, B-2340 Beerse, Belgium.

General experimental information S-2

Synthesis and characterization of quinolones **1**.. S-3

Synthesis and characterization of 4-oxo-2-alkynyl-dhydroquinolines **3**. S-7

Synthesis and characterization of compounds **4** and **5**.. S-26

NMR spectra of quinolones **1**.. S-29

NMR spectra of 4-oxo-2-alkynyl-dhydroquinolines **3** S-42

NMR spectra of compounds **4** and **5**.. S-71

General experimental information

All reactions were carried out with anhydrous solvents (vide infra) under a nitrogen atmosphere using oven-dried glassware and standard Schlenk techniques. Reactions were monitored by ^{1}H NMR. Purification of the products was performed by column chromatography using Merck 60 Å 230-400 mesh silica gel. Components were visualized by UV and $KMnO_4$ staining (TLC). NMR data were collected on Varian MercuryPlus, Varian400MR (^{1}H at 400 MHz; ^{13}C at 101 MHz) or Bruker NEO (^{1}H at 600 MHz; ^{13}C at 151 MHz) equipped with a 5 mm z-gradient broadband probe. Chemical shifts are reported in parts per million (ppm) relative to the residual solvent peak ($CDCl_3$, ^{1}H: 7.26 ppm; ^{13}C: 77.16 ppm). Coupling constants are reported in Hertz. Multiplicity is reported with the usual abbreviations. Enantiomeric excesses (*ee*) were determined by chiral HPLC analysis using a Shimadzu LC-10ADVP HPLC equipped with a Shimadzu SPD-M10AVP diode array detector, or by chiral SFC analysis using a Waters UPC2 system. Exact mass spectra were recorded on a LTQ Orbitrap XL apparatus with ESI ionization.

Unless otherwise indicated, reagents and substrates were purchased from commercial sources and used as received. Solvents not required to be dry were purchased as technical grade and used as received. Dry solvents were freshly collected from a dry solvent purification system before use. Inert atmosphere experiments were performed with standard Schlenk techniques with dried (P_2O_5) nitrogen gas. All reported compounds were characterized by ^{1}H and ^{13}C NMR, HRMS and compared with literature data. All new compounds were fully characterized by ^{1}H and ^{13}C NMR and HRMS techniques.

Angles of rotation of enantioenriched compounds were measured with a SCHMIDT+HAENSCH Polartronic MH8. The specific rotation was calculated with the following equation, where T is the temperature in °C, D is the sodium D-line emission, α is the angle of rotation, c is the concentration of the solution in g per 100 mL and d is the length of the polarimeter tube in dm (here 1 dm).

$$[\alpha]_D^T = \frac{100 \times \alpha}{c \times d}$$

The absolute configuration was determined by comparison of the optical rotation for compound **5** (see the corresponding section for details), and the absolute configurations of other compounds were assigned by analogy.

Synthesis and characterization of quinolones **1**.

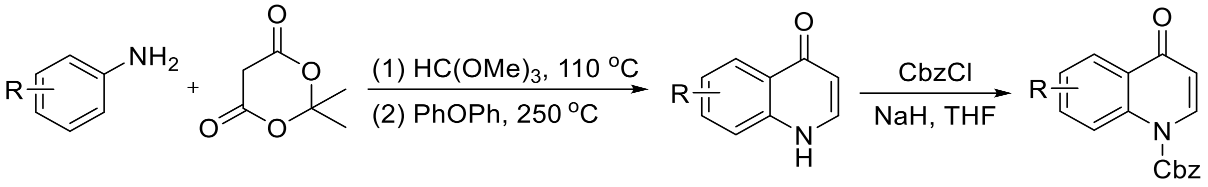

General procedure: Following a previously reported methodology,[1, 2] a solution of Meldrum's acid (1.08g, 7.5 mmol) and trimethyl orthoformate (13.7 mL) was heated to reflux at 110 °C for 2 h under argon (oil bath). The solution was cooled to room temperature, the corresponding aniline (5 mmol) was added and the mixture was heated to reflux at 110 °C for 2 h (oil bath). The precipitated product was washed with hexane (10 mL) and dried. Without further purification, the products (1.0 equiv) and diphenyl ether (10 mL) were heated at 250 °C for 1 h (oil bath). The solution was allowed to cool to room temperature and hexane (50 mL) was added. The precipitate formed was collected by filtration, washed with hexane (50 mL), and dried in vacuo.
After getting the crude 4(*H*)-quinolones, the next step is to protect the nitrogen with benzyl chloroformate. A solution of 4(1*H*)quinolone in THF (10 mL) was added to a suspension of NaH (346 mg, 8.65 mmol; 60 wt% in mineral oil) in THF (15 mL) at room temperature, and the resulting mixture was stirred for 15 min at 55 °C (oil bath). CbzCl (790 mg, 4.63 mmol) was then added dropwise, and the mixture was stirred for 21 h at room temperature. The reaction was quenched with water and extracted with Et_2O. The organic layer was dried over $MgSO_4$, filtered, and concentrated under vacuum. The residue was chromatographed on silica gel to afford the final compounds **1a,1b-m**.

1-Benzyloxycarbonyl-4-quinolone (1a)

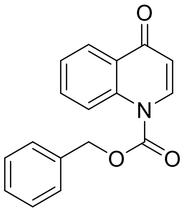

The general procedure was followed.
^{1}H NMR (400 MHz, $CDCl_3$) δ 8.67 (d, *J* = 8.9 Hz, 1H), 8.36 (d, *J* = 8.5 Hz, 2H), 7.71-7.61 (m, 1H), 7.53-7.39 (m, 6H), 6.25 (d, *J* = 8.6 Hz, 1H), 5.46 (s, 2H).
^{13}C {^{1}H} NMR (101 MHz, $CDCl_3$) δ 178.9, 151.3, 138.4, 138.2, 133.9, 132.9, 129.3, 129.0, 128.9, 126.5, 125.5, 120.0, 112.5, 110.0, 70.5.
Purification by flash column chromatography on silica gel (eluent, Pentane: Et_2O = 1:3). White solid, isolated yield: 80%.
The NMR data are in accordance with previously reported data.[2]

1-Benzyl-4-quinolone (1c)

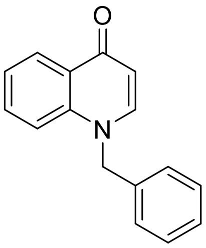

The general procedure was followed using BnBr instead of CbzCl.
^{1}H NMR (400 MHz, $CDCl_3$) δ 8.43 (d, *J* = 7.9 Hz, 1H), 7.62 (d, *J* = 7.6 Hz, 1H), 7.50 (t, *J* = 7.7 Hz, 1H), 7.40-7.19 (m, 5H), 7.11 (d, *J* = 6.8 Hz, 2H), 6.31 (d, *J* = 7.7 Hz, 1H), 5.30 (s, 2H).

---

[1] Li, M.; Li, L.; Ge, H. Direct C-3-Alkenylation of Quinolones via Palladium-Catalyzed C-H Functionalization. *Adv. Synth. Catal.* **2010**, *352*, 2445–2449.
[2] Guo, Y.; Harutyunyan, S. R. Highly Enantioselective Catalytic Addition of Grignard Reagents to N-Heterocyclic Acceptors. *Angew. Chem. Int. Ed.* **2019**, *58*, 12950–12954.

^{13}C {^{1}H} NMR (101 MHz, $CDCl_3$) δ 146.4, 142.7, 137.8, 134.8, 131.9, 130.9, 130.0, 129.6, 128.7, 126.4, 118.8, 113.0, 105.0, 59.1.
Purification by flash column chromatography on silica gel (eluent, Pentane: Et_2O = 1:1). White solid, isolated yield: 62%.
The NMR data are in accordance with previously reported data.[2]

1-Boc-4-quinolone (1d)

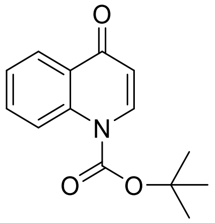

The general procedure was followed using Boc_2O instead of CbzCl.
^{1}H NMR (400 MHz, $CDCl_3$) δ 8.57 (d, *J* = 8.9 Hz, 1H), 8.36 (dd, *J* = 8.0, 1.8 Hz, 1H), 8.29 (d, *J* = 8.5 Hz, 1H), 7.64 (ddd, *J* = 8.9, 6.9, 1.8 Hz, 1H), 7.41 (td, *J* = 7.4, 6.9, 0.9 Hz, 1H), 6.25 (d, *J* = 8.5 Hz, 1H), 1.66 (s, 9H).
^{13}C {^{1}H} NMR (101 MHz, $CDCl_3$) δ 181.2, 152.0, 140.9, 140.7, 134.7, 128.8, 128.6, 127.3, 122.1, 114.0, 88.7, 30.1.
Purification by flash column chromatography on silica gel (eluent, Pentane: Et_2O = 3:1). White solid, isolated yield: 90%.
The NMR data are in accordance with previously reported data.[2]

1-Benzyloxycarbonyl-6-Methoxy-4-quinolone (1e)

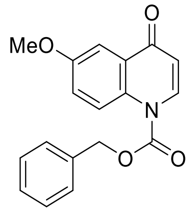

The general procedure was followed.
^{1}H NMR (400 MHz, $CDCl_3$) δ 8.55 (d, *J* = 9.6 Hz, 1H), 8.26 (d, *J* = 8.5 Hz, 1H), 7.68 (d, *J* = 3.1 Hz, 1H), 7.45-7.26 (m, 5H), 7.16 (dd, *J* = 9.6, 3.2 Hz, 1H), 6.15 (d, *J* = 8.5 Hz, 1H), 5.38 (s, 2H), 3.82 (s, 3H).
^{13}C {^{1}H} NMR (101 MHz, $CDCl_3$) δ 178.5, 156.9, 151.1, 137.6, 134.0, 132.6, 129.2, 128.9, 128.8, 127.8, 122.4, 121.7, 111.5, 106.1, 70.4, 55.6.
Purification by flash column chromatography on silica gel (eluent, Pentane: Et_2O = 1:2). White solid, isolated yield: 55%.
The NMR data are in accordance with previously reported data.[2]

1-Benzyloxycarbonyl-6-Trifloromethyl-4-quinolone (1f)

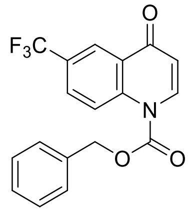

The general procedure was followed.
^{1}H NMR (400 MHz, $CDCl_3$) δ 8.83 (d, *J* = 9.2 Hz, 1H), 8.65 (s, 1H), 8.40 (d, *J* = 8.6 Hz, 1H), 7.86 (dd, *J* = 9.3, 1.8 Hz, 1H), 7.54-7.40 (m, 5H), 6.31 (d, *J* = 8.6 Hz, 1H), 5.49 (s, 2H).
^{13}C {^{1}H} NMR (101 MHz, $CDCl_3$) δ 178.0, 151.2, 140.6, 138.9, 133.7, 129.7, 129.2 (q, *J* = 6.5 Hz), 129.2, 129.1, 127.9 (q, *J* = 33.7 Hz), 126.6, 124.6 (q, *J* = 4.0 Hz), 123.7 (q, *J* = 272.2 Hz), 121.2, 113.2, 71.2.
^{19}F NMR (376 MHz, $CDCl_3$) δ -62.59.
Purification by flash column chromatography on silica gel (eluent, Pentane: Et_2O = 1:5). White solid, isolated yield: 73%.
The NMR data are in accordance with previously reported data.[2]

1-Benzyloxycarbonyl-6-bromo-4-quinolone (1g)

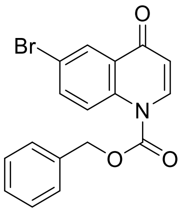

The general procedure was followed.

^{1}H NMR (400 MHz, $CDCl_3$) δ 8.59 (d, *J* = 9.3 Hz, 1H), 8.47 (s, 1H), 8.36 (d, *J* = 8.5 Hz, 1H), 7.72 (d, *J* = 9.3 Hz, 1H), 7.56-7.36 (m, 5H), 6.30-6.20 (m, 1H), 5.46 (s, 2H).

^{13}C {^{1}H} NMR (101 MHz, $CDCl_3$) δ 177.5, 151.0, 138.4, 137.2, 135.7, 133.7, 129.4, 129.1, 129.0, 128.9, 128.0, 122.1, 119.5, 112.6, 70.8.

Purification by flash column chromatography on silica gel (eluent, Pentane: Et_2O = 1:2). White solid, isolated yield: 45%.

The NMR data are in accordance with previously reported data.[2]

1-Benzyloxycarbonyl-6-methyl-4-quinolone (1h)

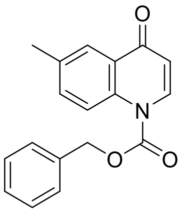

The general procedure was followed.

^{1}H NMR (400 MHz, $CDCl_3$) δ 8.56 (d, *J* = 9.0 Hz, 1H), 8.34 (d, *J* = 8.5 Hz, 1H), 8.15 (s, 1H), 7.55 – 7.31 (m, 6H), 6.23 (d, *J* = 8.5 Hz, 1H), 5.45 (s, 2H), 2.44 (s, 3H).

^{13}C {^{1}H} NMR (101 MHz, $CDCl_3$) δ 179.2, 151.4, 138.1, 136.5, 135.6, 134.3, 134.1, 129.4, 129.1, 128.9, 126.5, 126.1, 120.0, 112.4, 70.5, 20.9.

HRMS (ESI+, m/Z): calcd for $C_{18}H_{15}NO_3H$ $[M+H]^+$: 294.1125, found: 294.1128.

Purification by flash column chromatography on silica gel (eluent, Pentane: Et_2O = 1:1). Brownish oil, isolated yield: 46%.

1-Benzyloxycarbonyl-6-methoxycarbonyl-4-quinolone (1i)

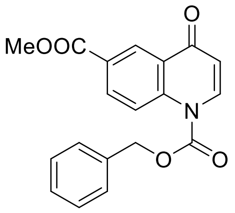

The general procedure was followed.

^{1}H NMR (400 MHz, $CDCl_3$) δ 9.00 (d, *J* = 2.2 Hz, 1H), 8.73 (d, *J* = 9.2 Hz, 1H), 8.36 (d, *J* = 8.6 Hz, 1H), 8.28 (dd, *J* = 9.2, 2.3 Hz, 1H), 7.52 – 7.38 (m, 5H), 6.29 (d, *J* = 8.6 Hz, 1H), 5.48 (s, 2H), 3.95 (s, 4H).

^{13}C {^{1}H} NMR (101 MHz, $CDCl_3$) δ 180.5, 168.1, 153.3, 143.4, 140.7, 135.8, 135.4, 131.6, 131.2, 131.1, 130.8, 129.4, 128.5, 122.5, 115.3, 73.1, 54.5.

HRMS (ESI+, m/Z): calcd for $C_{19}H_{15}NO_5H$ $[M+H]^+$: 338.1023, found: 338.1027.

Purification by flash column chromatography on silica gel (eluent, Pentane: Et_2O = 1:3). Pale yellow solid, isolated yield: 43%.

1-Benzyloxycarbonyl-6,7-methylenedioxy-4-quinolone (1j)

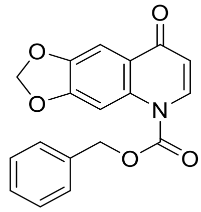

The general procedure was followed.
^{1}H NMR (400 MHz, $CDCl_3$) δ 8.28 (d, *J* = 8.5 Hz, 1H), 8.21 (s, 1H), 7.72 (s, 1H), 7.50 – 7.40 (m, 5H), 6.20 (d, *J* = 8.5 Hz, 1H), 6.09 (s, 2H), 5.44 (s, 2H).
^{13}C {^{1}H} NMR (101 MHz, $CDCl_3$) δ 177.9, 152.4, 151.5, 146.3, 137.3, 135.3, 134.0, 129.5, 129.1, 129.1, 129.0, 122.9, 112.2, 104.0, 102.4, 100.3, 70.7.
Purification by flash column chromatography on silica gel (eluent, Pentane: Et_2O = 1:3). White solid, isolated yield: 50%.
The NMR data are in accordance with previously reported data.[2]

1-Benzyloxycarbonyl-5,7-dimethyl-4-quinolone (1k)

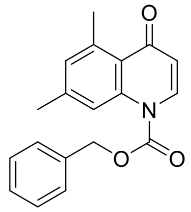

The general procedure was followed.
^{1}H NMR (400 MHz, $CDCl_3$) δ 8.24-8.11 (m, 2H), 7.53-7.34 (m, 5H), 6.99 (s, 1H), 6.10 (d, *J* = 8.5 Hz, 1H), 5.43 (s, 2H), 2.82 (s, 3H), 2.39 (s, 3H).
^{13}C {^{1}H} NMR (101 MHz, $CDCl_3$) δ 181.1, 151.7, 142.1, 140.9, 140.1, 136.3, 134.1, 130.1, 129.2, 128.9, 128.8, 123.2, 118.2, 114.2, 70.2, 23.9, 22.0.
Purification by flash column chromatography on silica gel (eluent, Pentane: Et_2O = 1:1). White solid, isolated yield: 41%.
The NMR data are in accordance with previously reported data.[2]

1-Benzyloxycarbonyl-8-fluoro-4-quinolone (1l)

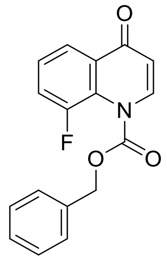

The general procedure was followed.
^{1}H NMR (400 MHz, $CDCl_3$) δ 8.95 (dd, *J* = 4.9, 1.2 Hz, 1H), 7.81 (dd, *J* = 8.3, 1.3 Hz, 1H), 7.55 – 7.32 (m, 8H), 5.35 (s, 2H).
^{13}C {^{1}H} NMR (101 MHz, $CDCl_3$) δ 158.1 (d, *J* = 257.0 Hz), 154.1 (d, *J* = 4.6 Hz), 152.1, 151.2 (d, *J* = 1.3 Hz), 140.4 (d, *J* = 12.7 Hz), 134.2, 129.2, 128.9, 128.8, 127.0 (d, *J* = 8.1 Hz), 123.6 (d, *J* = 2.3 Hz), 117.1 (d, *J* = 5.0 Hz), 114.4 (d, *J* = 18.8 Hz), 112.7 (d, *J* = 1.3 Hz), 71.3.
^{19}F NMR (376 MHz, $cdcl_3$) δ -124.10.
HRMS (ESI+, m/Z): calcd for $C_{17}H_{12}FNO_3H$ $[M+H]^+$: 298.0874, found: 298.0866.
Purification by flash column chromatography on silica gel (eluent, Pentane: Et_2O = 1:3). Pale yellow solid, isolated yield: 52%.

Synthesis and characterization of 4-oxo-2-alkynyl-dhydroquinolines 3.

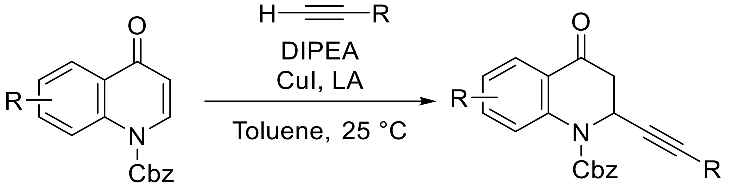

General procedure A (racemic): In a Schlenk tube (dried with a heat gun, ~650ºC), CuI (1.9 mg, 0.010 mmol) and the corresponding quinolone (0.100 mmol) were dissolved in 1.0 mL of dry Toluene. Then, DIPEA (28 µL, 0.160 mmol) and the corresponding terminal alkyne (0.130 mmol) were sequentially added dropwise. After stirring 5min at 25ºC, TBDMSOTf (28 µL, 0.120 mmol) was added and the reaction was stirred for 5h at the same temperature. The reaction was quenched by adding 1mL of 1M HCl (aq) solution and extracted with CH_2Cl_2 (3x5mL). The combined organic phases were dried over $MgSO_4$ and the volatiles were removed under reduced pressure. The purification was carried out by chromatographic column in silica gel using pentane/Et_2O (95:5 to 80:20) as eluent.

General procedure B (enantioselective): In a Schlenk tube (dried with a heat gun, ~650ºC), CuI (1.9 mg, 0.010 mmol) and (R,R)-Ph-BPE (5.6 mg, 0.011 mmol) were dissolved in 1.0 mL of dry toluene and the mixture was stirred for 15 min at 25ºC. Next, the corresponding quinolone (0.100 mmol) was added and the reaction was stirred for another 5 min at the same temperature. Then, DIPEA (28 µL, 0.160 mmol) and the corresponding terminal alkyne (0.130 mmol) were sequentially added dropwise. After stirring 5min at 25ºC, TBDMSOTf (28 µL, 0.120 mmol) were added and the reaction was stirred for 16h at the same temperature. The reaction was quenched by adding 1mL of 1M HCl (aq) solution and extracted with CH_2Cl_2 (3x5mL). The combined organic phases were dried over $MgSO_4$ and the volatiles were removed under reduced pressure. The purification was carried out by chromatographic column in silica gel using pentane/Et_2O (95:5 to 80:20) as eluent.

Optimization of base and solvent

The **general procedure A** (without ligand) was used.

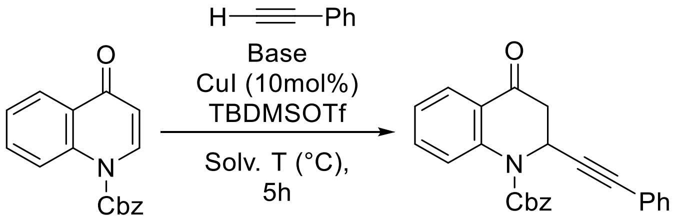

Entry	*Base*	*Solvent*	*Conv. (%)*
1	DIPEA	Toluene	> 99
2	DIPEA	THF	> 99
3	DIPEA	2-Me-THF	> 99
4	DIPEA	DCM	91
5	DIPEA	PhF	91
6	DIPEA	$PhCF_3$	95
7	Et_3N	Toluene	0
8	DBU	Toluene	0
9	DABCO	Toluene	0
10	$CsCO_3$	Toluene	0
11	Barton´s base	Toluene	<10

Benzyl 4-oxo-2-(phenylethynyl)-3,4-dihydroquinoline-1(2H)-carboxylate (3aa).

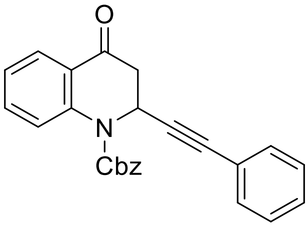

The **general procedure A** was followed, affording 31.0 mg (81%) of the product as a white solid.
Synthesis of 3aa, 1 mmol scale: In a Schlenk tube (dried with a heat gun, ~650ºC), CuI (19.0 mg, 0.10 mmol) was suspended in 10.0 mL of dry toluene and the mixture was stirred for 15 min at 25ºC. Next, **2a** (279.3 mg, 1.00 mmol) was added and the reaction was stirred for another 5 min at the same temperature. Then, DIPEA (279 µL, 1.60 mmol) and phenylacetylene (143 µL, 1.30 mmol) were sequentially added dropwise. After stirring 5 min at 25ºC, TBDMSOTf (276 µL, 1.20 mmol) were added and the resulting yellow solution was stirred for 5h at the same temperature. The reaction was quenched by adding 10 mL of 1M HCl (aq) solution and extracted with CH_2Cl_2 (3x20mL). The combined organic phases were dried over $MgSO_4$ and the volatiles were removed under reduced pressure. The purification was carried out by chromatographic column in silica gel using pentane/Et_2O (95:5 to 80:20) as eluent to afford 351 mg of **3aa** (92%).
^{1}H NMR (400 MHz, $CDCl_3$) δ 8.05 (dd, *J* = 7.9, 1.7 Hz, 1H), 7.85 (d, *J* = 8.4 Hz, 1H), 7.54 (ddd, *J* = 8.6, 7.3, 1.7 Hz, 1H), 7.45 – 7.34 (m, 5H), 7.25 – 7.15 (m, 6H), 6.08 (dd, *J* = 5.6, 2.1 Hz, 1H), 5.39 (d, *J* = 12.2 Hz, 1H), 5.29 (d, *J* = 12.2 Hz, 1H), 3.14 (dd, *J* = 17.1, 5.6 Hz, 1H), 2.96 (dd, *J* = 17.1, 2.1 Hz, 1H).
^{13}C {^{1}H} NMR (101 MHz, $CDCl_3$) δ 192.1, 153.2, 141.2, 135.6, 134.6, 131.9, 128.9, 128.8, 128.7, 128.4, 128.3, 127.2, 125.0, 124.7, 124.3, 121.9, 85.6, 85.0, 68.8, 47.4, 44.7.
HRMS (ESI+, m/Z): calcd for $C_{25}H_{19}NO_3H$ $[M+H]^+$: 381.1326, found: 381.1298.

Benzyl 2-((4-methoxyphenyl)ethynyl)-4-oxo-3,4-dihydroquinoline-1(2H)-carboxylate (3ab).

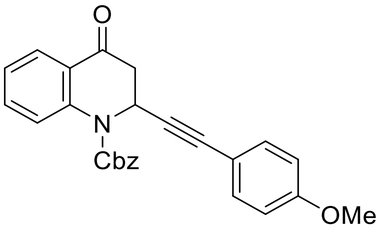

The **general procedure A** was followed, affording 35.8 mg (87%) of the product as a white solid.
^{1}H NMR (400 MHz, $CDCl_3$) δ 8.04 (dd, *J* = 7.9, 1.7 Hz, 1H), 7.85 (d, *J* = 8.4 Hz, 1H), 7.56 – 7.50 (m, 1H), 7.48 – 7.29 (m, 5H), 7.23 – 7.18 (m, 1H), 7.11 (d, *J* = 8.8 Hz, 2H), 6.72 (d, *J* = 8.8 Hz, 2H), 6.06 (dd, *J* = 5.5, 2.1 Hz, 1H), 5.38 (d, *J* = 12.2 Hz, 1H), 5.29 (d, *J* = 12.3 Hz, 1H), 3.75 (s, 3H), 3.12 (dd, *J* = 17.1, 5.5 Hz, 1H), 2.94 (dd, *J* = 17.1, 2.1 Hz, 1H).
^{13}C {^{1}H} NMR (101 MHz, $CDCl_3$) δ 192.3, 160.0, 153.2, 141.2, 135.6, 134.6, 133.4, 128.9, 128.7, 128.4, 127.2, 125.0, 124.7, 124.3, 114.0, 113.9, 85.0, 84.2, 68.7, 55.4, 47.5, 44.8.
HRMS (ESI+, m/Z): calcd for $C_{26}H_{21}NO_4H$ $[M+H]^+$: 412.1543, found: 412.1542.

Benzyl 4-oxo-2-(p-tolylethynyl)-3,4-dihydroquinoline-1(2H)-carboxylate (3ac).

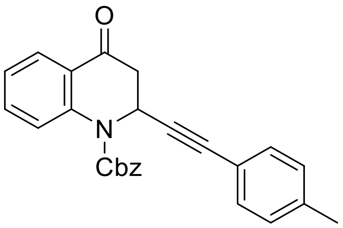

The **general procedure A** was followed, affording 32.7 mg (83%) of the product as a white solid.
^{1}H NMR (400 MHz, $CDCl_3$) δ 8.02 (dd, *J* = 7.9, 1.7 Hz, 1H), 7.83 (d, *J* = 8.4 Hz, 1H), 7.51 (ddd, *J* = 8.7, 7.2, 1.7 Hz, 1H), 7.44 – 7.32 (m, 5H), 7.19 (t, *J* = 7.5 Hz, 1H), 7.05 (d, *J* = 8.1 Hz, 2H), 6.98 (d, *J* = 8.0 Hz, 2H), 6.05 (dd, *J* = 5.5, 2.1 Hz, 1H), 5.37 (d, *J* = 12.3 Hz, 1H), 5.28 (d, *J* = 12.3 Hz, 1H), 3.11 (dd, *J* = 17.1, 5.5 Hz, 1H), 2.93 (dd, *J* = 17.1, 2.1 Hz, 1H), 2.26 (s, 3H).
^{13}C {^{1}H} NMR (101 MHz, $CDCl_3$) δ 194.7, 155.7, 143.7, 141.5, 138.1, 137.0, 134.3, 131.5, 131.4, 131.2, 130.9, 129.7, 127.5, 127.2, 126.8, 121.3, 87.6, 87.4, 71.2, 49.9, 47.3, 24.1.
HRMS (ESI+, m/Z): calcd for $C_{26}H_{21}NO_3H$ [M+H]$^+$: 396.1594, found: 396.1602.

Benzyl 2-((4-fluorophenyl)ethynyl)-4-oxo-3,4-dihydroquinoline-1(2H)-carboxylate (3ad).

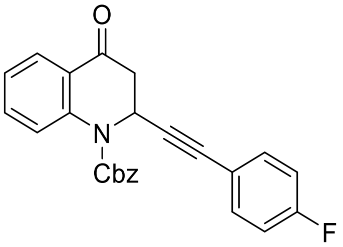

The **general procedure A** was followed, affording 32.7 mg (82%) of the product as a white solid.
^{1}H NMR (400 MHz, $CDCl_3$) δ 8.03 (d, *J* = 7.8 Hz, 1H), 7.83 (d, *J* = 8.4 Hz, 1H), 7.52 (t, *J* = 8.5 Hz, 1H), 7.44 – 7.32 (m, 5H), 7.20 (t, *J* = 7.6 Hz, 1H), 7.13 (dd, *J* = 8.6, 5.4 Hz, 2H), 6.87 (t, *J* = 8.6 Hz, 2H), 6.05 (dd, *J* = 5.6, 2.1 Hz, 1H), 5.37 (d, *J* = 12.2 Hz, 1H), 5.28 (d, *J* = 12.2 Hz, 1H), 3.12 (dd, *J* = 17.1, 5.6 Hz, 1H), 2.93 (dd, *J* = 17.1, 2.1 Hz, 1H).
^{13}C {^{1}H} NMR (101 MHz, $CDCl_3$) δ 192.0, 162.8 (d, *J* = 250.0 Hz), 153.2, 141.1, 135.6, 134.6, 133.9 (d, *J* = 8.4 Hz), 128.9, 128.7, 128.4, 127.3, 124.9, 124.8, 124.3, 118.0 (d, *J* = 3.5 Hz), 115.6 (d, *J* = 22.2 Hz), 85.3 (d, *J* = 1.4 Hz), 83.9, 68.8, 47.4, 44.7.
^{19}F NMR (376 MHz, $CDCl_3$) δ -110.15.
HRMS (ESI+, m/Z): calcd for $C_{25}H_{18}FNO_3H$ [M+H]$^+$: 400.1344, found: 400.1342.

Benzyl 4-oxo-2-((4-(trifluoromethyl)phenyl)ethynyl)-3,4-dihydroquinoline-1(2H)-carboxylate (3ae).

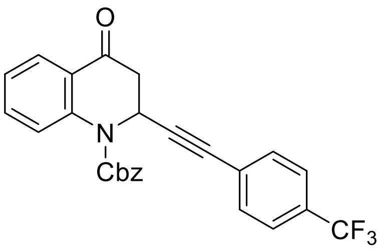

The **general procedure A** was followed, affording 34.2 mg (76%) of the product as a white solid.
^{1}H NMR (400 MHz, $CDCl_3$) δ 8.05 (dd, *J* = 7.9, 1.7 Hz, 1H), 7.85 (d, *J* = 8.5 Hz, 1H), 7.55 (td, *J* = 8.6, 7.9, 1.7 Hz, 1H), 7.49 – 7.36 (m, 6H), 7.30 – 7.20 (m, 4H), 6.10 (dd, *J* = 5.5, 2.1 Hz, 1H), 5.39 (d, *J* = 12.2 Hz, 1H), 5.30 (d, *J* = 12.2 Hz, 1H), 3.16 (dd, *J* = 17.2, 5.6 Hz, 1H), 2.96 (dd, *J* = 17.2, 2.1 Hz, 1H).
^{13}C {^{1}H} NMR (101 MHz, $CDCl_3$) δ 194.3, 155.6, 143.6, 138.0, 137.2, 134.7, 133.1 (q, *J* = 32.4 Hz), 131.4, 131.3, 131.0, 129.8, 127.7 (q, *J* = 3.8 Hz), 127.4, 127.3, 126.8, 126.3 (q, *J* = 272.2 Hz), 90.6, 86.1, 71.4, 49.8, 47.0.
^{19}F NMR (376 MHz, $CDCl_3$) δ -63.02.
HRMS (ESI+, m/Z): calcd for $C_{26}H_{18}F_3NO_3H$ [M+H]$^+$: 450.1312, found: 450.1293.

(*R*)–Benzyl 2-((3-methoxyphenyl)ethynyl)-4-oxo-3,4-dihydroquinoline-1(2H)-carboxylate (3af).

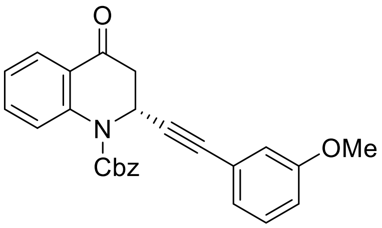

The **general procedure A** was followed, affording 32.5 mg (79%) of the product as a white solid.
The **general procedure B** was followed, affording 35.3 mg (86%) of the product as a white solid. 68% ee.
^{1}H NMR (400 MHz, $CDCl_3$) δ 8.04 (dd, *J* = 7.8, 1.5 Hz, 1H), 7.85 (d, *J* = 8.3 Hz, 1H), 7.57 – 7.51 (m, 1H), 7.46 – 7.32 (m, 5H), 7.24 – 7.19 (m, 1H), 7.10 (t, *J* = 7.9 Hz, 1H), 6.84 – 6.75 (m, 2H), 6.69 (s, 1H), 6.07 (dd, *J* = 5.5, 2.1 Hz, 1H), 5.38 (d, *J* = 12.2 Hz, 1H), 5.29 (d, *J* = 12.2 Hz, 1H), 3.72 (s, 3H), 3.14 (dd, *J* = 17.2, 5.6 Hz, 1H), 2.95 (dd, *J* = 17.2, 2.1 Hz, 1H).
^{13}C {^{1}H} NMR (101 MHz, $CDCl_3$) δ 192.1, 159.3, 153.2, 141.2, 135.6, 134.6, 129.4, 128.9, 128.7, 128.4, 127.3, 125.0, 124.8, 124.5, 124.3, 122.9, 116.8, 115.4, 85.4, 84.9, 68.8, 55.4, 47.4, 44.7.
HRMS (ESI+, m/Z): calcd for $C_{26}H_{21}NO_4H$ $[M+H]^+$: 412.1543, found: 412.1534.
HPLC analysis: Chiralcel-OD-H, n-heptane/i-PrOH 90:10, 0.5 mL/min, 40 °C, detection at 280 nm. Retention time (min): 27.22 (minor) and 32.01 (major).

<Chromatogram>

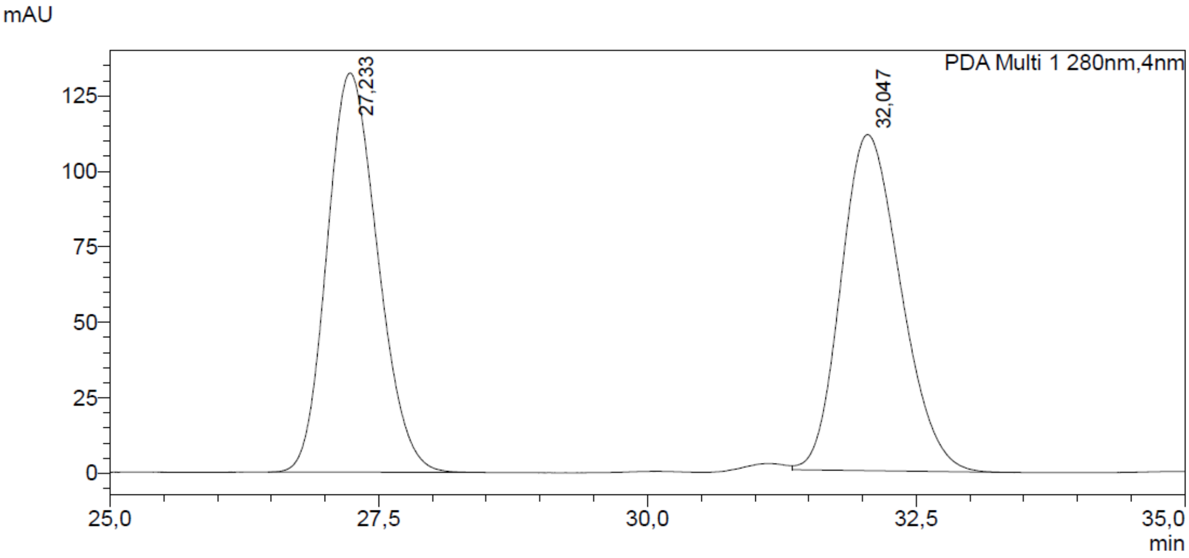

<Peak Table>

PDA Ch1 280nm

Peak#	Ret. Time	Area	Area%
1	27,233	4339983	50,155
2	32,047	4313134	49,845
Total		8653117	100,000

<Chromatogram>

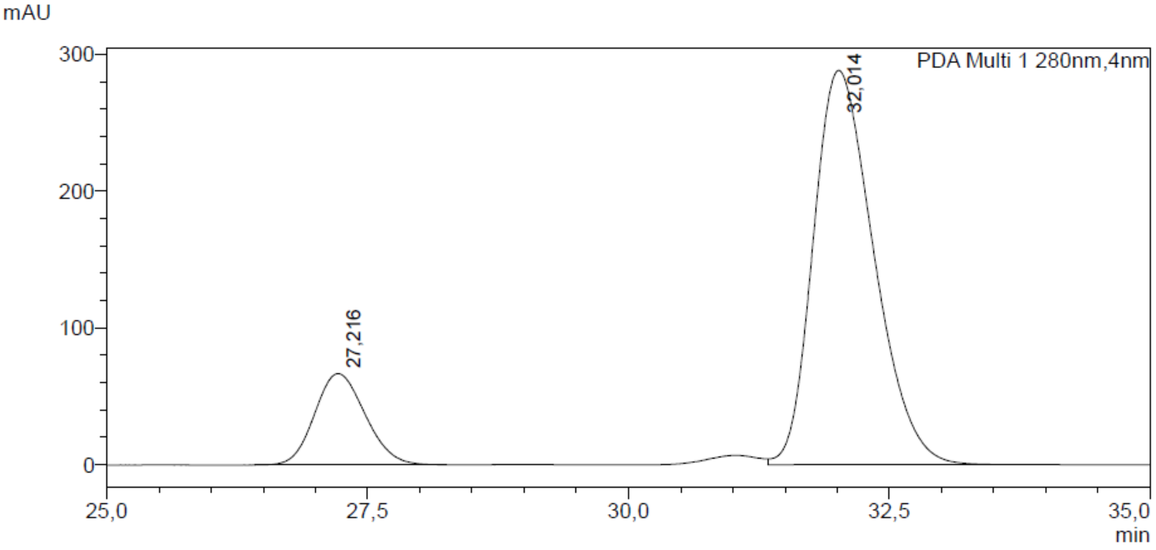

<Peak Table>

PDA Ch1 280nm

Peak#	Ret. Time	Area	Area%
1	27,216	2243661	16,178
2	32,014	11624914	83,822
Total		13868575	100,000

Peak# : 1
Retention Time : 27,233 min
Compound Name :
Spectrum Operation:

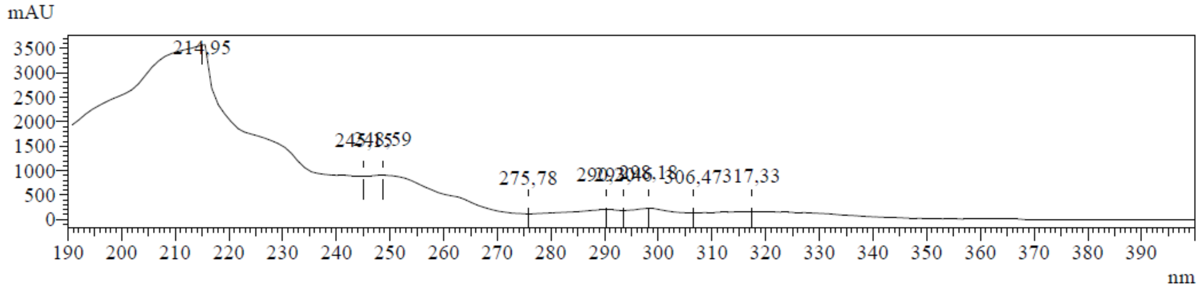

Peak# : 2
Retention Time : 32,047 min
Compound Name :
Spectrum Operation:

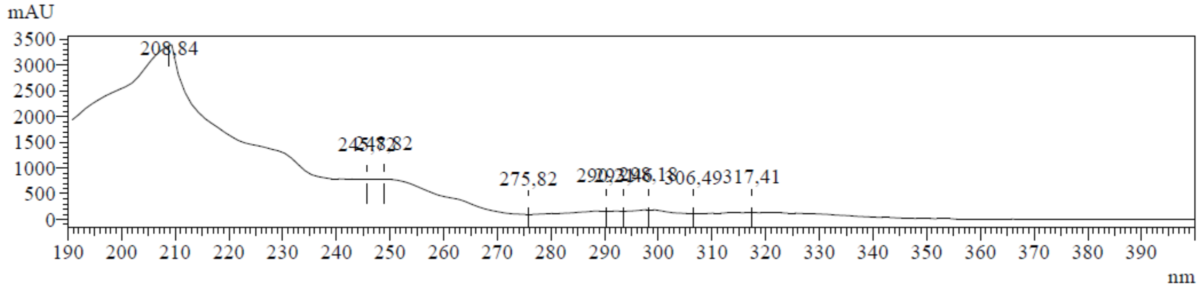

(*R*)–Benzyl 4-oxo-2-(m-tolylethynyl)-3,4-dihydroquinoline-1(2H)-carboxylate (3ag).

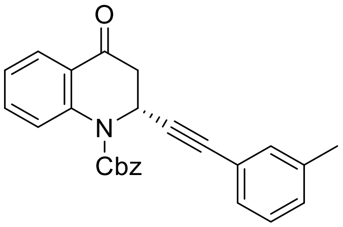

The **general procedure A** was followed, affording 29.3 mg (74%) of the product as a white solid.
The **general procedure B** was followed, affording 32.9 mg (83%) of the product as a white solid. 82% ee.
^{1}H NMR (400 MHz, $CDCl_3$) δ 8.04 (dd, *J* = 7.8, 1.7 Hz, 1H), 7.85 (d, *J* = 8.8 Hz, 1H), 7.57 – 7.50 (m, 1H), 7.45 – 7.34 (m, 5H), 7.22 (t, *J* = 7.7 Hz, 1H), 7.11 – 7.04 (m, 2H), 7.02 – 6.95 (m, 2H), 6.07 (dd, *J* = 5.6, 2.1 Hz, 1H), 5.38 (d, *J* = 12.2 Hz, 1H), 5.30 (d, *J* = 0.7 Hz, 1H), 3.13 (dd, *J* = 17.1, 5.5 Hz, 1H), 2.95 (dd, *J* = 17.1, 2.1 Hz, 1H), 2.23 (s, 3H).
^{13}C {^{1}H} NMR (101 MHz, $CDCl_3$) δ 192.1, 153.2, 141.2, 138.0, 135.6, 134.6, 132.5, 129.7, 129.0, 128.9, 128.7, 128.4, 128.2, 127.2, 125.0, 124.7, 124.3, 121.7, 85.2, 85.1, 68.8, 47.4, 44.8, 21.2.
HRMS (ESI+, m/Z): calcd for $C_{26}H_{21}NO_3H$ $[M+H]^+$: 396.1594, found: 396.1593.
HPLC analysis: Chiralcel-OD-H, n-heptane/i-PrOH 90:10, 0.5 mL/min, 40 °C, detection at 315 nm. Retention time (min): 20.31 (minor) and 24.42 (major).

<Chromatogram>

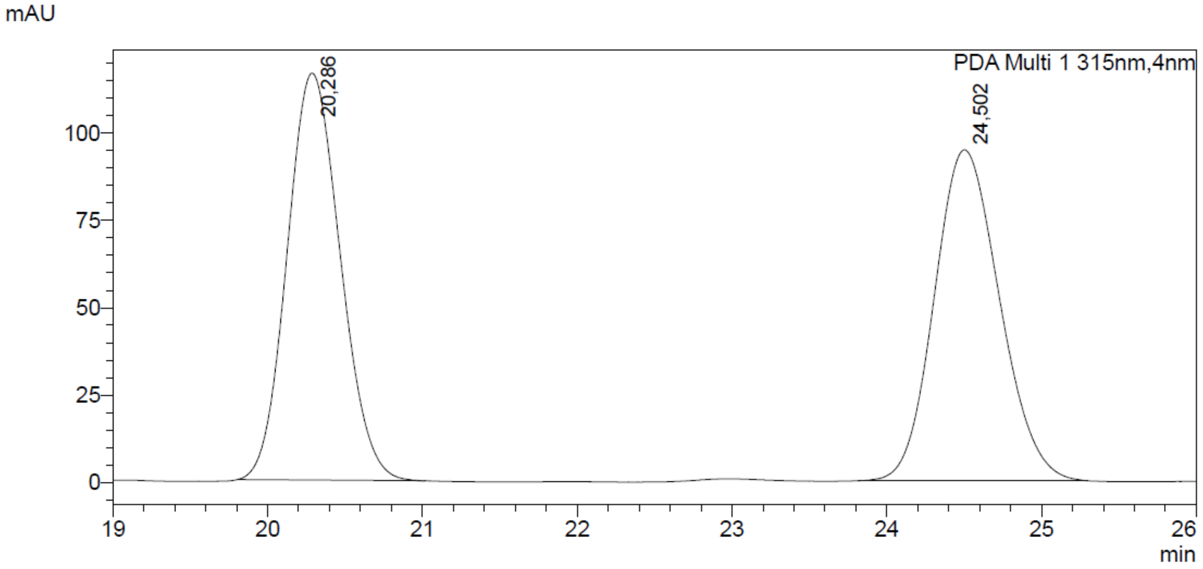

<Peak Table>

PDA Ch1 315nm

Peak#	Ret. Time	Area	Area%
1	20,286	2781596	50,091
2	24,502	2771459	49,909
Total		5553055	100,000

<Chromatogram>

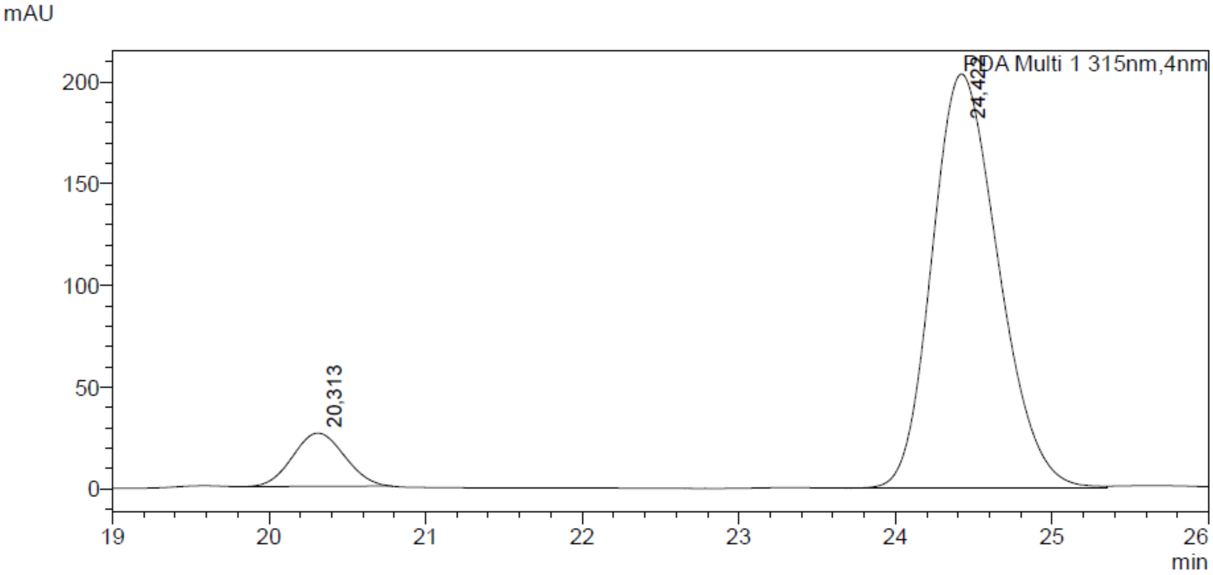

<Peak Table>

PDA Ch1 315nm

Peak#	Ret. Time	Area	Area%
1	20,313	601295	9,114
2	24,422	5995928	90,886
Total		6597223	100,000

Peak# : 1
Retention Time : 20,286 min
Compound Name :
Spectrum Operation:

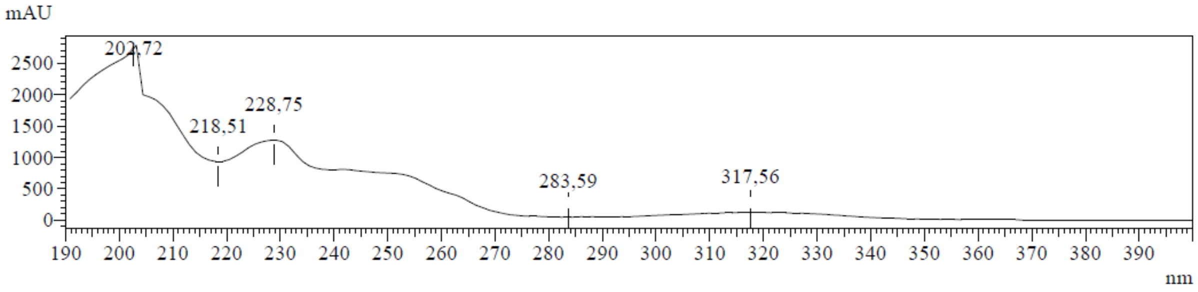

Peak# : 2
Retention Time : 24,502 min
Compound Name :
Spectrum Operation:

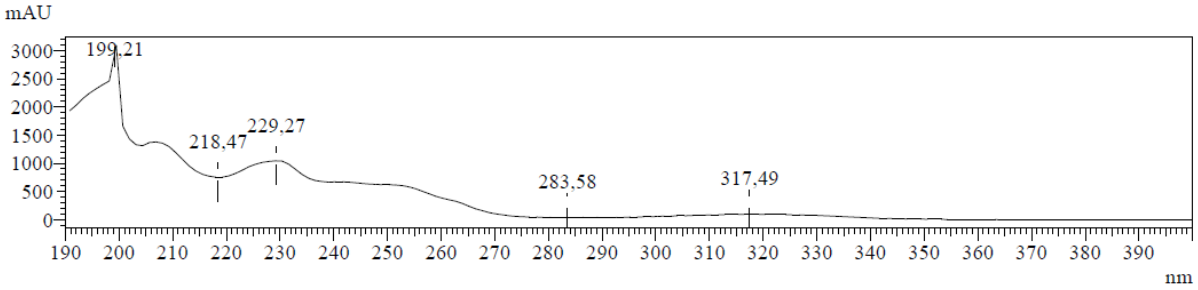

Benzyl 2-((3-chlorophenyl)ethynyl)-4-oxo-3,4-dihydroquinoline-1(2H)-carboxylate (3ah).

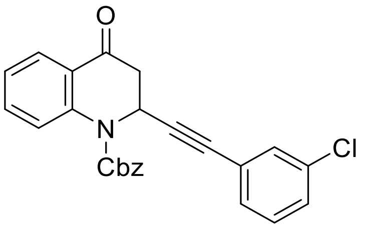

The **general procedure A** was followed, affording 33.6 mg (81%) of the product as a white solid.
^{1}H NMR (400 MHz, $CDCl_3$) δ 8.05 (dd, *J* = 7.9, 1.7 Hz, 1H), 7.85 (d, *J* = 8.5 Hz, 1H), 7.55 (ddd, *J* = 8.6, 7.2, 1.7 Hz, 1H), 7.46 – 7.34 (m, 5H), 7.25 – 7.20 (m, 2H), 7.17 – 7.10 (m, 2H), 7.05 (d, *J* = 7.7 Hz, 1H), 6.08 (dd, *J* = 5.6, 2.1 Hz, 1H), 5.38 (d, *J* = 12.2 Hz, 1H), 5.30 (d, *J* = 12.2 Hz, 1H), 3.14 (dd, *J* = 17.2, 5.6 Hz, 1H), 2.94 (dd, *J* = 17.2, 2.1 Hz, 1H).
^{13}C {^{1}H} NMR (101 MHz, $CDCl_3$) δ 191.9, 153.1, 141.1, 135.5, 134.7, 134.2, 131.8, 130.1, 129.6, 129.1, 128.9, 128.8, 128.5, 127.3, 124.8, 124.2, 123.6, 86.8, 83.5, 68.9, 47.3, 44.5.
HRMS (APCI+, m/Z): calcd for $C_{17}H_{11}ClNO$ $[M]^+$: 280.0524, found: 280.0519. The HRMS did not successfully afford the exact mass for the title compound. The fragment resulting from the loss of Cbz was found instead.

Benzyl 4-oxo-2-((3-(trifluoromethyl)phenyl)ethynyl)-3,4-dihydroquinoline-1(2H)-carboxylate (3ai).

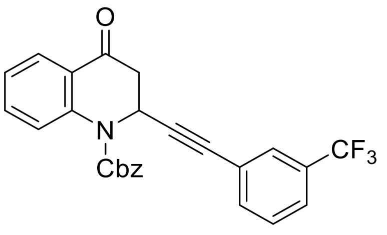

The **general procedure A** was followed, affording 37.8 mg (84%) of the product as a white solid.
^{1}H NMR (400 MHz, $CDCl_3$) δ 8.05 (dd, *J* = 7.8, 1.7 Hz, 1H), 7.86 (d, *J* = 8.5 Hz, 1H), 7.56 (ddd, *J* = 8.6, 7.3, 1.7 Hz, 1H), 7.52 – 7.48 (m, 1H), 7.46 – 7.30 (m, 8H), 7.26 – 7.21 (m, 1H), 6.10 (dd, *J* = 5.7, 2.1 Hz, 1H), 5.39 (d, *J* = 12.2 Hz, 1H), 5.30 (d, *J* = 12.2 Hz, 1H), 3.16 (dd, *J* = 17.2, 5.6 Hz, 1H), 2.96 (dd, *J* = 17.2, 2.1 Hz, 1H).
^{13}C {^{1}H} NMR (101 MHz, $CDCl_3$) δ 191.8, 153.1, 141.1, 135.5, 135.1 (q, *J* = 1.3 Hz), 134.8, 131.0 (q, *J* = 32.9 Hz), 128.9, 128.9, 128.8, 128.7 (q, *J* = 3.8 Hz), 128.5, 127.3, 125.4 (q, *J* = 3.7 Hz), 124.9, 124.8, 124.2, 122.8, 87.2, 83.4, 68.9, 47.3, 44.5.
^{19}F NMR (376 MHz, $CDCl_3$) δ -63.07.
HRMS (APCI+, m/Z): calcd for $C_{18}H_{11}F_3NO$ $[M]^+$: 314.0787, found: 314.0785. The HRMS did not successfully afford the exact mass for the title compound. The fragment resulting from the loss of Cbz was found instead.
(*R*)–Benzyl 4-oxo-2-(o-tolylethynyl)-3,4-dihydroquinoline-1(2H)-carboxylate (3aj).

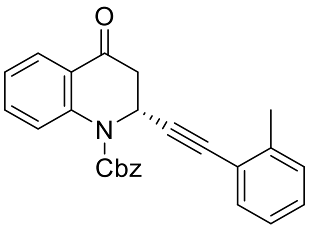

The **general procedure A** was followed, affording 34.1 mg (86%) of the product as a white solid.
The **general procedure B** was followed, affording 32.8 mg (83%) of the product as a white solid. 54% ee.
^{1}H NMR (400 MHz, $CDCl_3$) δ 8.04 (dd, *J* = 7.8, 1.7 Hz, 1H), 7.84 (d, *J* = 9.0 Hz, 1H), 7.53 (td, *J* = 8.7, 8.1, 1.8 Hz, 1H), 7.45 – 7.34 (m, 5H), 7.24 – 7.12 (m, 3H), 7.07 – 7.00 (m, 2H), 6.09 (dd, *J* = 5.5, 2.1 Hz, 1H), 5.39 (d, *J* = 12.2 Hz, 1H), 5.30 (d, *J* = 12.2 Hz, 1H), 3.16 (dd, *J* = 17.1, 5.4 Hz, 1H), 2.96 (dd, *J* = 17.2, 2.1 Hz, 1H), 1.93 (s, 3H).
^{13}C {^{1}H} NMR (101 MHz, $CDCl_3$) δ 192.0, 153.0, 141.2, 140.6, 135.5, 134.4, 131.8, 129.3, 128.7, 128.7, 128.6, 128.3, 127.1, 125.4, 125.0, 124.6, 124.2, 121.5, 89.4, 83.9, 68.6, 47.5, 44.9, 20.1.

HRMS (APCI+, m/Z): calcd for $C_{18}H_{14}NO$ $[M]^+$: 260.1070, found: 260.1066. The HRMS did not successfully afford the exact mass for the title compound. The fragment resulting from the loss of Cbz was found instead.
HPLC analysis: Chiralcel-OD-H, n-heptane/i-PrOH 90:10, 0.5 mL/min, 40 °C, detection at 315 nm. Retention time (min): 20.75 (minor) and 23.04 (major).

<Chromatogram>

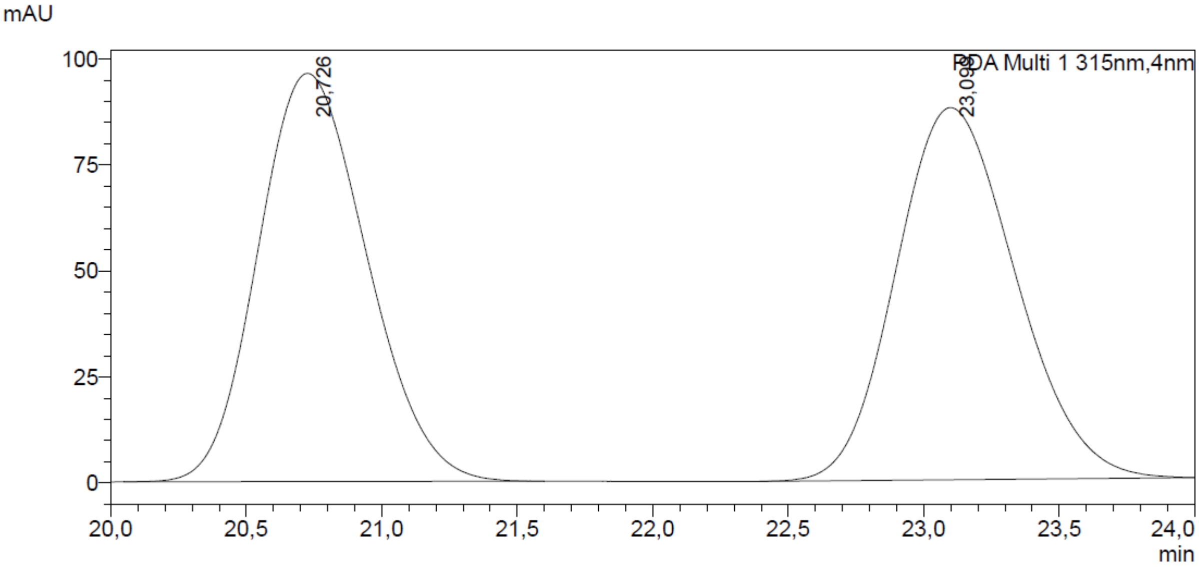

<Peak Table>

PDA Ch1 315nm

Peak#	Ret. Time	Area	Area%
1	20,726	2685189	49,949
2	23,099	2690706	50,051
Total		5375895	100,000

<Chromatogram>

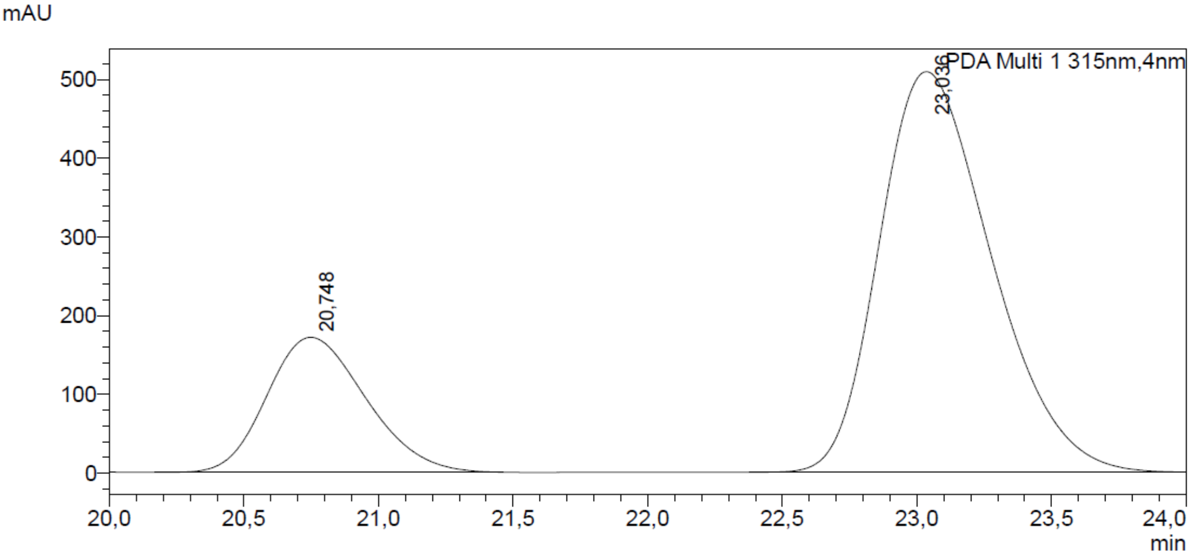

<Peak Table>

PDA Ch1 315nm

Peak#	Ret. Time	Area	Area%
1	20,748	4384300	22,948
2	23,036	14720936	77,052
Total		19105236	100,000

Peak# : 1
Retention Time : 20.726 min
Compound Name :
Spectrum Operation:

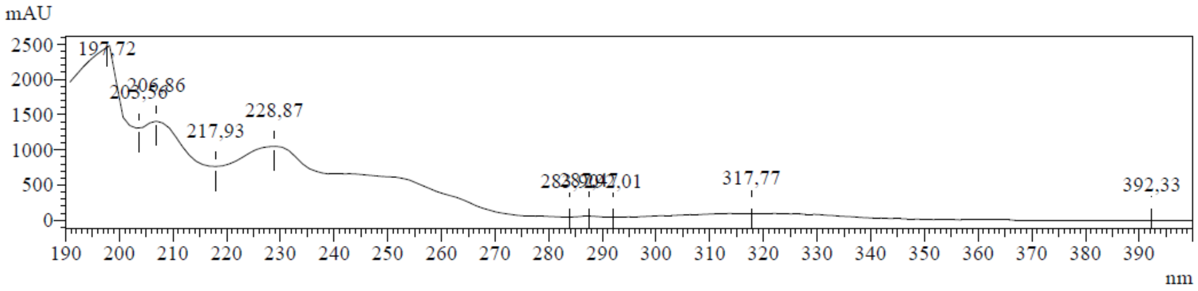

Peak# : 2
Retention Time : 23.099 min
Compound Name :
Spectrum Operation:

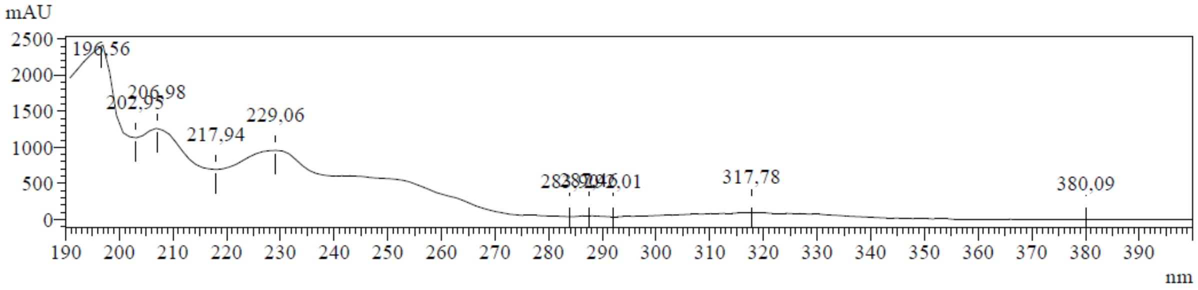

(*R*)–Benzyl 4-oxo-2-(thiophen-3-ylethynyl)-3,4-dihydroquinoline-1(2H)-carboxylate (3ak).

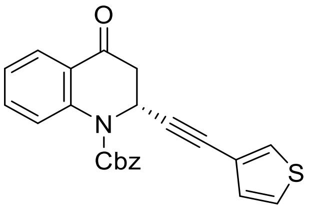

The **general procedure A** was followed, affording 33.1 mg (85%) of the product as a light brown solid.
The **general procedure B** was followed, affording 32.9 mg (85%) of the product as a light brown solid. 50% ee.
^{1}**H NMR** (400 MHz, $CDCl_3$) δ 8.04 (dd, *J* = 7.8, 1.7 Hz, 1H), 7.85 (d, *J* = 8.4 Hz, 1H), 7.54 (ddd, *J* = 8.6, 7.3, 1.7 Hz, 1H), 7.47 – 7.31 (m, 5H), 7.26 – 7.19 (m, 2H), 7.15 (dd, *J* = 5.0, 3.0 Hz, 1H), 6.87 (dd, *J* = 5.0, 1.2 Hz, 1H), 6.07 (dd, *J* = 5.6, 2.1 Hz, 1H), 5.38 (d, *J* = 12.2 Hz, 1H), 5.29 (d, *J* = 12.3 Hz, 1H), 3.13 (dd, *J* = 17.2, 5.6 Hz, 1H), 2.94 (dd, *J* = 17.1, 2.1 Hz, 1H).
^{13}**C {^{1}H} NMR** (101 MHz, $CDCl_3$) δ 192.1, 153.2, 141.2, 135.6, 134.6, 130.0, 129.8, 128.9, 128.7, 128.4, 127.3, 125.4, 124.9, 124.7, 124.3, 120.9, 85.2, 80.1, 68.8, 47.4, 44.7.
HRMS (ESI+, m/Z): calcd for $C_{23}H_{17}NO_3SH$ $[M+H]^+$: 388.1002, found: 388.1015.
HPLC analysis: Chiralcel-OD-H, n-heptane/i-PrOH 90:10, 0.5 mL/min, 40 °C, detection at 315 nm. Retention time (min): 29.57 (minor) and 32.31 (major).

<Chromatogram>

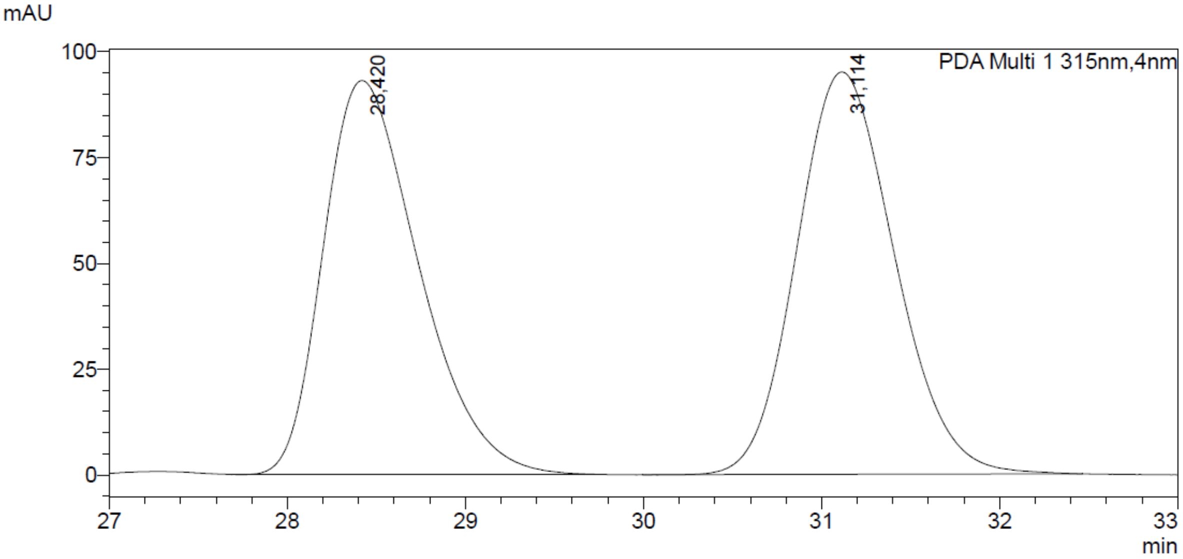

<Peak Table>

PDA Ch1 315nm

Peak#	Ret. Time	Area	Area%
1	28,420	3468241	48,847
2	31,114	3632034	51,153
Total		7100275	100,000

<Chromatogram>

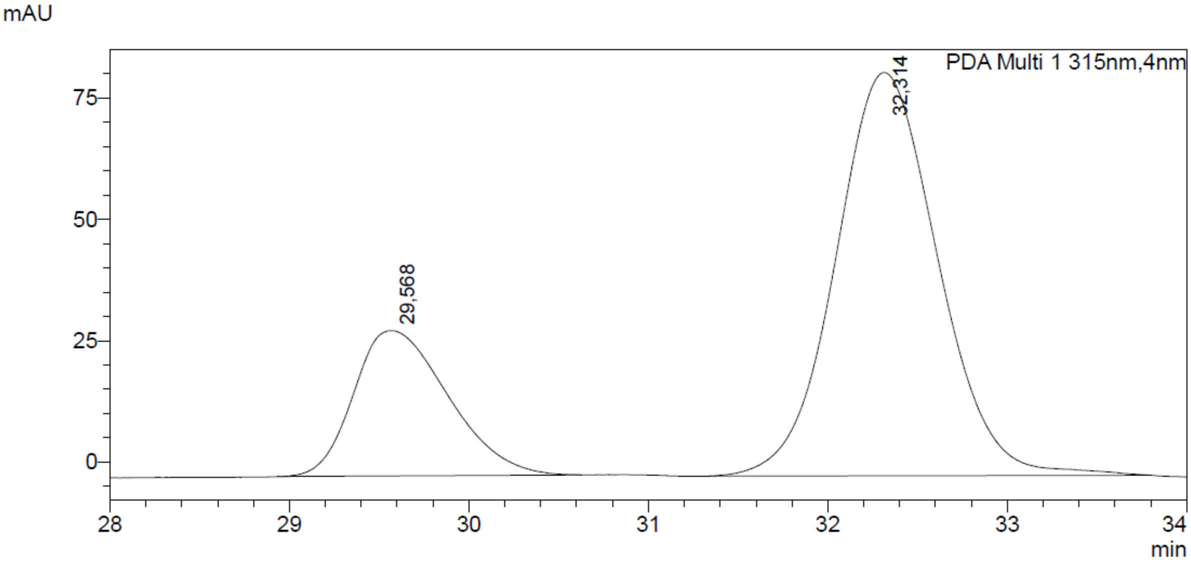

<Peak Table>

PDA Ch1 315nm

Peak#	Ret. Time	Area	Area%
1	29,568	1094190	24,996
2	32,314	3283347	75,004
Total		4377536	100,000

Peak# : 1
Retention Time : 28,420 min
Compound Name :
Spectrum Operation:

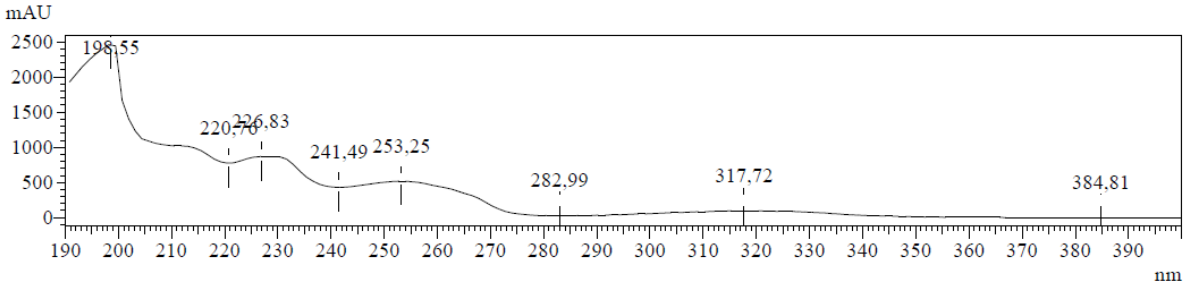

Peak# : 2
Retention Time : 31,114 min
Compound Name :
Spectrum Operation:

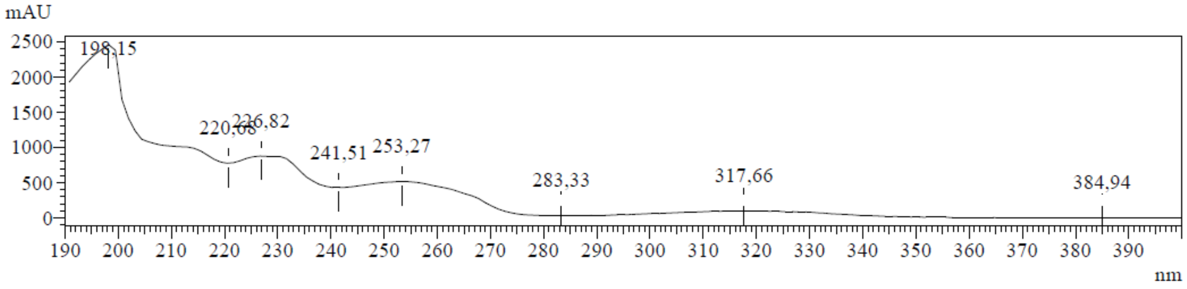

Benzyl 2-(cyclopropylethynyl)-4-oxo-3,4-dihydroquinoline-1(2H)-carboxylate (3al).

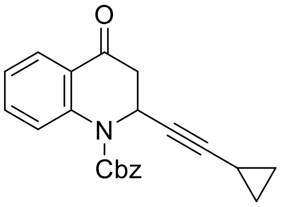

The **general procedure A** was followed, affording 25.6 mg (74%) of the product as a white solid.

^{1}H NMR (400 MHz, $CDCl_3$) δ 7.99 (d, *J* = 7.8 Hz, 1H), 7.80 (d, *J* = 8.4 Hz, 1H), 7.51 (t, *J* = 8.5 Hz, 1H), 7.42 – 7.30 (m, 5H), 7.18 (t, *J* = 7.5 Hz, 1H), 5.79 – 5.74 (m, 1H), 5.33 (d, *J* = 12.2 Hz, 1H), 5.23 (d, *J* = 12.3 Hz, 1H), 2.99 (dd, *J* = 17.0, 5.5 Hz, 1H), 2.78 (dd, *J* = 17.0, 2.1 Hz, 1H), 1.04 – 0.94 (m, 1H), 0.64 – 0.55 (m, 2H), 0.44 – 0.33 (m, 2H).

^{13}C {^{1}H} NMR (101 MHz, $CDCl_3$) δ 192.4, 153.2, 141.2, 135.7, 134.4, 128.8, 128.7, 128.4, 127.1, 125.0, 124.5, 124.3, 89.1, 71.6, 68.6, 47.1, 45.0, 8.4, -0.7.

HRMS (ESI+, m/Z): calcd for $C_{22}H_{18}NO_3$ $[M+H]^-$: 344.1292, found: 344.1296.

Benzyl 2-(4-methylpent-1-yn-1-yl)-4-oxo-3,4-dihydroquinoline-1(2H)-carboxylate (3am).

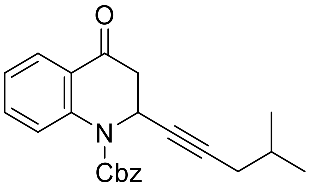

The **general procedure A** was followed, affording 23.8 mg (66%) of the product as a colorless oil.

^{1}H NMR (400 MHz, $CDCl_3$) δ 8.02 (dd, *J* = 7.8, 1.7 Hz, 1H), 7.81 (d, *J* = 8.4 Hz, 1H), 7.54 – 7.49 (m, 1H), 7.45 – 7.31 (m, 5H), 7.22 – 7.16 (m, 1H), 5.81 (dd, *J* = 5.3, 2.1 Hz, 1H), 5.35 (d, *J* = 12.3 Hz, 1H), 5.26 (d, *J* = 12.3 Hz, 1H), 3.03 (dd, *J* = 17.0, 5.4 Hz, 1H), 2.82 (dd, *J* = 17.0, 2.1 Hz, 1H), 1.90 – 1.83 (m, 2H), 1.57 – 1.51 (m, 1H), 0.70 – 0.61 (m, 6H).

^{13}C {^{1}H} NMR (101 MHz, $CDCl_3$) δ 192.5, 153.2, 141.3, 135.7, 134.4, 128.8, 128.6, 128.4, 127.1, 125.1, 124.6, 124.3, 85.0, 77.6, 68.6, 47.1, 45.2, 27.7, 27.6, 21.6, 21.6.

HRMS (APCI+, m/Z): calcd for $C_{15}H_{16}NO$ $[M]^+$: 226.1226, found: 226.1223. The HRMS did not successfully afford the exact mass for the title compound. The fragment resulting from the loss of Cbz was found instead.

Benzyl 2-(3-methoxy-3-oxoprop-1-yn-1-yl)-4-oxo-3,4-dihydroquinoline-1(2H)-carboxylate (3an).

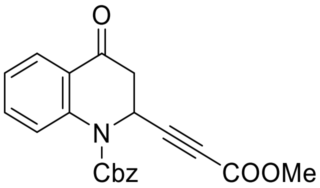

Slightly modification of the general procedure A was used. After stirring the reaction for the required time, TBAF was added (1M in THF, 0.1mL), and the reaction was stirred for another 5h at 25ºC. Then, the reaction was quenched by adding 1mL of 1M HCl (aq) solution, and extracted with CH_2Cl_2 (3x5mL). The combined organic phases were dried over $MgSO_4$ and the volatiles removed under reduced pressure. The purification was carried out by chromatographic column in silica gel using pentane/Et_2O (9:1) as eluent.

The **modified general procedure A** was followed, affording 29.8 mg (82%) of the product as a white solid.

^{1}H NMR (400 MHz, $CDCl_3$) δ 8.03 (dd, *J* = 7.8, 1.7 Hz, 1H), 7.81 (d, *J* = 8.4 Hz, 1H), 7.59 – 7.53 (m, 1H), 7.43 – 7.33 (m, 5H), 7.26 – 7.21 (m, 1H), 6.03 (dd, *J* = 6.0, 2.0 Hz, 1H), 5.36 (d, *J* = 12.2 Hz, 1H), 5.26 (d, *J* = 12.1 Hz, 1H), 3.66 (s, 3H), 3.12 (dd, *J* = 17.4, 6.0 Hz, 1H), 2.91 (dd, *J* = 17.4, 2.0 Hz, 1H).

^{13}C {^{1}H} NMR (101 MHz, $CDCl_3$) δ 190.7, 153.1, 152.9, 140.7, 135.2, 135.0, 128.9, 128.9, 128.5, 127.5, 125.2, 124.5, 124.3, 83.5, 75.6, 69.2, 53.0, 46.5, 43.4.

HRMS (ESI+, m/Z): calcd for $C_{21}H_{17}NO_5Na$ $[M+Na]^+$: 386.0999, found: 386.0998.

Benzyl 6-methoxy-4-oxo-2-(m-tolylethynyl)-3,4-dihydroquinoline-1(2H)-carboxylate (3eg).

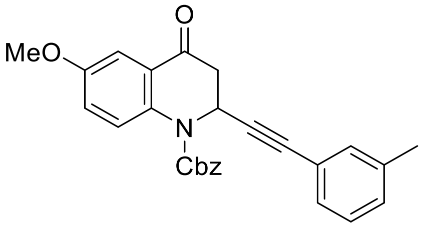

The **general procedure A** was followed, affording 33.6 mg (79%) of the product as a white solid.
^{1}H NMR (400 MHz, $CDCl_3$) δ 7.74 (d, *J* = 9.3 Hz, 1H), 7.49 (d, *J* = 3.1 Hz, 1H), 7.46 – 7.30 (m, 5H), 7.14 – 6.98 (m, 5H), 6.05 (dd, *J* = 5.7, 1.9 Hz, 1H), 5.36 (d, *J* = 12.3 Hz, 1H), 5.27 (d, *J* = 12.3 Hz, 1H), 3.84 (s, 3H), 3.12 (dd, *J* = 17.2, 5.5 Hz, 1H), 2.93 (dd, *J* = 17.3, 2.1 Hz, 1H), 2.24 (s, 3H).
^{13}C {^{1}H} NMR (151 MHz, $CDCl_3$) δ 192.0, 156.3, 153.1, 137.9, 135.6, 134.7, 132.3, 129.5, 128.9, 128.7, 128.5, 128.2, 128.0, 125.9, 125.6, 122.5, 121.6, 108.6, 85.1, 84.8, 68.5, 55.6, 47.3, 44.6, 21.1.
HRMS (APCI+, m/Z): calcd for $C_{19}H_{16}NO_2$ $[M]^+$: 290.1176, found: 290.1173. The HRMS did not successfully afford the exact mass for the title compound. The fragment resulting from the loss of Cbz was found instead.
Benzyl 4-oxo-2-(m-tolylethynyl)-6-(trifluoromethyl)-3,4-dihydroquinoline-1(2H)-carboxylate (3fg).

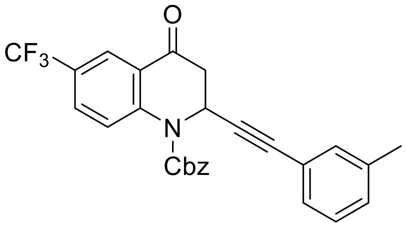

The **general procedure A** was followed, affording 38.5 mg (83%) of the product as a colorless oil.
^{1}H NMR (400 MHz, $CDCl_3$) δ 8.33 (d, *J* = 2.3 Hz, 1H), 8.08 (d, *J* = 8.9 Hz, 1H), 7.75 (dd, *J* = 8.9, 2.4 Hz, 1H), 7.48 – 7.34 (m, 5H), 7.14 – 7.04 (m, 2H), 7.05 – 6.96 (m, 2H), 6.10 (dd, *J* = 5.5, 2.2 Hz, 1H), 5.41 (d, *J* = 12.1 Hz, 1H), 5.33 (d, *J* = 12.1 Hz, 1H), 3.15 (dd, *J* = 17.1, 5.5 Hz, 1H), 3.01 (dd, *J* = 17.1, 2.3 Hz, 1H), 2.24 (s, 3H).
^{13}C {^{1}H} NMR (101 MHz, $CDCl_3$) δ 191.1, 153.2, 144.2, 138.5, 135.5, 132.9, 131.2 (q, *J* = 3.4 Hz), 130.3, 129.3, 129.3, 129.3, 128.9, 128.6, 127.0 (q, *J* = 33.4 Hz), 125.1 (q, *J* = 3.7 Hz), 124.8, 124.8, 124.0 (q, *J* = 272.0 Hz), 121.6, 86.0, 84.7, 69.6, 47.7, 44.7, 21.5.
^{19}F NMR (376 MHz, $CDCl_3$) δ -62.66.
HRMS (ESI+, m/Z): calcd for $C_{27}H_{20}F_3NO_3H$ $[M+H]^+$: 464.1468, found: 464.1462.
Benzyl 6-bromo-4-oxo-2-(m-tolylethynyl)-3,4-dihydroquinoline-1(2H)-carboxylate (3gg).

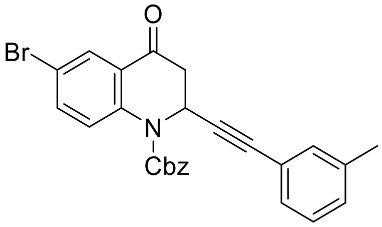

The **general procedure A** was followed, affording 38.9 mg (82%) of the product as a white solid.
^{1}H NMR (400 MHz, $CDCl_3$) δ 8.15 (d, *J* = 2.5 Hz, 1H), 7.79 (d, *J* = 9.0 Hz, 1H), 7.61 (dd, *J* = 8.9, 2.5 Hz, 1H), 7.46 – 7.34 (m, 5H), 7.14 – 7.07 (m, 2H), 7.04 – 6.99 (m, 2H), 6.06 (dd, *J* = 5.5, 2.1 Hz, 1H), 5.37 (d, *J* = 12.2 Hz, 1H), 5.29 (d, *J* = 12.1 Hz, 1H), 3.11 (dd, *J* = 17.2, 5.5 Hz, 1H), 2.95 (dd, *J* = 17.1, 2.2 Hz, 1H), 2.28 – 2.22 (m, 3H).
^{13}C {^{1}H} NMR (151 MHz, $CDCl_3$) δ 190.7, 152.8, 140.0, 138.0, 137.1, 135.2, 132.4, 129.8, 129.7, 128.9, 128.8, 128.7, 128.4, 128.1, 126.0, 125.9, 121.3, 117.9, 85.4, 84.5, 68.9, 47.2, 44.3, 21.1.
HRMS (APCI+, m/Z): calcd for $C_{18}H_{13}BrNO$ $[M]^+$: 340.0155, found: 340.0145. The HRMS did not successfully afford the exact mass for the title compound. The fragment resulting from the loss of Cbz was found instead.

Benzyl 6-methyl-4-oxo-2-(m-tolylethynyl)-3,4-dihydroquinoline-1(2H)-carboxylate (3hg).

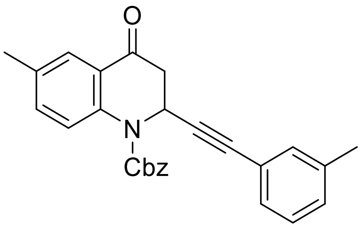

The **general procedure A** was followed, affording 34.6 mg (85%) of the product as a white solid.
^{1}H NMR (400 MHz, $CDCl_3$) δ 7.83 (d, J = 1.6 Hz, 1H), 7.73 (d, J = 8.6 Hz, 1H), 7.45 – 7.31 (m, 6H), 7.12 – 6.98 (m, 4H), 6.05 (dd, J = 5.5, 2.0 Hz, 1H), 5.37 (d, J = 12.3 Hz, 1H), 5.28 (d, J = 12.3 Hz, 1H), 3.11 (dd, J = 17.1, 5.6 Hz, 1H), 2.92 (dd, J = 17.1, 2.1 Hz, 1H), 2.35 (s, 3H), 2.24 (s, 3H).
^{13}C {^{1}H} NMR (101 MHz, $CDCl_3$) δ 192.4, 153.3, 138.8, 138.0, 135.7, 135.5, 134.5, 132.5, 129.7, 129.0, 128.9, 128.7, 128.4, 128.2, 127.2, 124.7, 124.2, 121.8, 85.3, 85.0, 68.7, 47.4, 44.8, 21.2, 20.8.
HRMS (ESI+, m/Z): calcd for $C_{27}H_{23}NO_3H$ [M+H]$^+$: 410.1751, found: 410.1749.

1-Benzyl 6-methyl 4-oxo-2-(m-tolylethynyl)-3,4-dihydroquinoline-1,6(2H)-dicarboxylate (3ig).

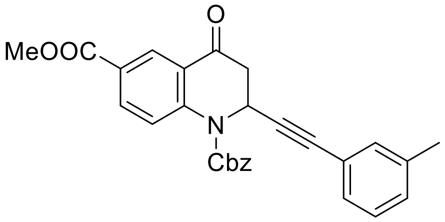

The **general procedure A** was followed, affording 35.4 mg (78%) of the product as a white solid.
^{1}H NMR (400 MHz, $CDCl_3$) δ 8.72 (d, J = 2.2 Hz, 1H), 8.18 (dd, J = 8.8, 2.2 Hz, 1H), 8.01 (d, J = 8.8 Hz, 1H), 7.50 – 7.32 (m, 5H), 7.12 – 6.95 (m, 4H), 6.09 (dd, J = 5.5, 2.2 Hz, 1H), 5.40 (d, J = 12.2 Hz, 1H), 5.32 (d, J = 12.1 Hz, 1H), 3.92 (s, 3H), 3.14 (dd, J = 17.1, 5.5 Hz, 1H), 3.00 (dd, J = 17.1, 2.2 Hz, 1H), 2.23 (s, 3H).
^{13}C {^{1}H} NMR (151 MHz, $CDCl_3$) δ 192.1, 156.4, 153.1, 137.9, 135.6, 134.8, 132.4, 129.5, 128.9, 128.7, 128.5, 128.2, 128.1, 125.9, 125.7, 122.5, 121.6, 108.7, 85.2, 84.9, 68.5, 55.7, 47.3, 44.6, 21.1.
HRMS (ESI+, m/Z): calcd for $C_{28}H_{23}NO_5H$ [M+H]$^+$: 454.1649, found: 454.1627.

Benzyl 8-oxo-6-(m-tolylethynyl)-7,8-dihydro-[1,3]dioxolo[4,5-g]quinoline-5(6H)-carboxylate (3jg).

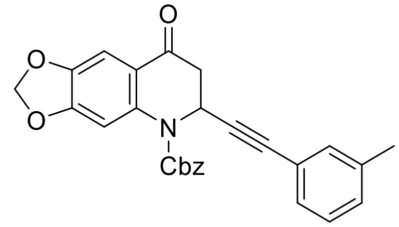

The **general procedure A** was followed, affording 34.8 mg (79%) of the product as a white solid.
^{1}H NMR (400 MHz, $CDCl_3$) δ 7.44 – 7.28 (m, 7H), 7.13 – 7.01 (m, 4H), 6.04 – 6.00 (m, 3H), 5.36 (d, J = 12.2 Hz, 1H), 5.27 (d, J = 12.2 Hz, 1H), 3.05 (dd, J = 17.1, 5.6 Hz, 1H), 2.87 (dd, J = 17.2, 2.0 Hz, 1H), 2.25 (s, 3H).
^{13}C {^{1}H} NMR (101 MHz, $CDCl_3$) δ 193.0, 155.6, 155.4, 147.7, 140.8, 140.6, 138.1, 135.1, 132.2, 131.6, 131.4, 131.2, 130.9, 130.7, 124.3, 122.5, 107.8, 107.7, 104.8, 87.7, 87.3, 71.3, 50.3, 46.7, 23.8.
HRMS (ESI+, m/Z): calcd for $C_{27}H_{21}NO_5H$ [M+H]$^+$: 440.1493, found: 440.1480.

Benzyl 5,7-dimethyl-4-oxo-2-(m-tolylethynyl)-3,4-dihydroquinoline-1(2H)-carboxylate (3kg).

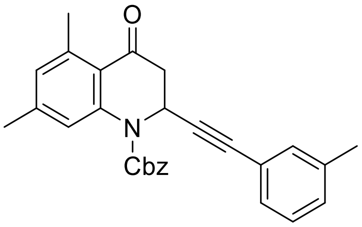

The **general procedure A** was followed, affording 35.2 mg (83%) of the product as a white solid.

^{1}H NMR (400 MHz, $CDCl_3$) δ 7.44 – 7.31 (m, 6H), 7.12 – 7.04 (m, 2H), 7.02 – 6.96 (m, 2H), 6.85 (s, 1H), 5.98 (dd, *J* = 5.9, 1.8 Hz, 1H), 5.38 (d, *J* = 12.3 Hz, 1H), 5.22 (d, *J* = 12.3 Hz, 1H), 3.10 (dd, *J* = 17.4, 6.0 Hz, 1H), 2.88 (dd, *J* = 17.4, 1.8 Hz, 1H), 2.63 (s, 3H), 2.31 (s, 3H), 2.24 (s, 3H).

^{13}C {^{1}H} NMR (101 MHz, $CDCl_3$) δ 193.4, 153.2, 143.7, 141.9, 141.5, 137.9, 135.6, 132.3, 130.1, 129.4, 128.8, 128.7, 128.4, 128.1, 128.0, 123.7, 121.9, 121.8, 85.5, 84.6, 68.4, 46.8, 46.1, 23.2, 21.8, 21.1.

HRMS (ESI+, m/Z): calcd for $C_{28}H_{25}NO_3H$ [M+H]$^+$: 424.1907, found: 424.1906.

(*R*)–Benzyl 2-((3,4-dimethoxyphenyl)ethynyl)-4-oxo-3,4-dihydroquinoline-1(2H)-carboxylate (3ap)

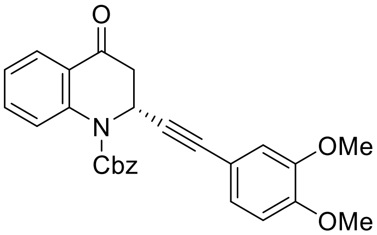

The **general procedure A** was followed, affording 41.2 mg (93%) of the product as a yellow solid.

The **general procedure B** was followed, affording 39.2 mg (89%) of the product as a yellow solid. 34% ee.

^{1}H NMR (400 MHz, $CDCl_3$) δ 8.05 (dd, *J* = 7.8, 1.7 Hz, 1H), 7.85 (d, *J* = 8.4 Hz, 1H), 7.57 – 7.50 (m, 1H), 7.46 – 7.33 (m, 5H), 7.21 (m, 1H), 6.79 (dd, *J* = 8.3, 1.8 Hz, 1H), 6.70 – 6.64 (m, 2H), 6.07 (dd, *J* = 5.5, 2.1 Hz, 1H), 5.39 (d, *J* = 12.2 Hz, 1H), 5.29 (d, *J* = 12.2 Hz, 1H), 3.82 (s, 3H), 3.78 (s, 3H), 3.14 (dd, *J* = 17.1, 5.5 Hz, 1H), 2.95 (dd, *J* = 17.1, 2.1 Hz, 1H).

^{13}C {^{1}H} NMR (101 MHz, $CDCl_3$) δ 192.2, 153.2, 149.9, 148.6, 141.3, 135.6, 134.6, 128.9, 128.7, 128.4, 127.2, 125.4, 125.0, 124.7, 124.3, 114.5, 114.0, 110.9, 85.0, 84.1, 68.8, 56.0, 56.0, 47.5, 44.8.

HRMS (APCI+, m/Z): calcd for $C_{19}H_{16}NO_3$ [M]$^+$: 306.1125, found: 306.1121. The HRMS did not successfully afford the exact mass for the title compound. The fragment resulting from the loss of Cbz was found instead.

SFC analysis: Chiralpak AS, 3um, 3.0*150mm, 98/2 CO2/MeOH for 2 min, then, 98/2 CO2/MeOH to 50/50 CO2/MeOH gradient from 2.0-10.0 min, 1.8 mL/min, 40 ^{o}C, detection at 254 nm. Retention time (min): 4.98 (minor) and 5.28 (major).

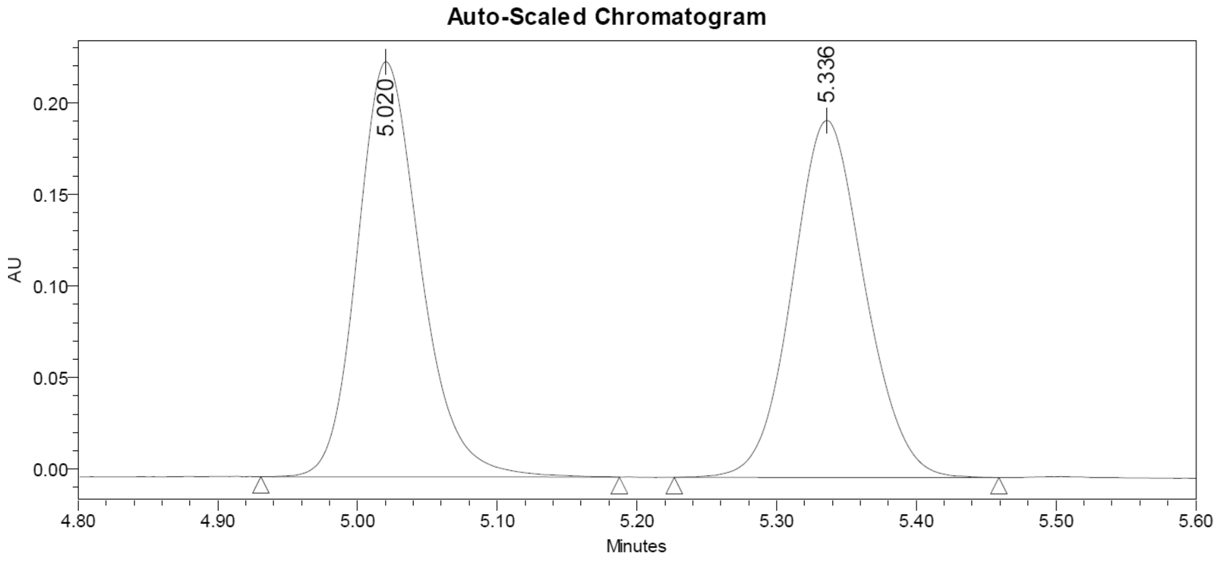

Peak Results

	Name	RT	Area	% Area
1		5.020	713478	50.39
2		5.336	702513	49.61

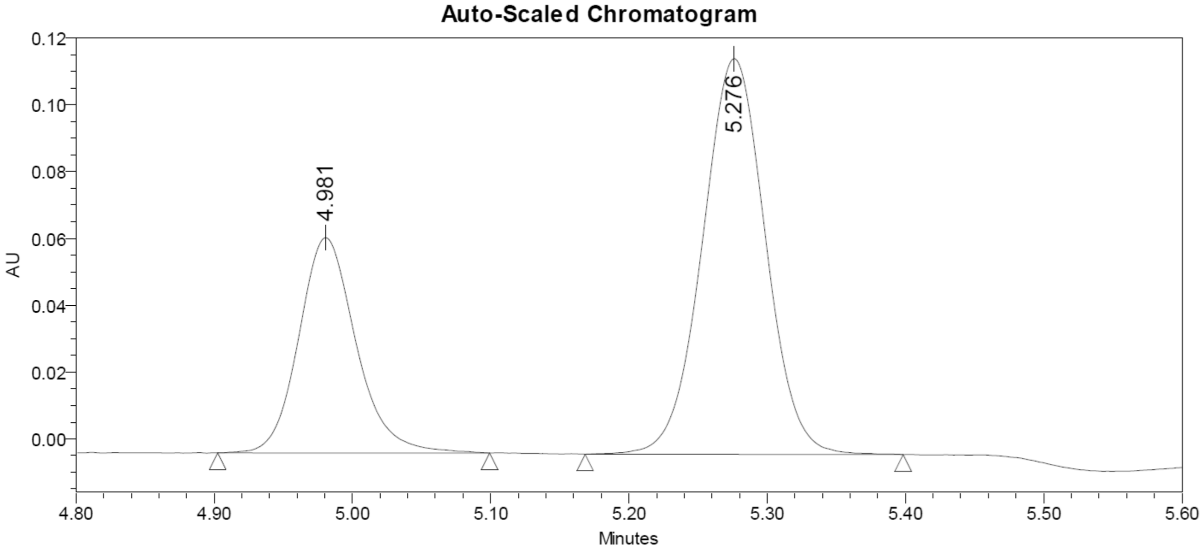

Peak Results

	Name	RT	Area	% Area
1		4.981	186040	33.09
2		5.276	376179	66.91

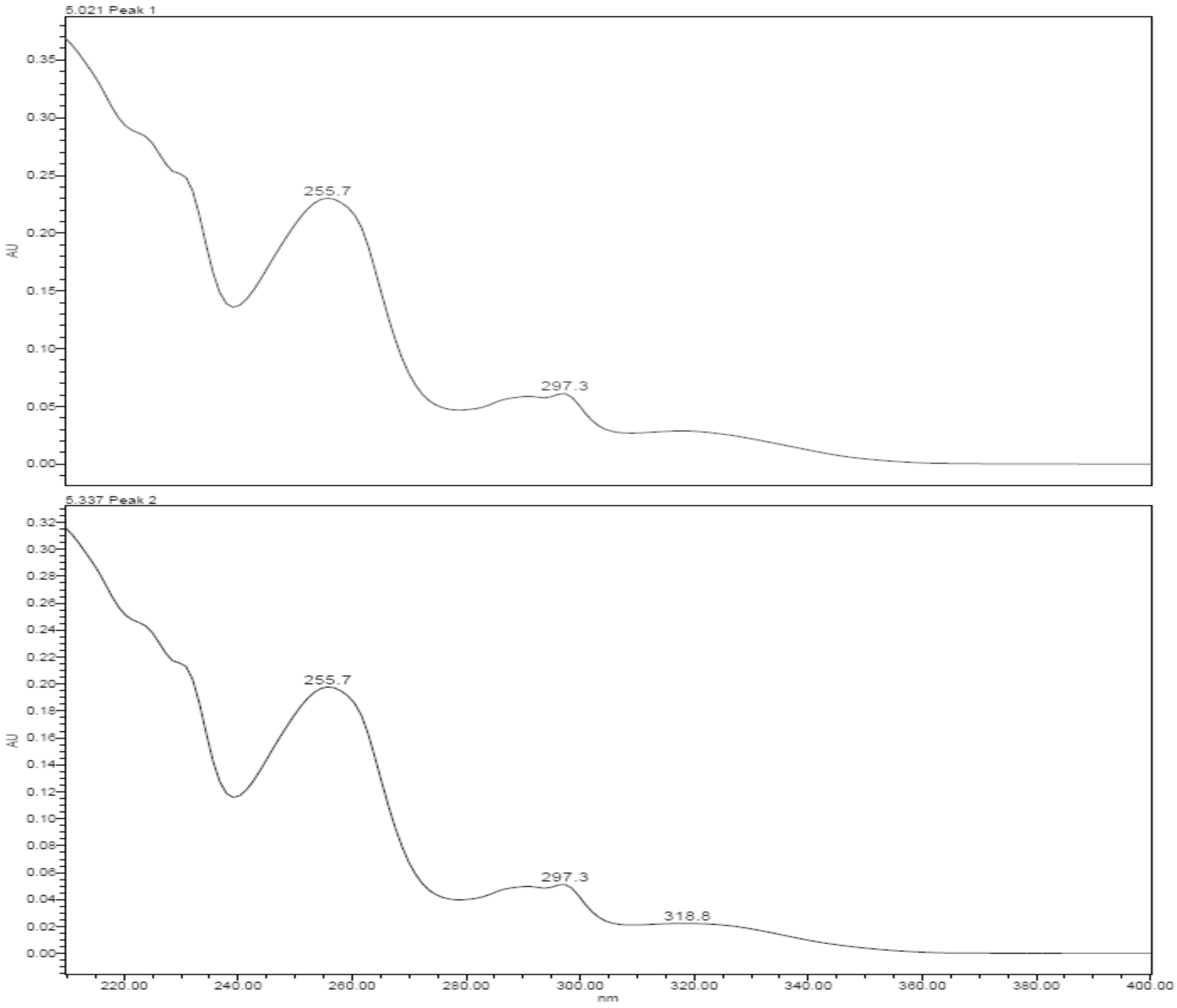
5.021 Peak 1
255.7
297.3
AU
5.337 Peak 2
255.7
297.3
318.8
AU
nm

Synthesis and characterization of compounds **4** and **5**.

Synthesis of (*R*)–2-(phenylethynyl)-2,3-dihydroquinolin-4(1H)-one (4)

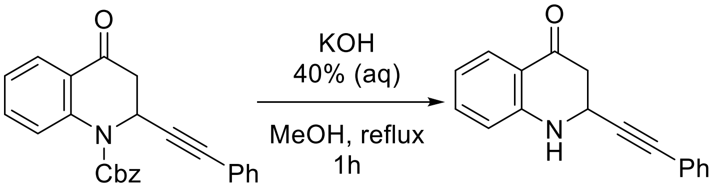

3aa 351 mg (0.92 mmol) → **4** 205 mg 90% (0.83 mmol)

Following a previously reported procedure,[2] KOH (5 mL; 40% aqueous) was added to a solution of **3aa** (351 mg, 0.92 mmol) in MeOH (5 mL) and the resulting mixture was refluxed for 1h (oil bath), turning into an orange solution. After cooling to room temperature, the reaction mixture was extracted with Et_2O and the organic layer was washed with brine, dried over $MgSO_4$, filtered, and concentrated under vacuum. The residue was purified by chromatography on silica gel (pentane/Et_2O 8:2) to afford 205 mg of **4** as a white solid (90% yield).

^{1}H NMR (400 MHz, $CDCl_3$) δ 7.88 (dd, *J* = 8.0, 1.6 Hz, 1H), 7.40 – 7.28 (m, 5H), 6.82 (ddd, *J* = 8.0, 7.1, 1.0 Hz, 1H), 6.74 (d, *J* = 8.2 Hz, 1H), 4.73 (dd, *J* = 8.5, 5.1 Hz, 1H), 4.61 (bs, 1H), 3.04 – 2.88 (m, 2H).

^{13}C {^{1}H} NMR (101 MHz, $CDCl_3$) δ 192.2, 150.2, 135.5, 132.0, 128.8, 128.5, 127.7, 122.2, 119.7, 119.2, 116.4, 87.2, 84.3, 45.7, 44.1.

HRMS (APCI+, m/Z): calcd for $C_{17}H_{12}NO$ $[M-H]^+$: 246.0913, found: 246.0910.

Synthesis of (+)-Cuspareine (5)

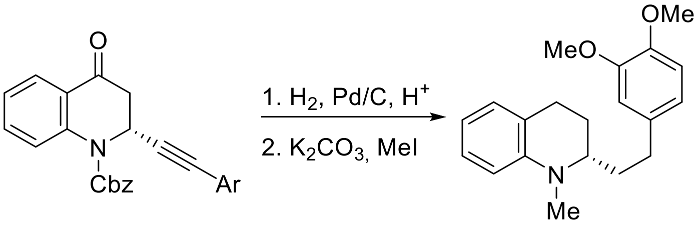

3ap 34% ee → **5** (+)-Cuspareine 82% (from **3ap**) 36% ee

Step 1: Following a previously reported procedure,[2] in a flame-dried Schlenk tube Pd/C 10% (106.4 mg, 0.1 mmol, 0.5 equiv.) was added to a flask under a hydrogen atmosphere (hydrogen balloon). Then, a solution of **3ap** (88.3 mg, 0.2 mmol, 34% ee, 1.0 equiv.) in MeOH (5 mL) was injected into the flask and TFA (0.2 mL) was added to the solution. The reaction was stirred at room temperature for 12 h. After that, the Pd/C was filtered through celite and the filtrate evaporated to remove the TFA and solvent. The obtained colourless liquid was used for the next step without further purification.

Step 2: To a solution of the above crude product and K_2CO_3 (82.9 mg, 0.6 mmol, 3.0 equiv.) in THF (5 mL), MeI (0.062 mL, 1.0 mmol, 5.0 equiv.) was added under a nitrogen atmosphere. After refluxing for 12 h (oil bath), the reaction mixture was quenched with water and extracted with CH_2Cl_2 (3×10 mL). The combined organic layers were washed with brine and dried over anhydrous $MgSO_4$. After removal of the solvent, the residue was purified by flash column chromatography on silica gel (Pentane: Et_2O = 95:5) to give 51.2 mg of (+)-Cuspareine **5** as a colourless oil (82%, 36% ee).

^{1}H NMR (400 MHz, $CDCl_3$) δ 7.08 (t, *J* = 8.1 Hz, 1H), 6.98 (d, *J* = 7.2 Hz, 1H), 6.79 (d, *J* = 7.9 Hz, 1H), 6.75 – 6.69 (m, 2H), 6.59 (t, *J* = 7.5 Hz, 1H), 6.53 (d, *J* = 8.2 Hz, 1H), 3.86 (s, 3H), 3.85 (s, 3H), 3.35 – 3.22 (m, 1H), 2.91 (s, 3H), 2.90 – 2.78 (m, 1H), 2.73 – 2.61 (m, 2H), 2.59 – 2.47 (m, 1H), 1.98 – 1.85 (m, 3H), 1.80 – 1.66 (m, 1H).

^{13}C {^{1}H} NMR (101 MHz, $CDCl_3$) δ 149.0, 147.3, 145.4, 134.88, 128.8, 127.36, 121.9, 120.2, 115.5, 111.7, 111.4, 110.7, 58.5, 56.1, 56.0, 38.2, 33.2, 32.1, 24.5, 23.7.

HRMS (ESI+, m/Z): calcd for $C_{20}H_{25}NO_2$ $[M+H]^+$: 312.1958, found: 312.1967.

HPLC analysis: Chiralcel-AD-H, n-heptane/i-PrOH 90:10, 0.5 mL/min, 40 °C, detection at 254 nm. Retention time (min): 12.13 (minor) and 15.95 (major).
The NMR data are in accordance with previously reported data.[3]
Lit. $[\alpha]_D^{20}$ = +58.5° (c = 0.85, $CHCl_3$), (*S*-configuration) [4]
Exp. $[\alpha]_D^{20}$ (36% ee) = -4.7° (c = 0.95, $CHCl_3$). (*R*-configuration). The absolute configurations of other compounds were assigned by analogy.

<Chromatogram>

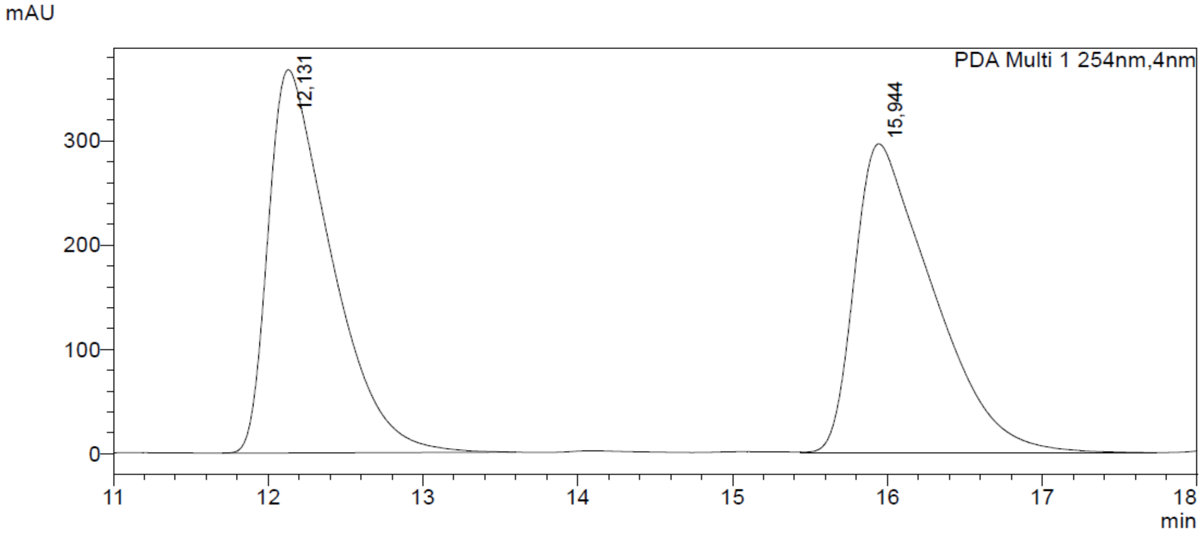

<Peak Table>

PDA Ch1 254nm

Peak#	Ret. Time	Area	Area%
1	12,131	10410237	49,792
2	15,944	10497255	50,208
Total		20907492	100,000

[3] Davies, S. G.; Fletcher, A. M.; Houlsby, I. T. T.; Roberts, P. M.; Thomson, J. E.; Zimmer, D. The Hancock Alkaloids (-)-Cuspareine, (-)-Galipinine, (-)-Galipeine, and (-)-Angustureine: Asymmetric Syntheses and Corrected 1 H and 13 C NMR Data. *J. Nat. Prod.* **2018**, *81*, 2731–2742.
[4] Berthold, D.; Breit, B. Asymmetric Total Syntheses of (-)-Angustureine and (-)-Cuspareine via Rhodium-Catalyzed Hydroamination. *Org. Lett.* **2020**, *22*, 565–568.

<Chromatogram>

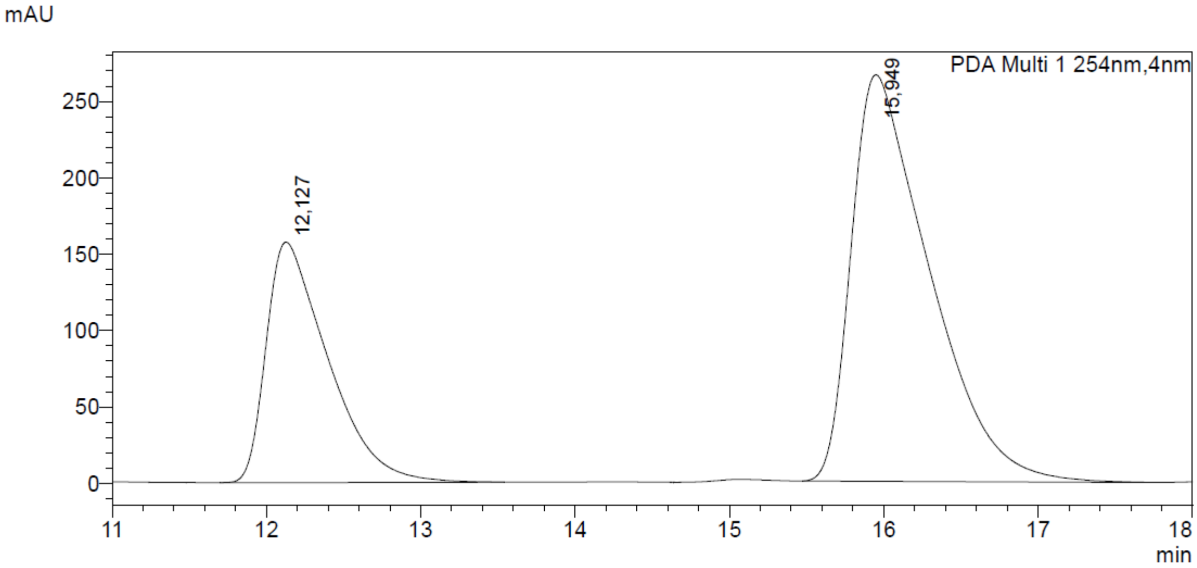

<Peak Table>

PDA Ch1 254nm

Peak#	Ret. Time	Area	Area%
1	12,127	4374093	31,827
2	15,949	9369415	68,173
Total		13743508	100,000

Peak# : 1
Retention Time : 12,127 min
Compound Name :
Spectrum Operation:

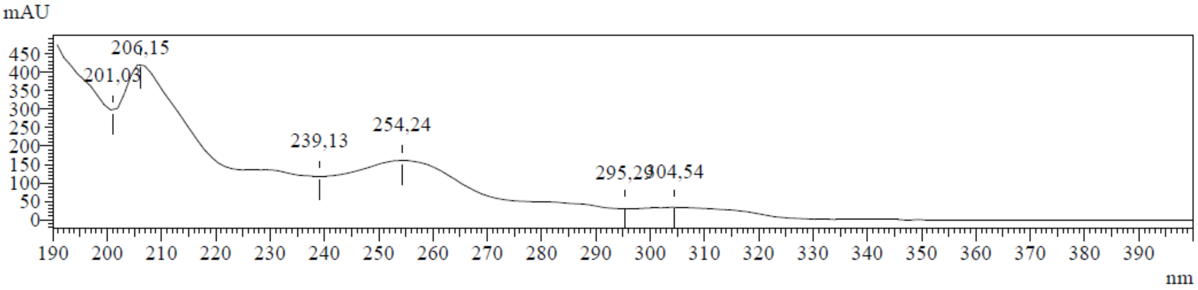

Peak# : 2
Retention Time : 15,949 min
Compound Name :
Spectrum Operation:

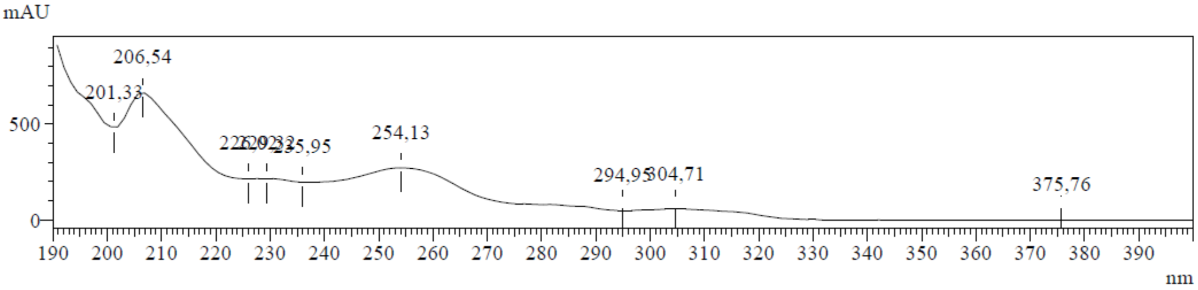

NMR spectra of quinolones **1**

1-Benzyloxycarbonyl-4-quinolone (1a) ($CDCl_3$, ^{1}H 400 MHz, ^{13}C 100 MHz)

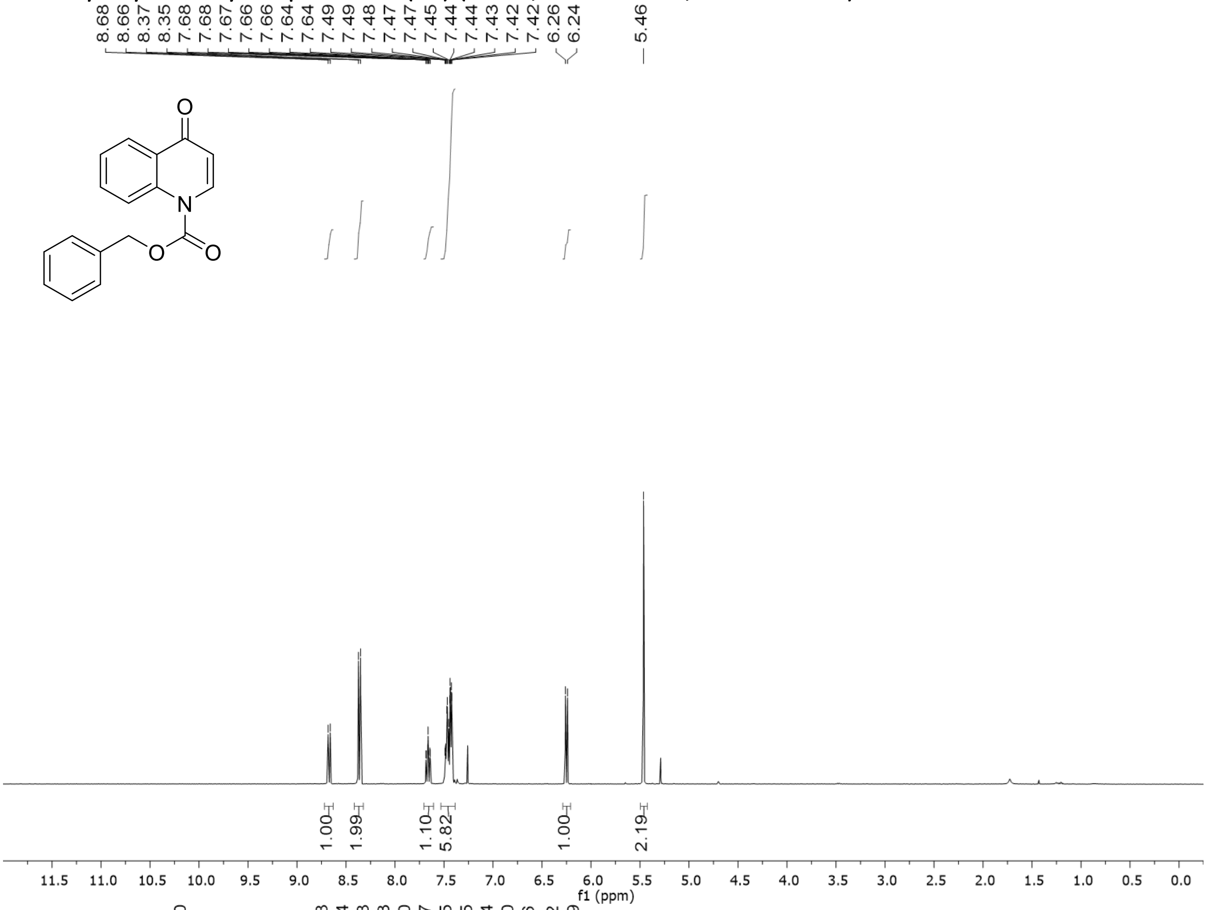

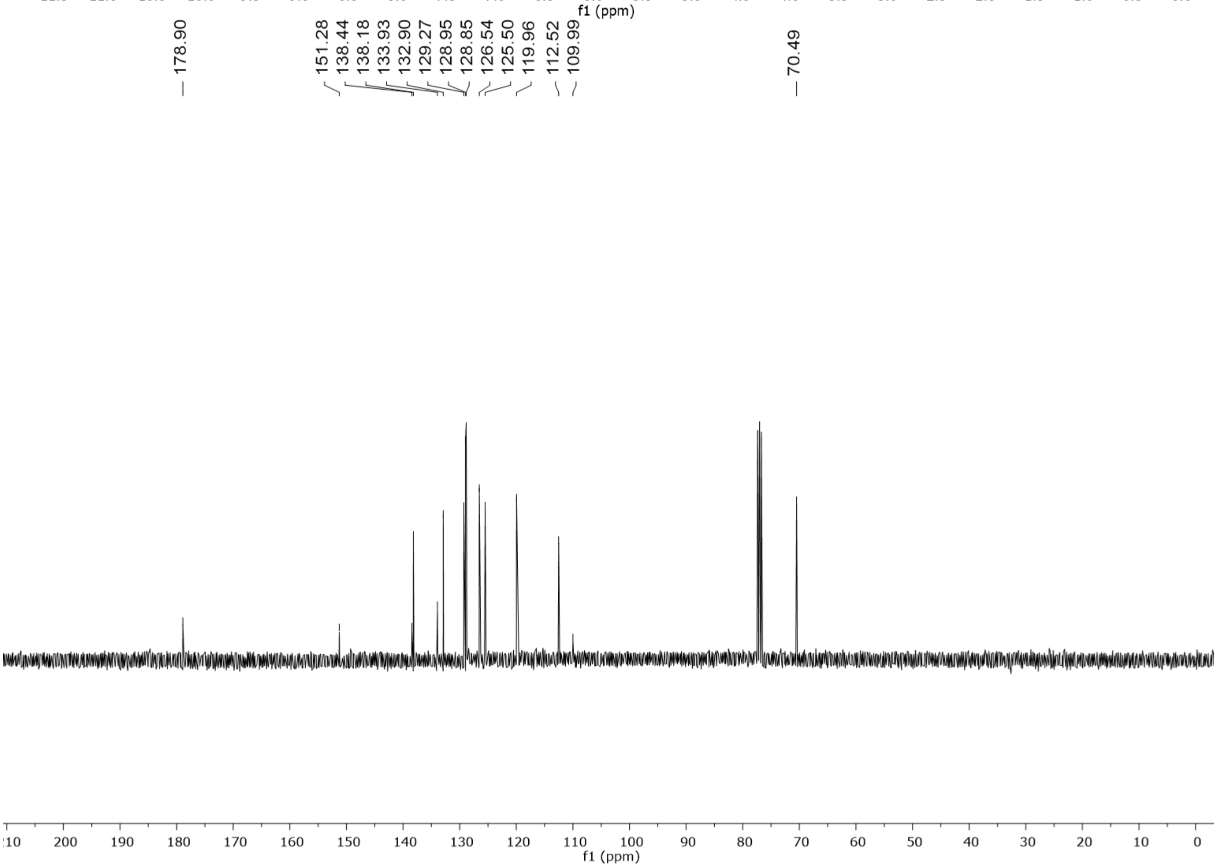

1-Benzyl-4-quinolone (1c) ($CDCl_3$, ^{1}H 400 MHz, ^{13}C 100 MHz)

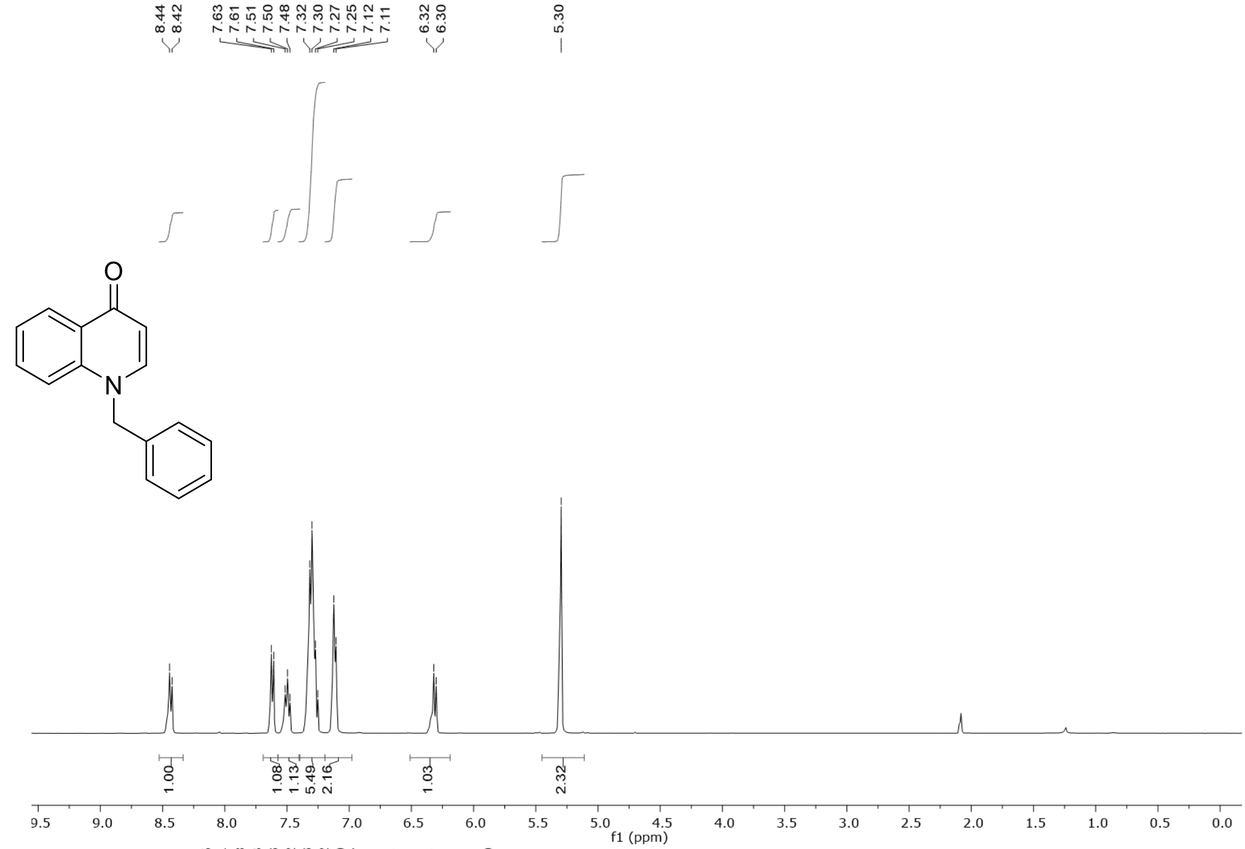

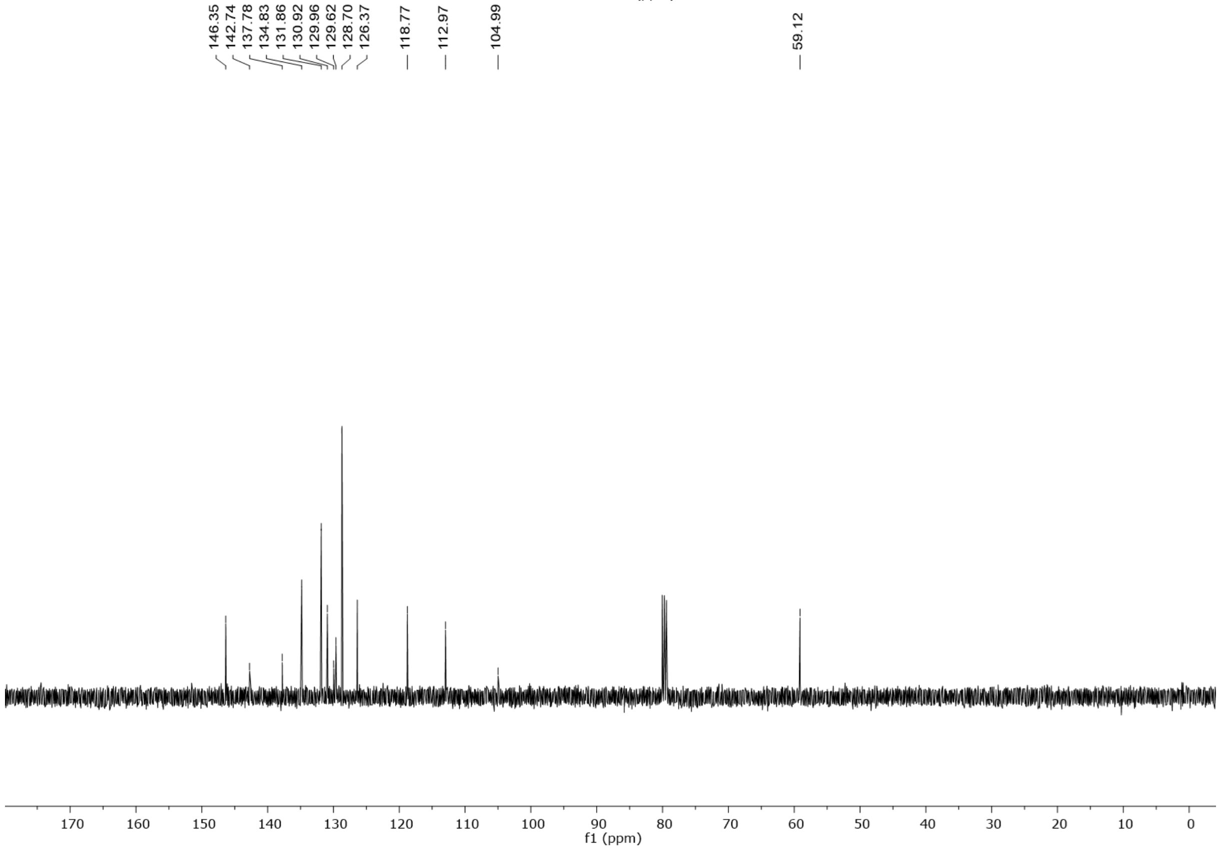

1-Boc-4-quinolone (1d) ($CDCl_3$, ^{1}H 400 MHz, ^{13}C 100 MHz)

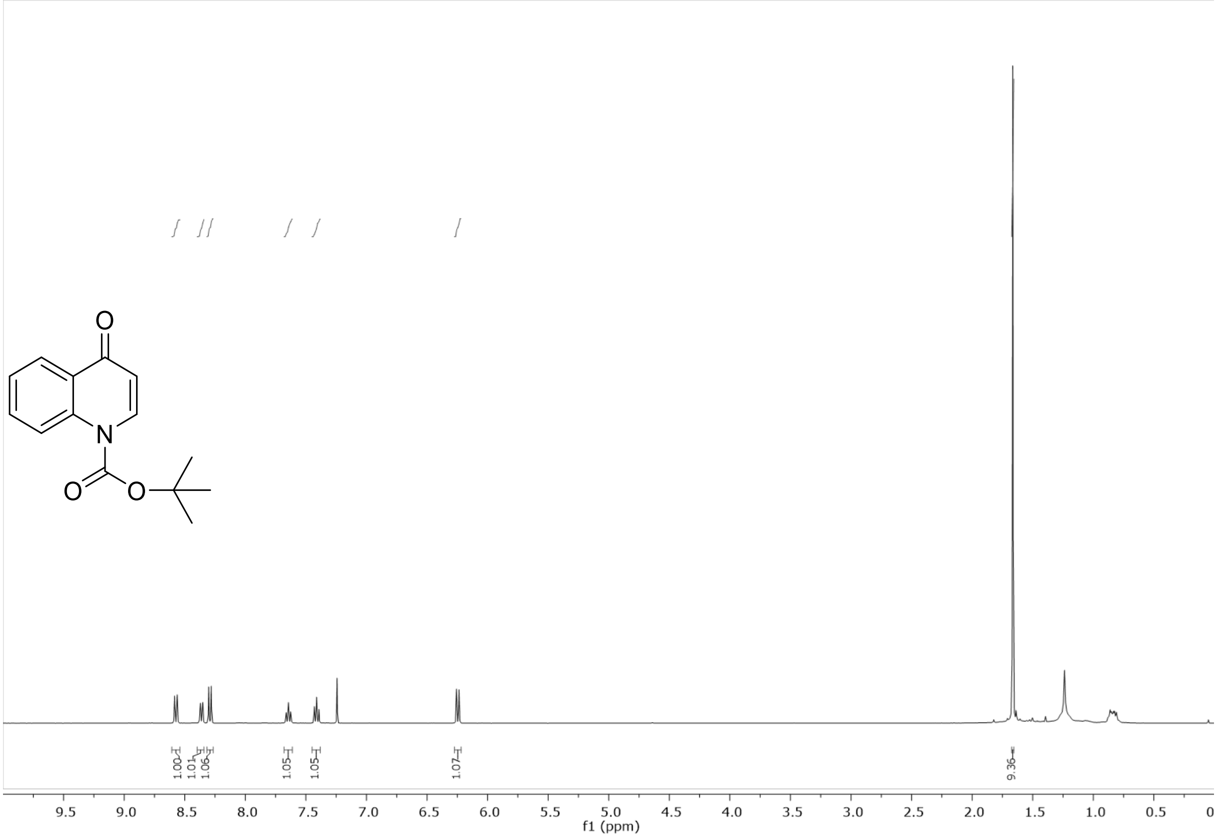

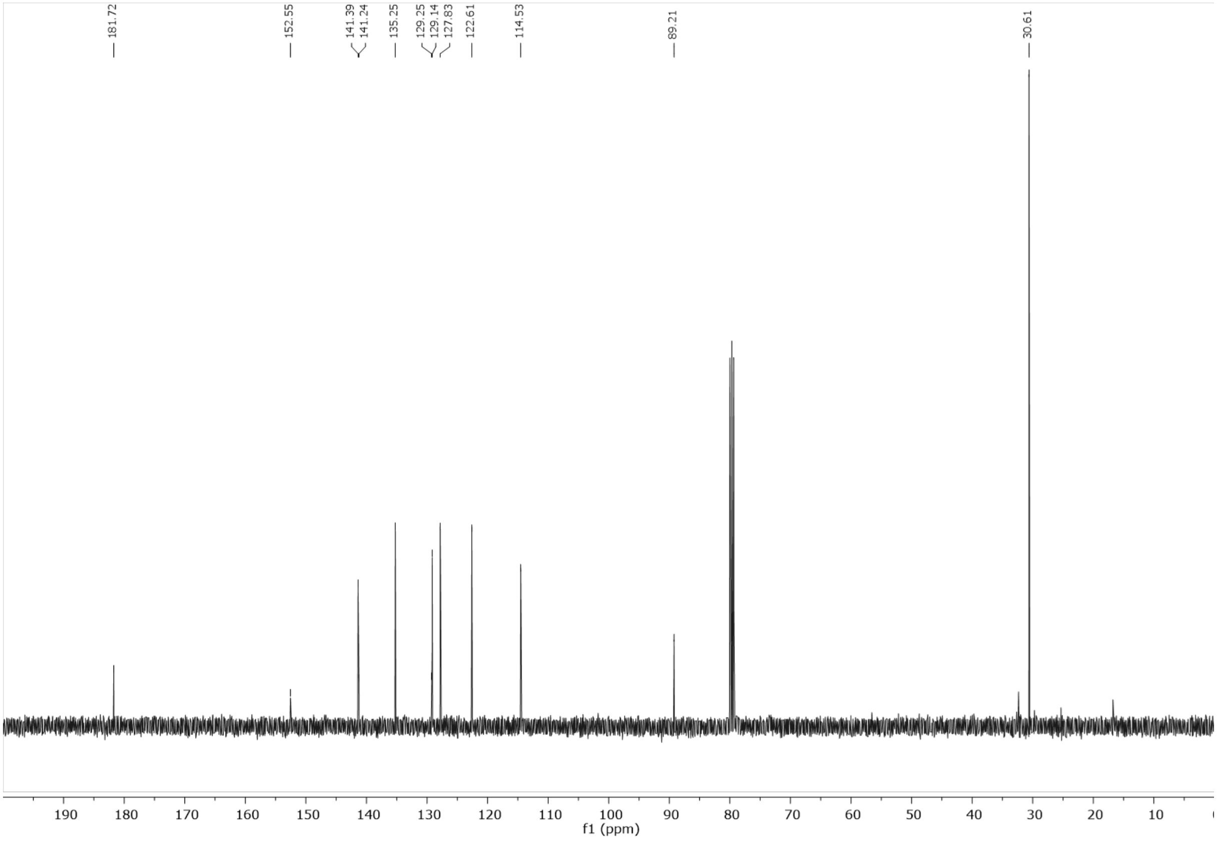

1-Benzyloxycarbonyl-6-Methoxy-4-quinolone (1e) ($CDCl_3$, ^1H 400 MHz, ^{13}C 100 MHz)

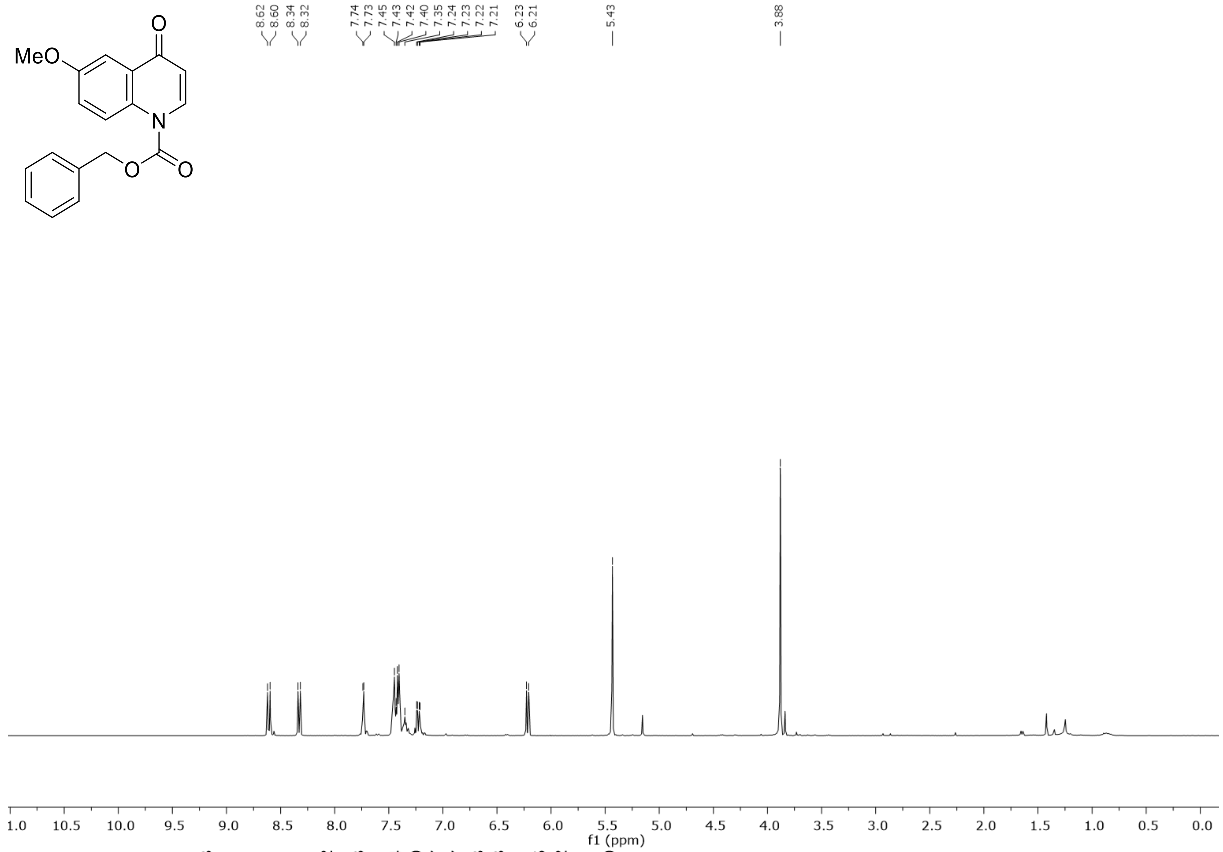

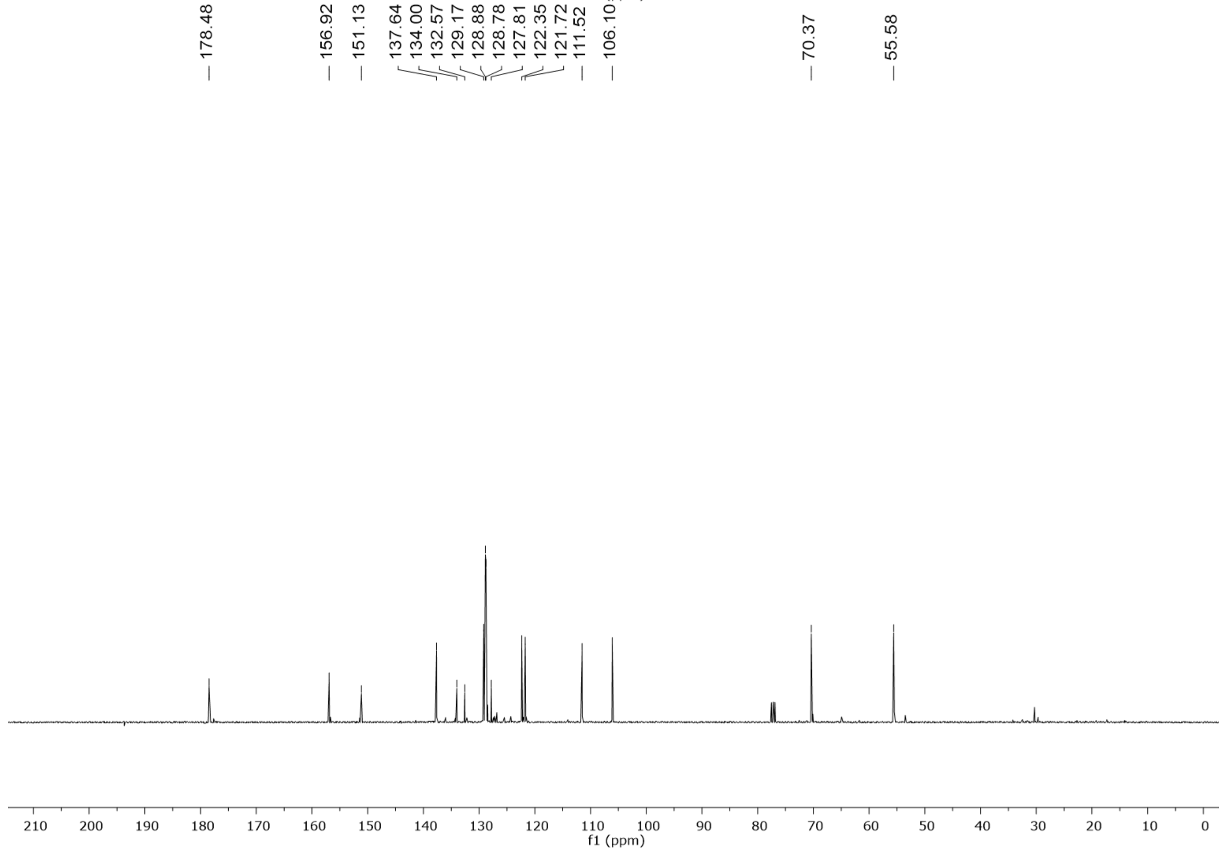

1-Benzyloxycarbonyl-6-Trifloromethyl-4-quinolone (1f) ($CDCl_3$, ^{1}H 400 MHz, ^{13}C 100 MHz, ^{19}F 376 MHz)

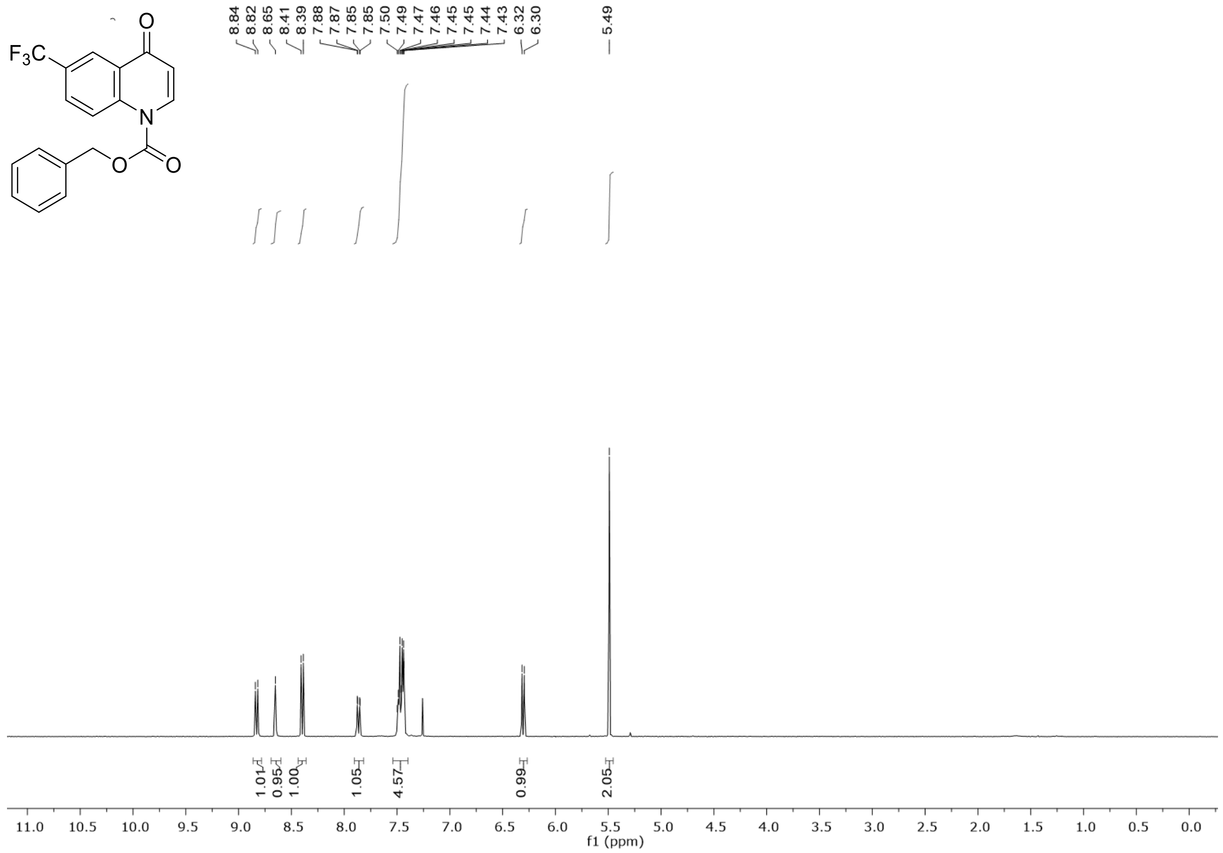

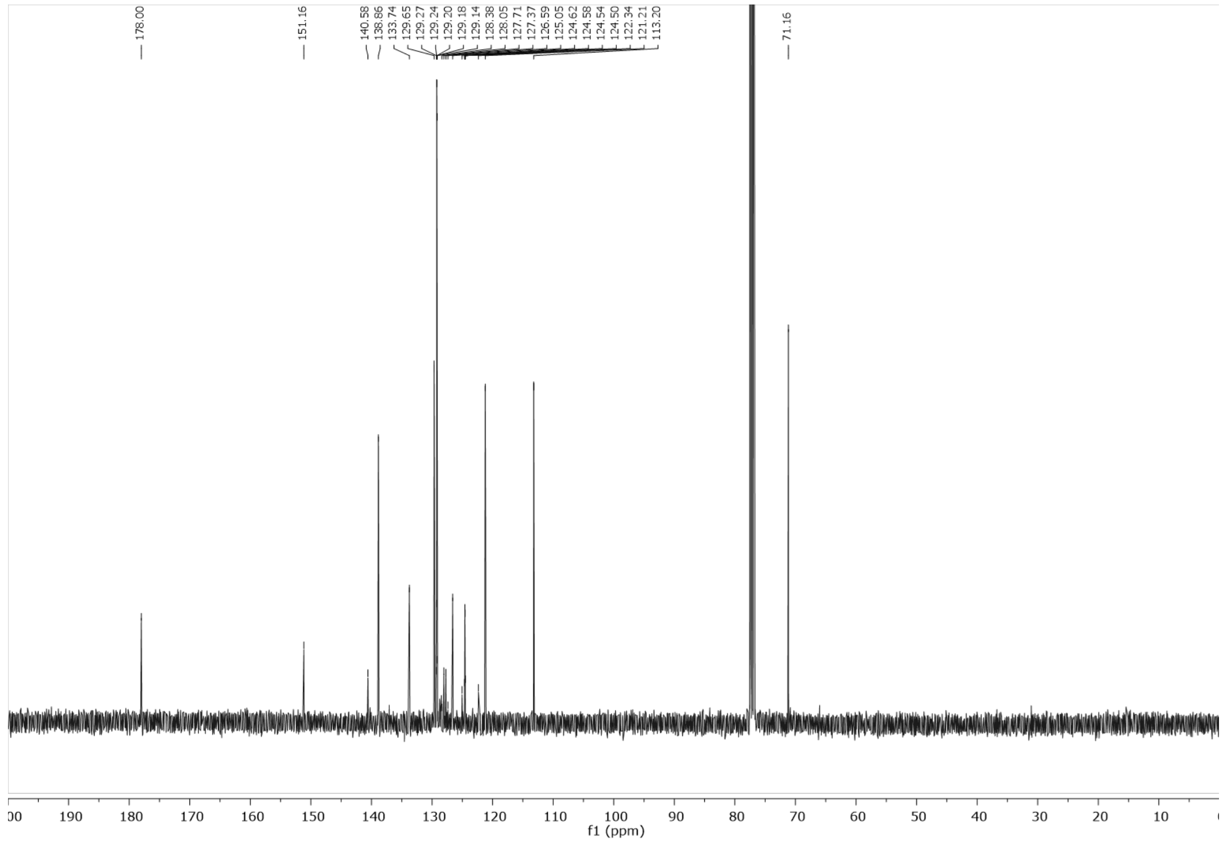

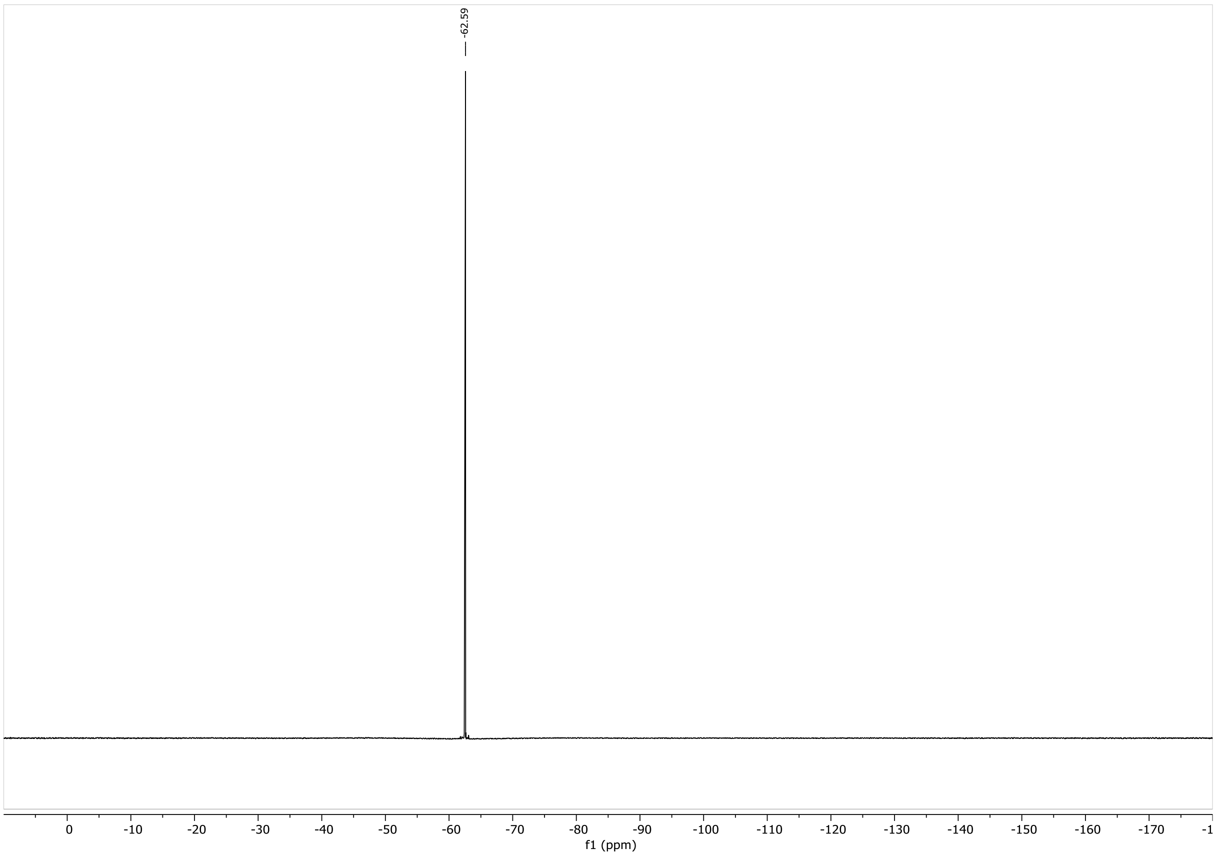
-62.59
0
-10
-20
-30
-40
-50
-60
-70
-80
-90
-100
-110
-120
-130
-140
-150
-160
-170
f1 (ppm)

1-Benzyloxycarbonyl-6-bromo-4-quinolone (1g) ($CDCl_3$, ^1H 400 MHz, ^{13}C 100 MHz)

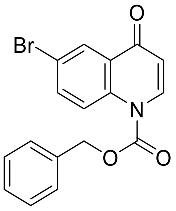

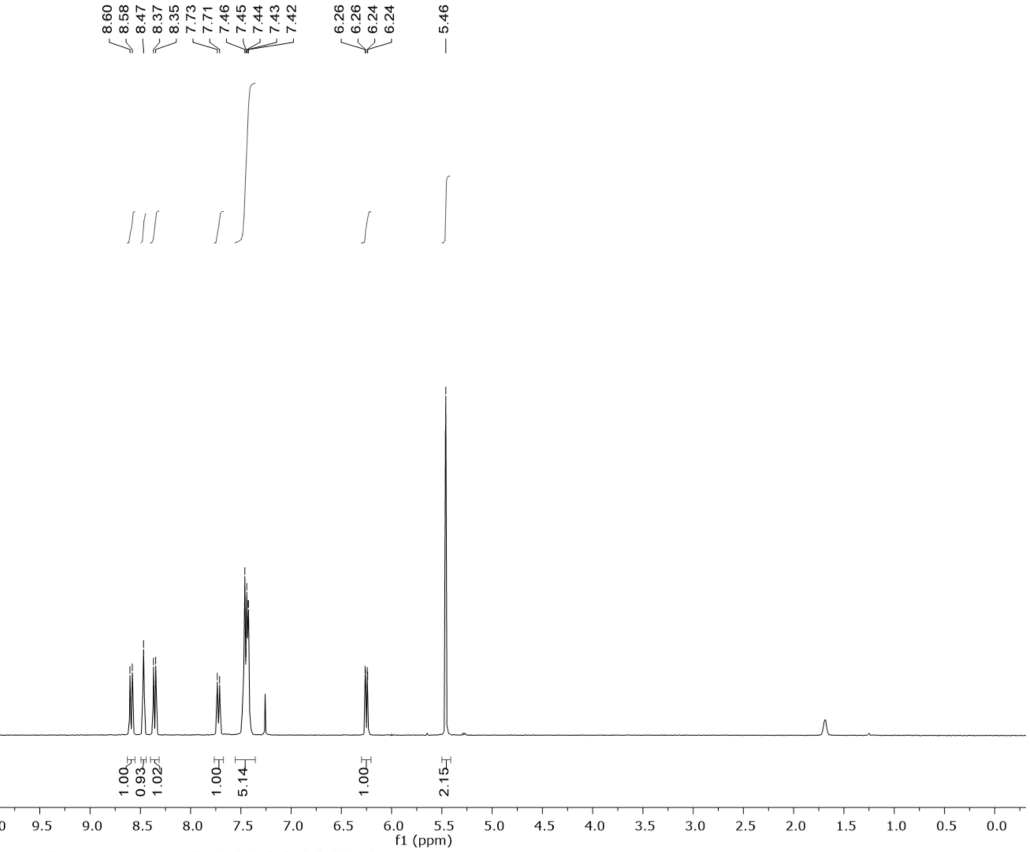

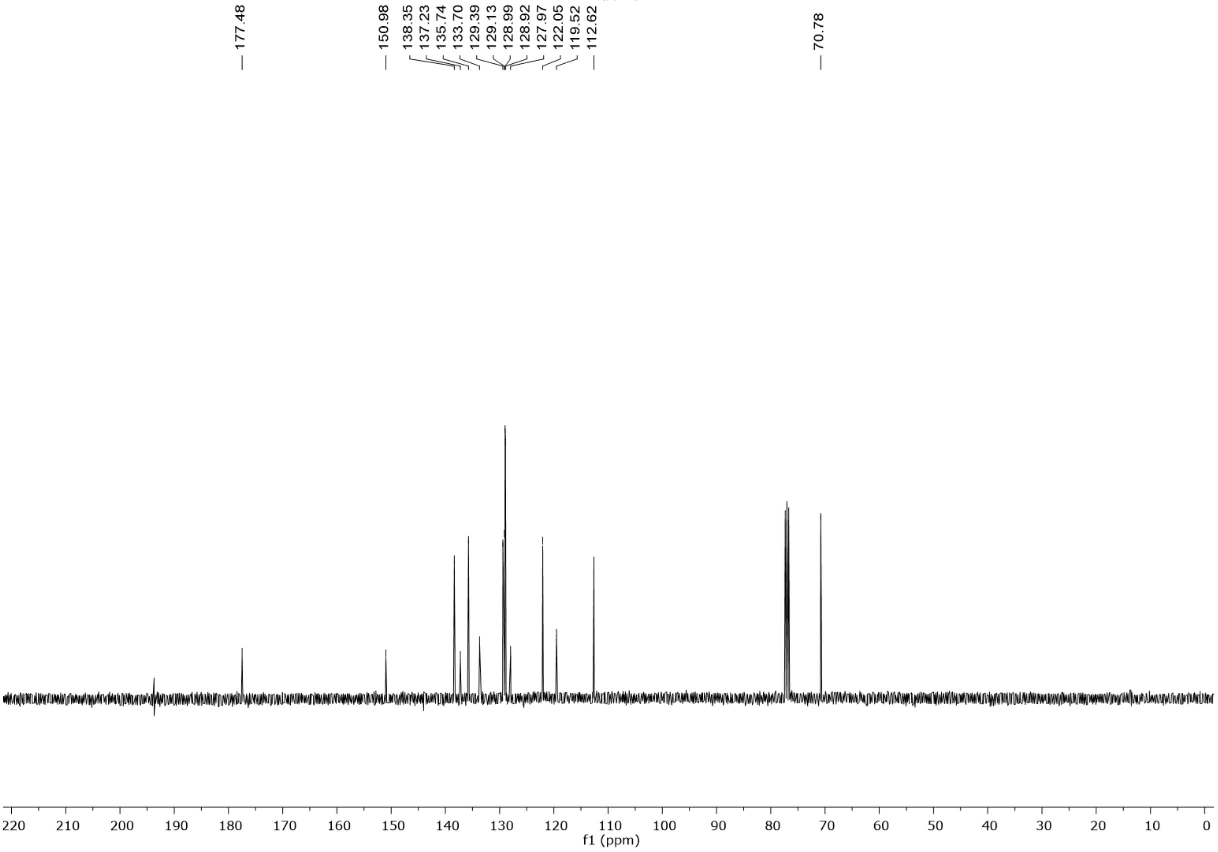

1-Benzyloxycarbonyl-6-methyl-4-quinolone (1h) ($CDCl_3$, ^{1}H 400 MHz, ^{13}C 100 MHz)

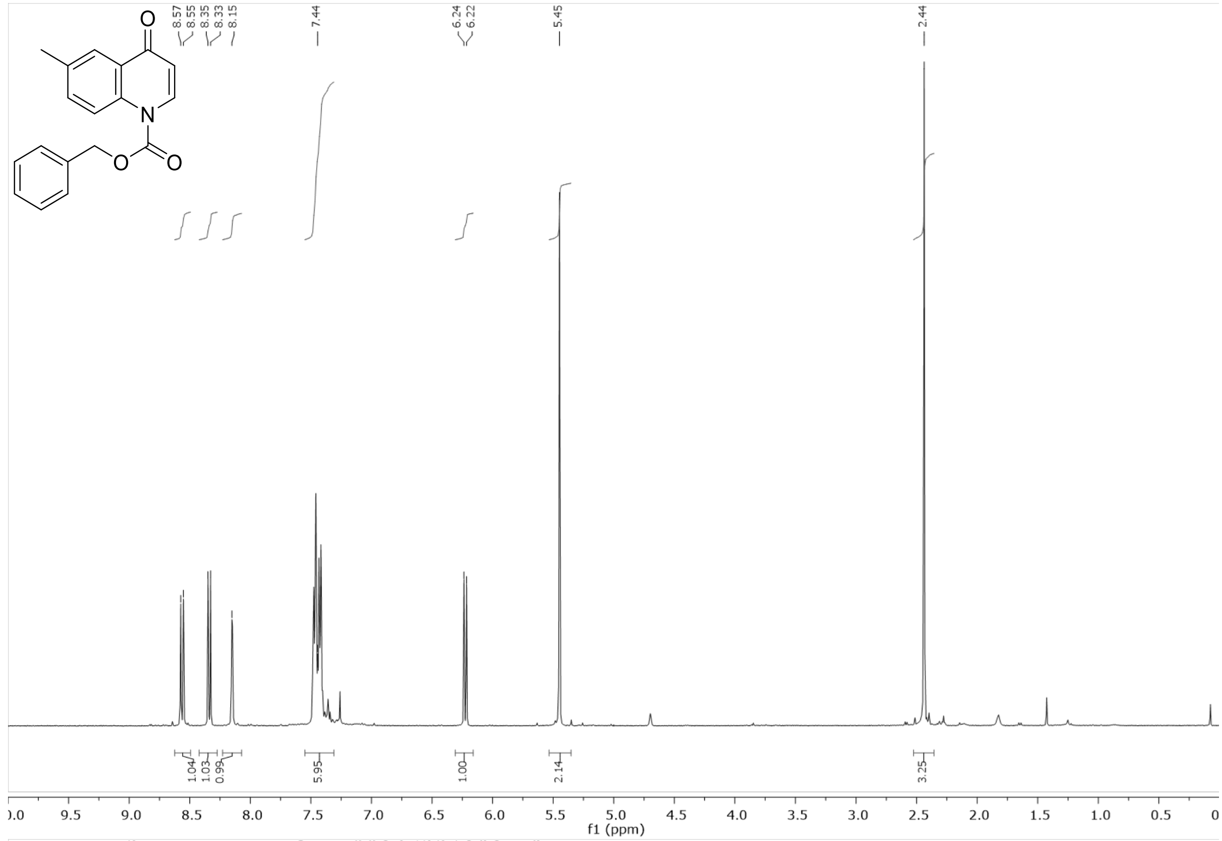

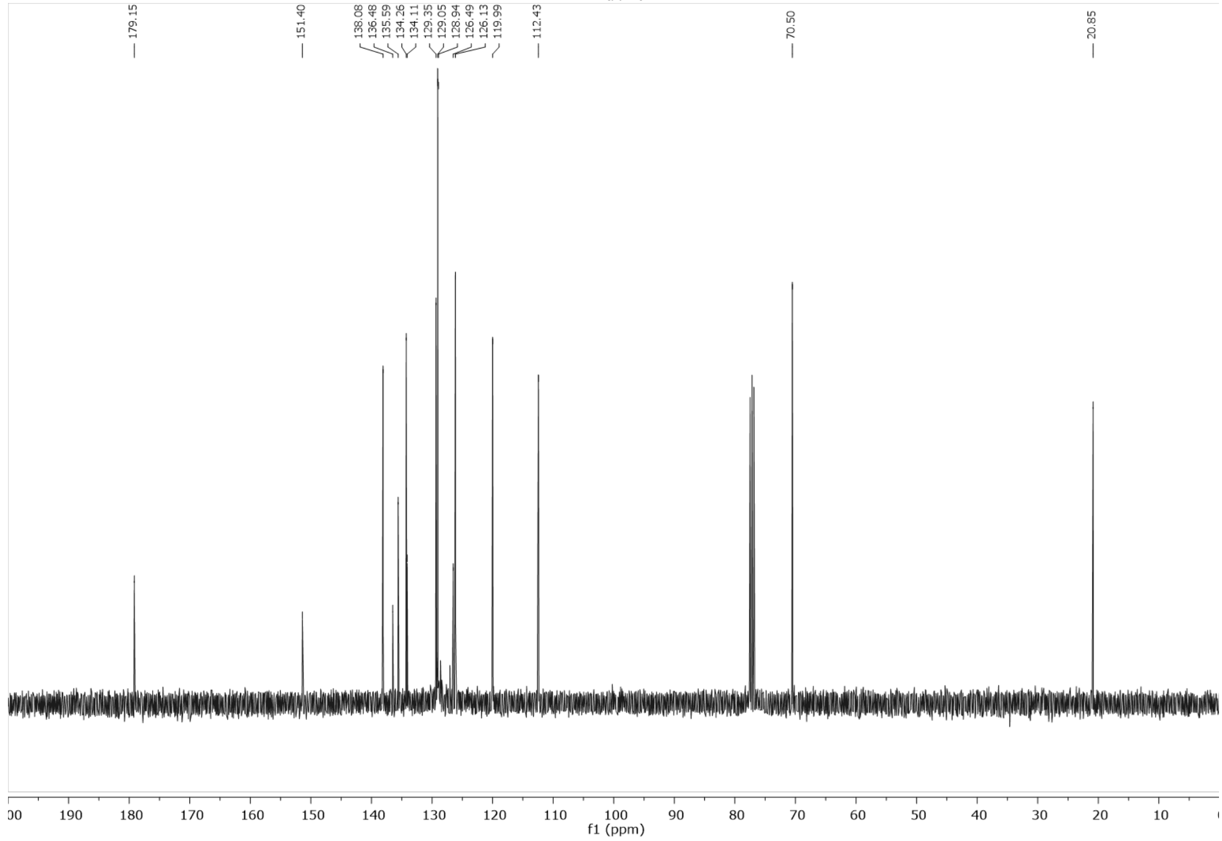

1-Benzyloxycarbonyl-6-methoxycarbonyl-4-quinolone (1i) ($CDCl_3$, ^1H 400 MHz, ^{13}C 100 MHz)

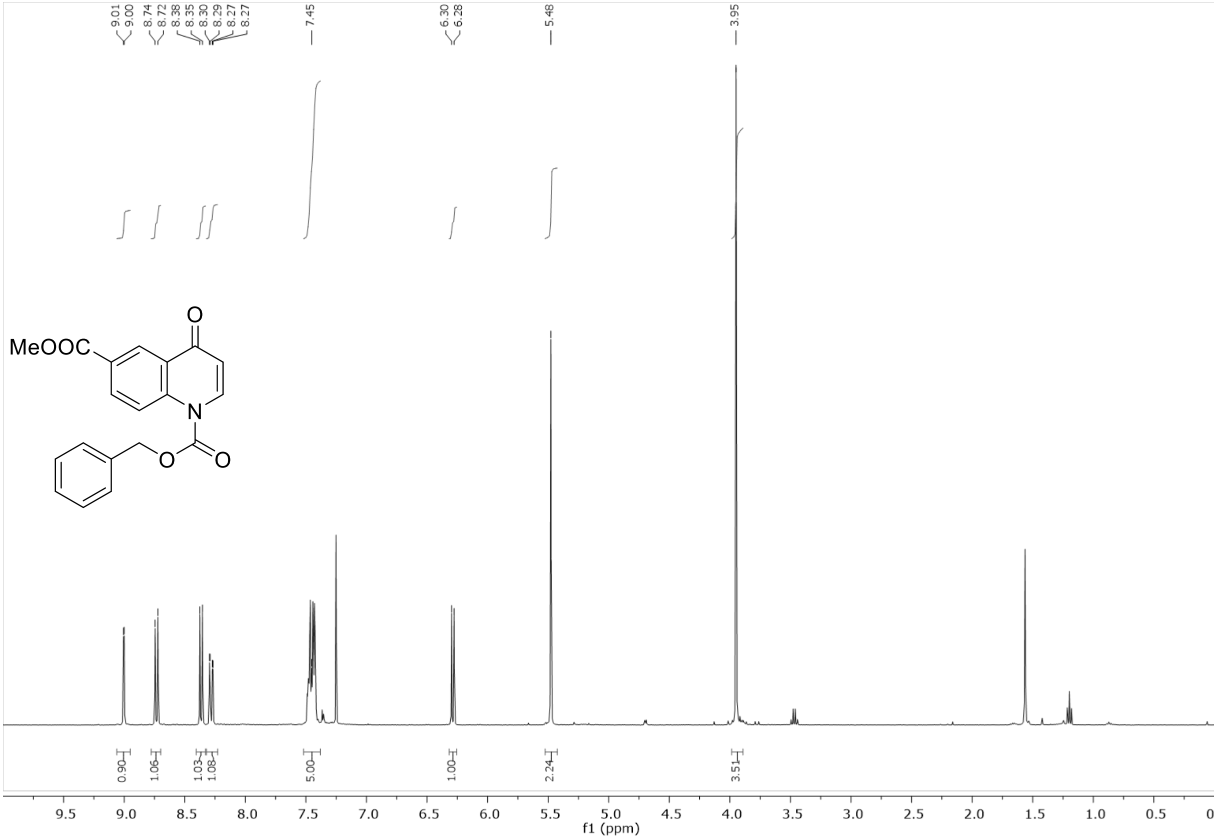

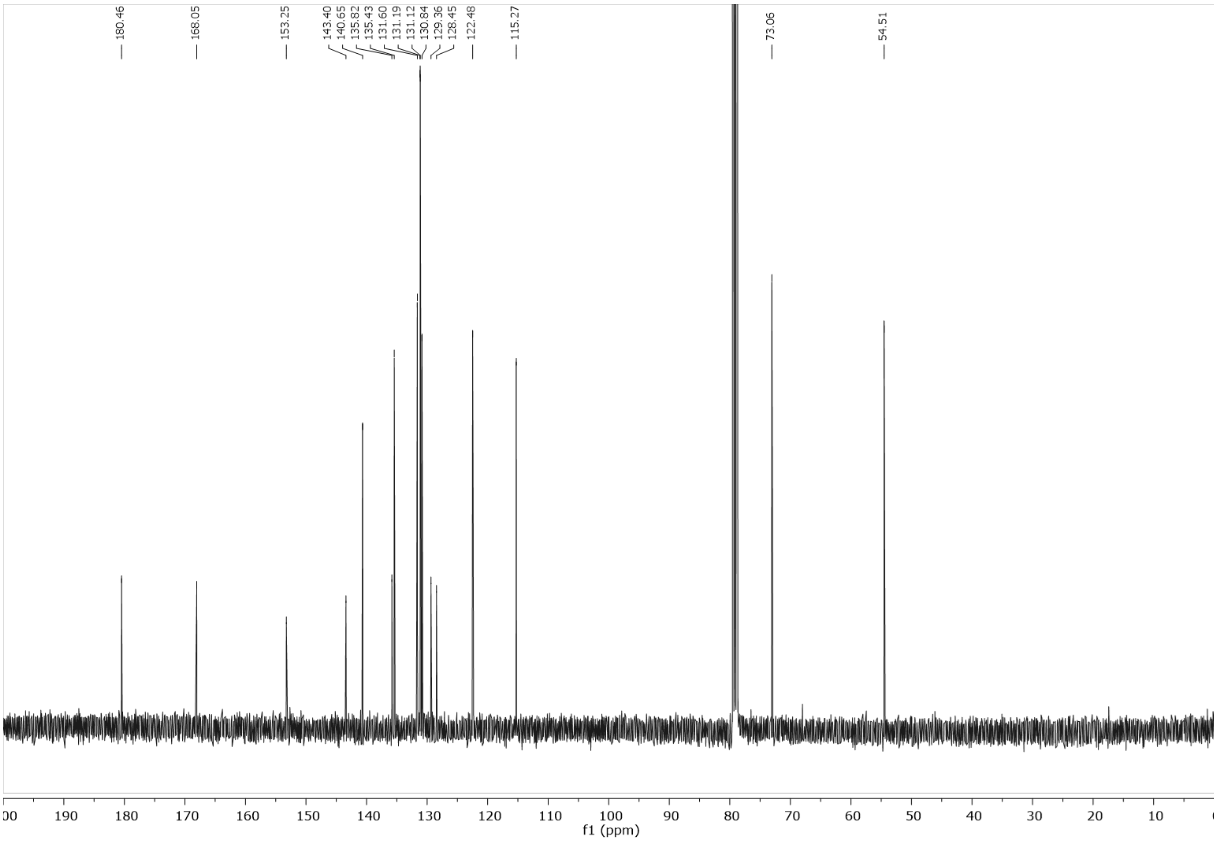

1-Benzyloxycarbonyl-6,7-methylenedioxy-4-quinolone (1j) ($CDCl_3$, ^{1}H 400 MHz, ^{13}C 100 MHz)

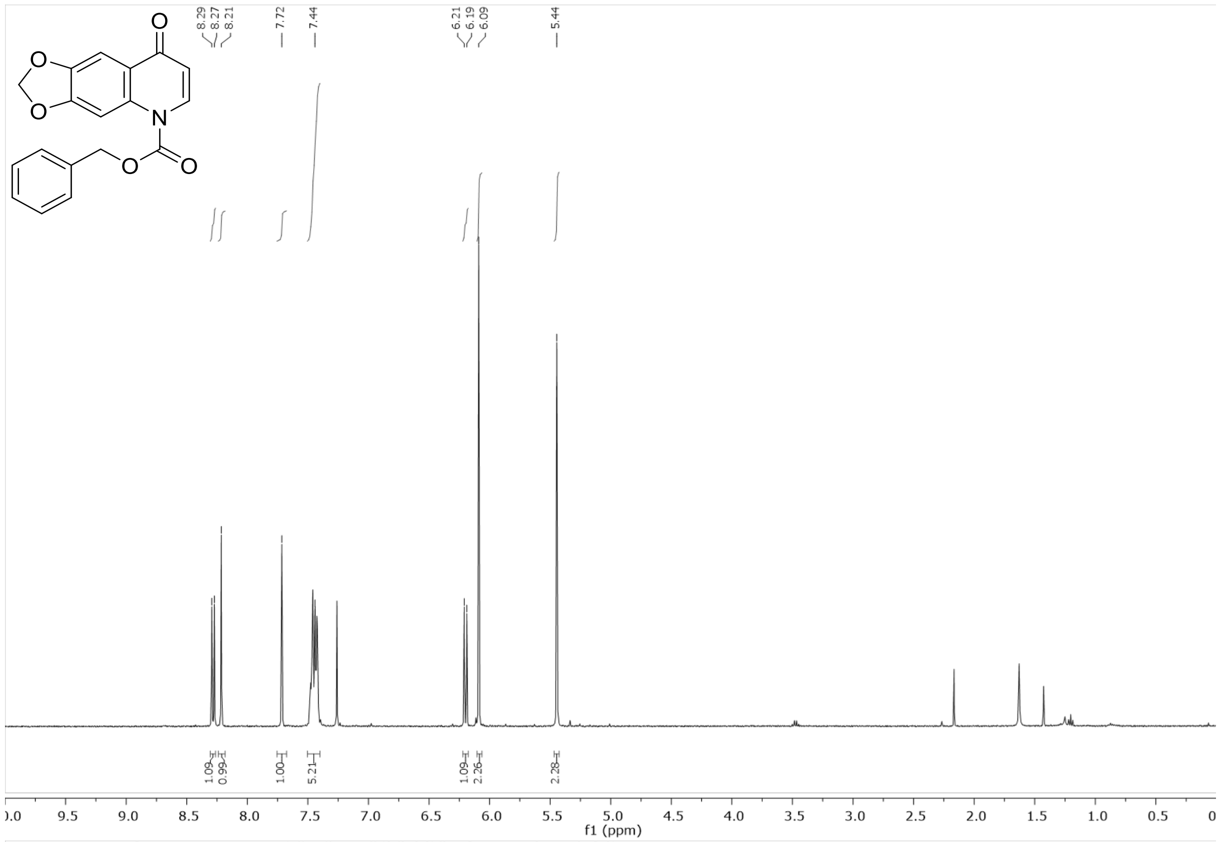

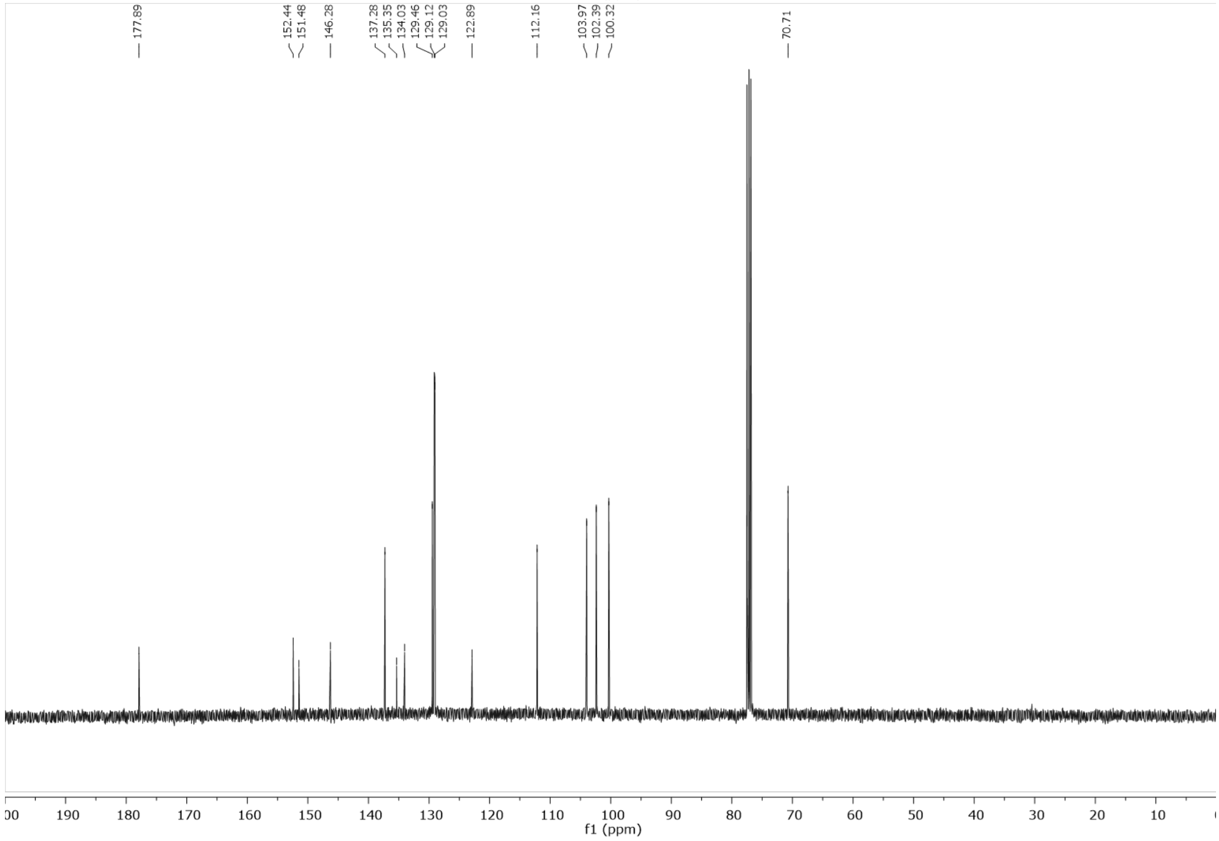

1-Benzyloxycarbonyl-5,7-dimethyl-4-quinolone (1k) ($CDCl_3$, ^{1}H 400 MHz, ^{13}C 100 MHz)

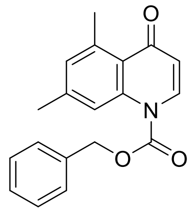

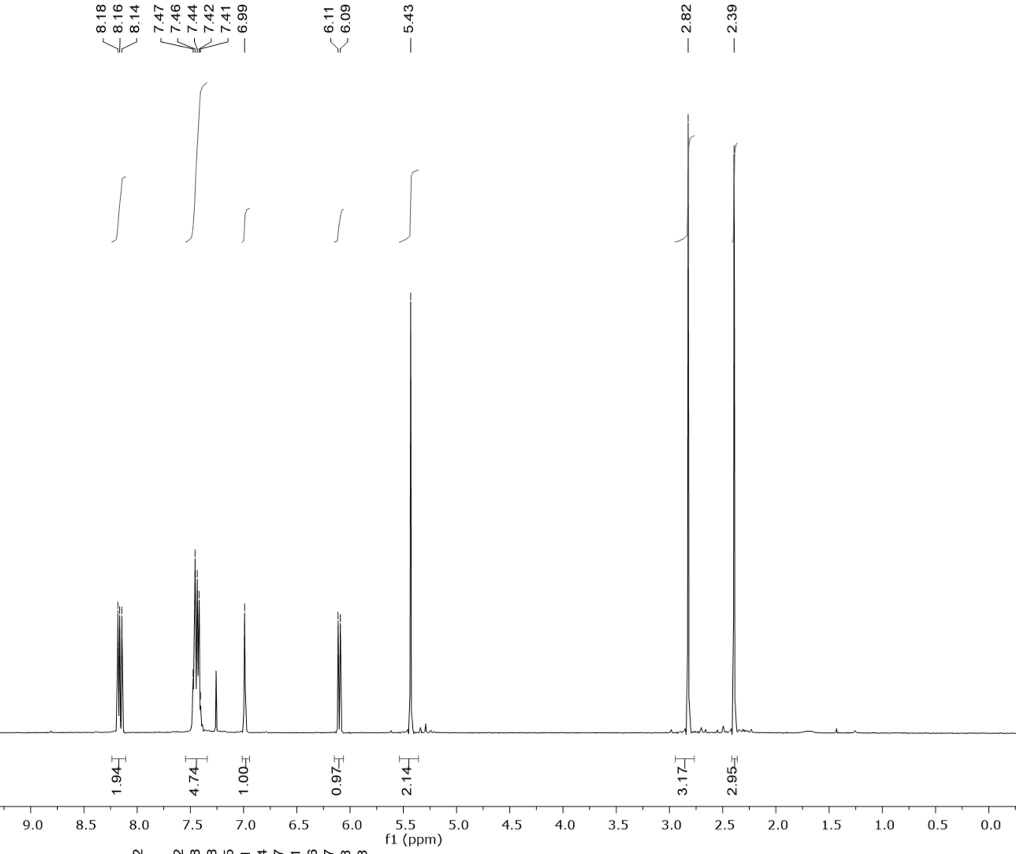

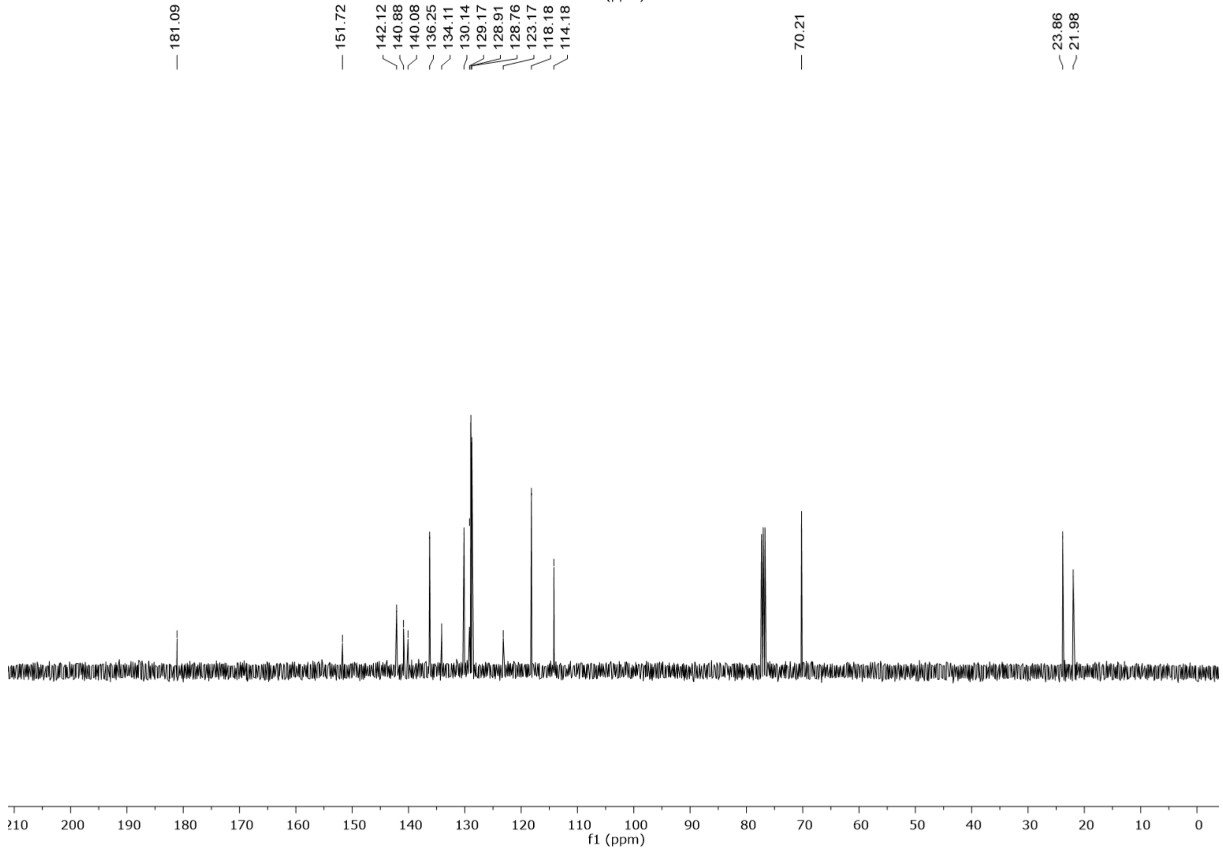

1-Benzyloxycarbonyl-8-fluoro-4-quinolone (1l) ($CDCl_3$, ^{1}H 400 MHz, ^{13}C 100 MHz, ^{19}F 376 MHz)

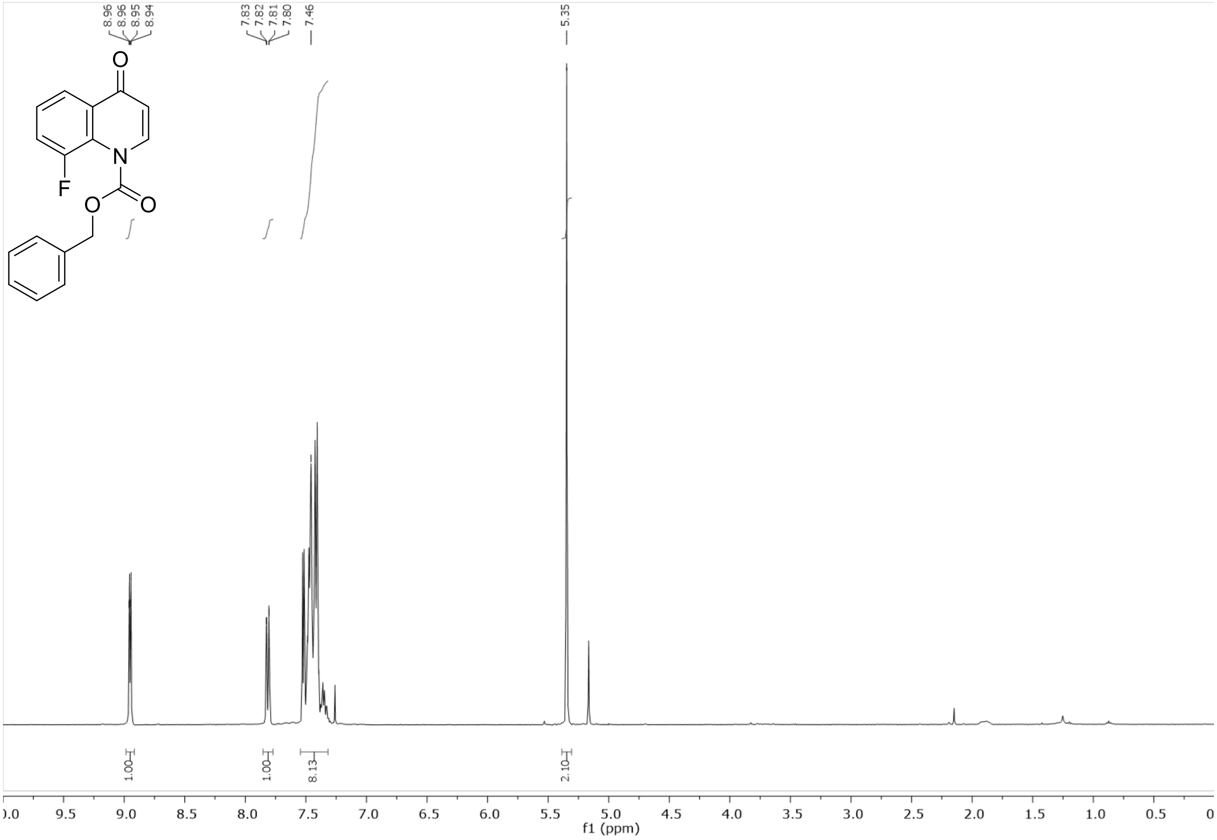

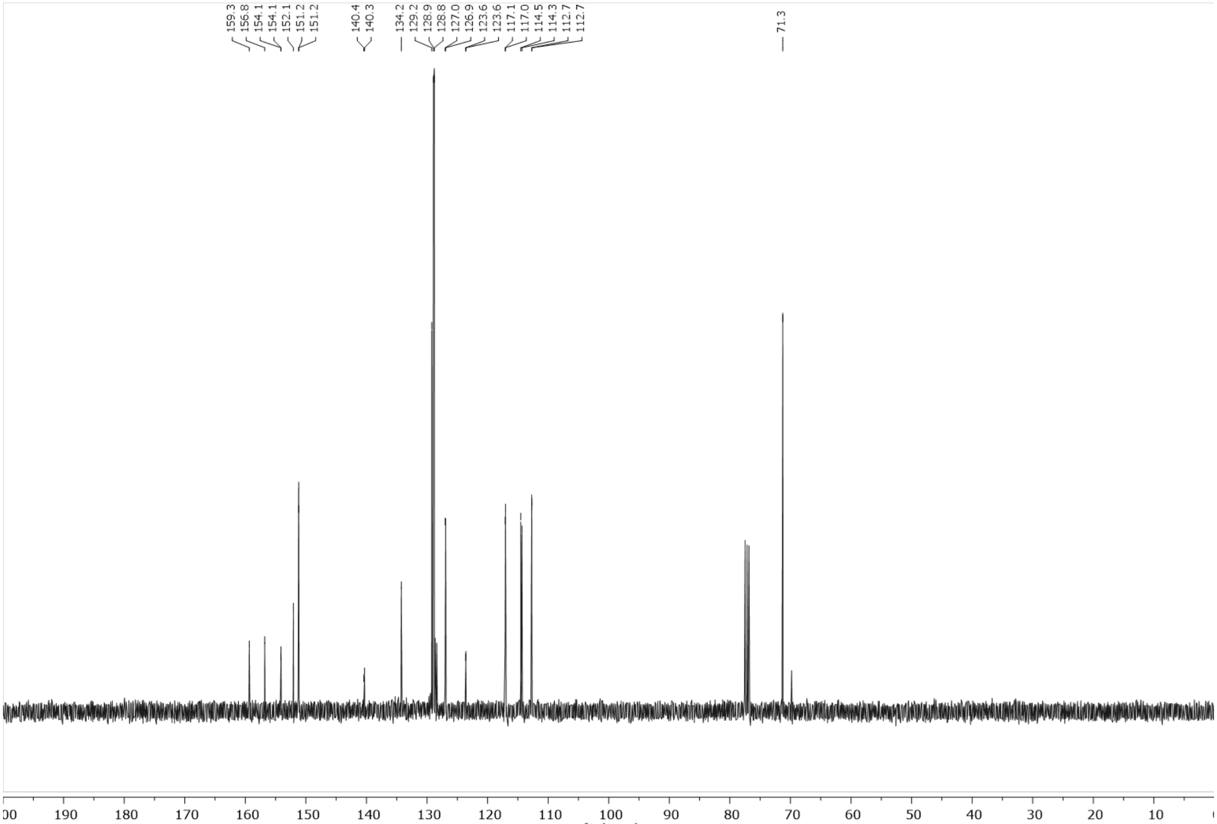

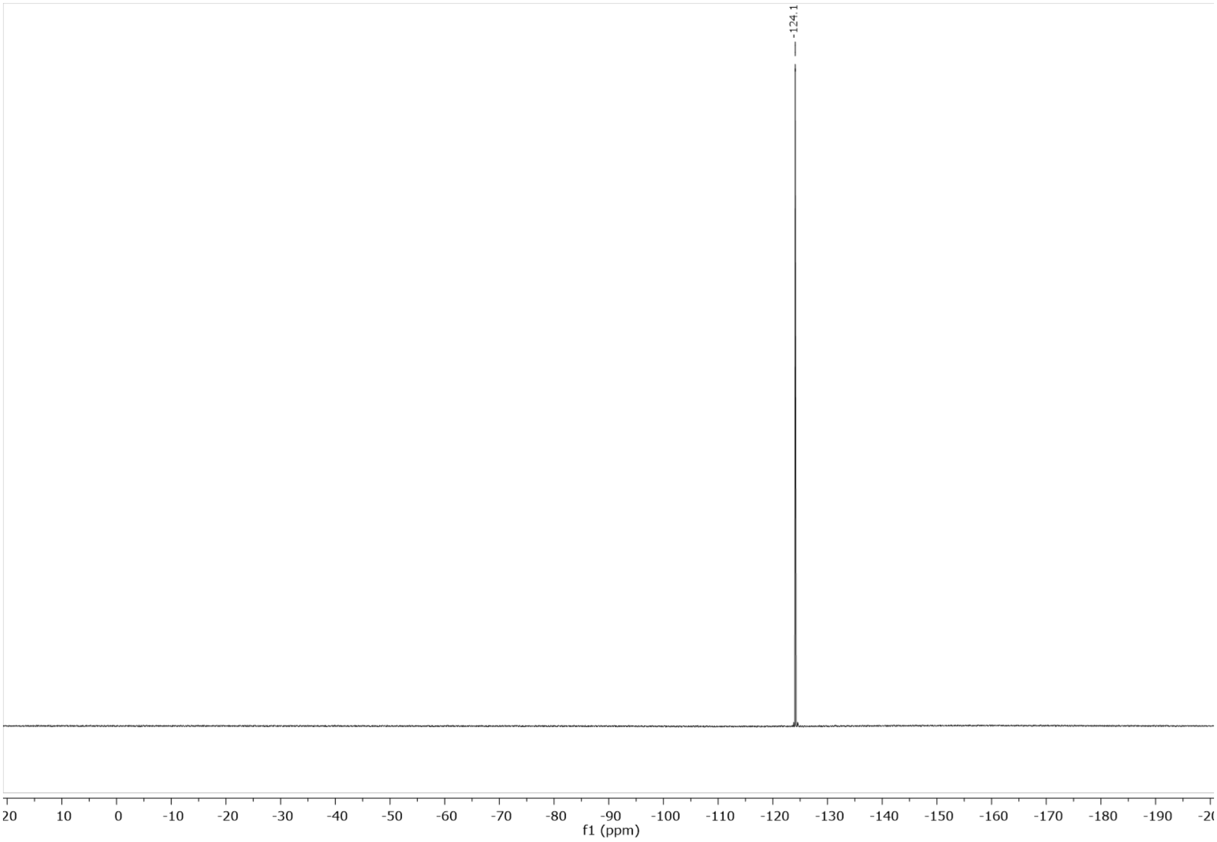
-124.1
20
10
0
-10
-20
-30
-40
-50
-60
-70
-80
-90
-100
-110
-120
-130
-140
-150
-160
-170
-180
-190
f1 (ppm)

NMR spectra of 4-oxo-2-alkynyl-dhydroquinolines **3**

Benzyl 4-oxo-2-(phenylethynyl)-3,4-dihydroquinoline-1(2H)-carboxylate (3aa) ($CDCl_3$, ^1H 400 MHz, ^{13}C 100 MHz)

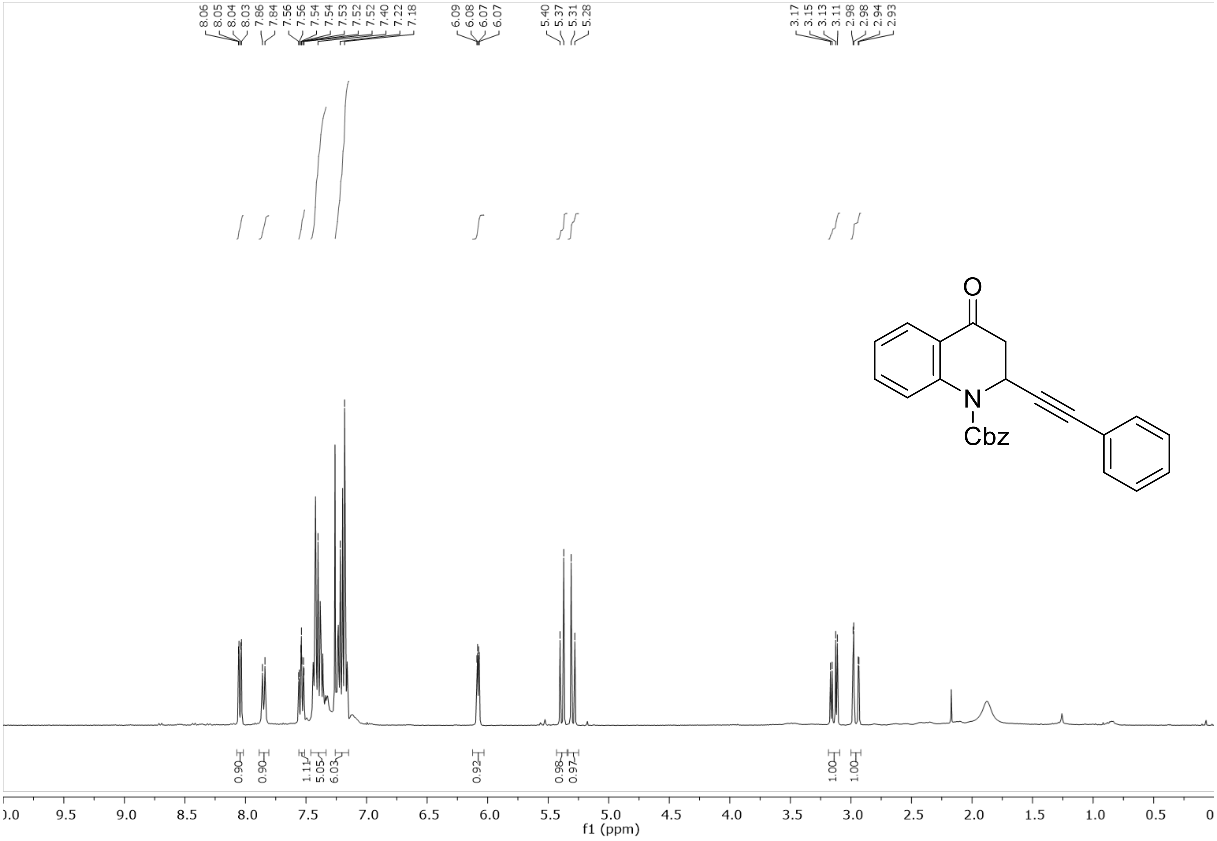

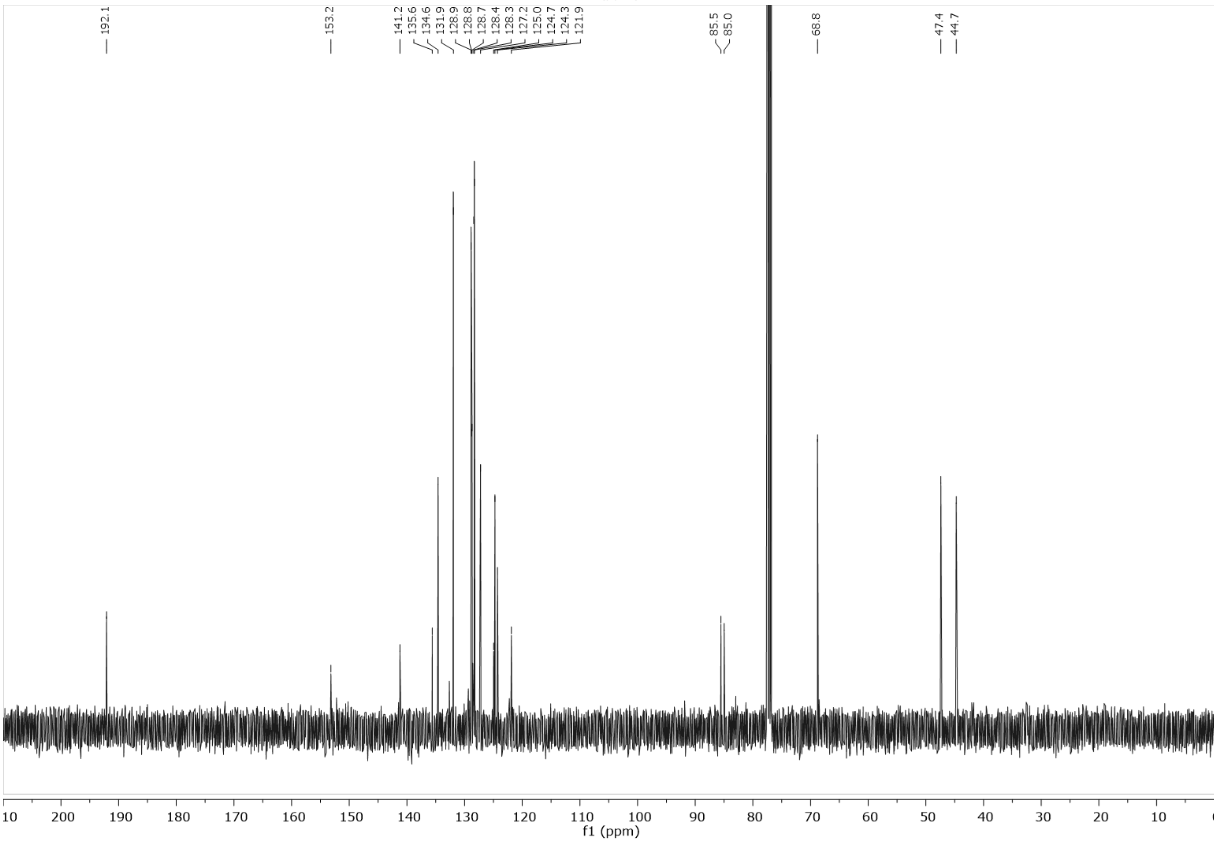

Benzyl 2-((4-methoxyphenyl)ethynyl)-4-oxo-3,4-dihydroquinoline-1(2H)-carboxylate (3ab) ($CDCl_3$, ^{1}H 400 MHz, ^{13}C 100 MHz)

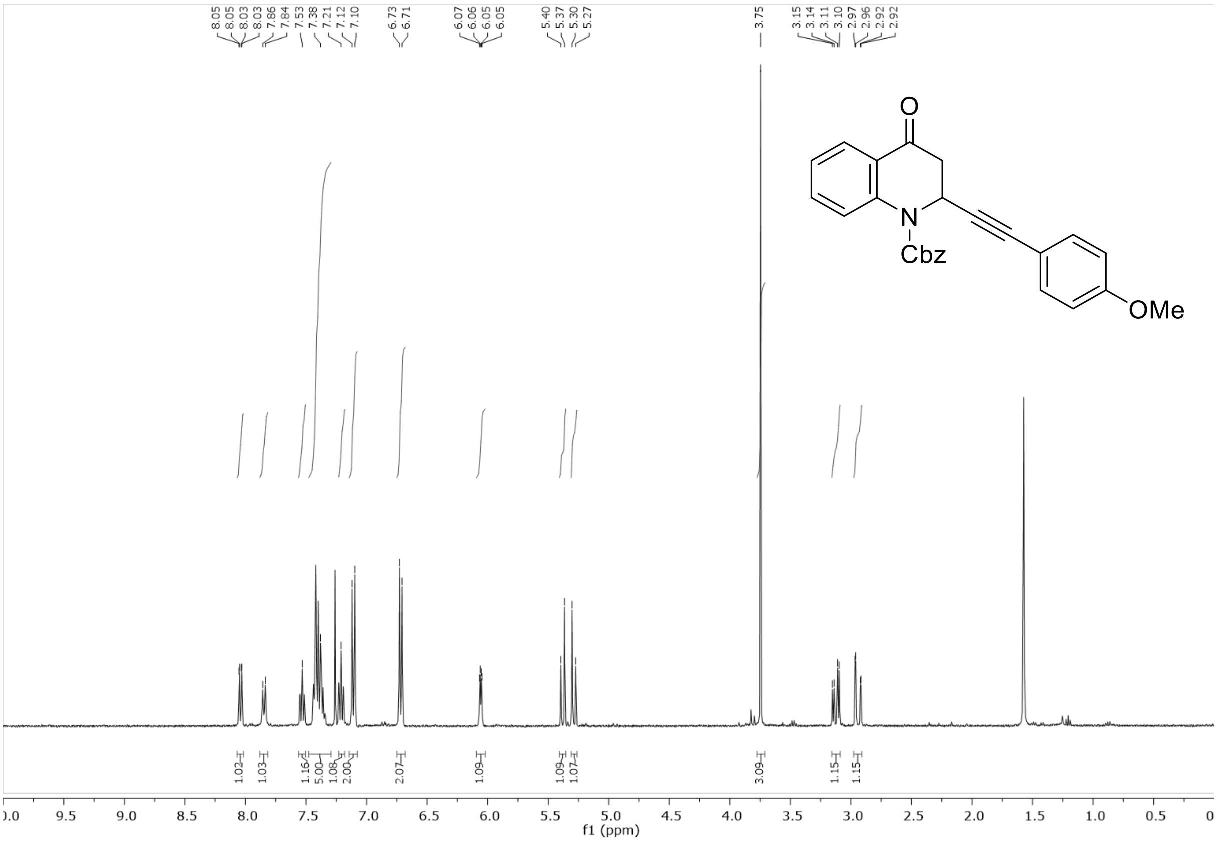

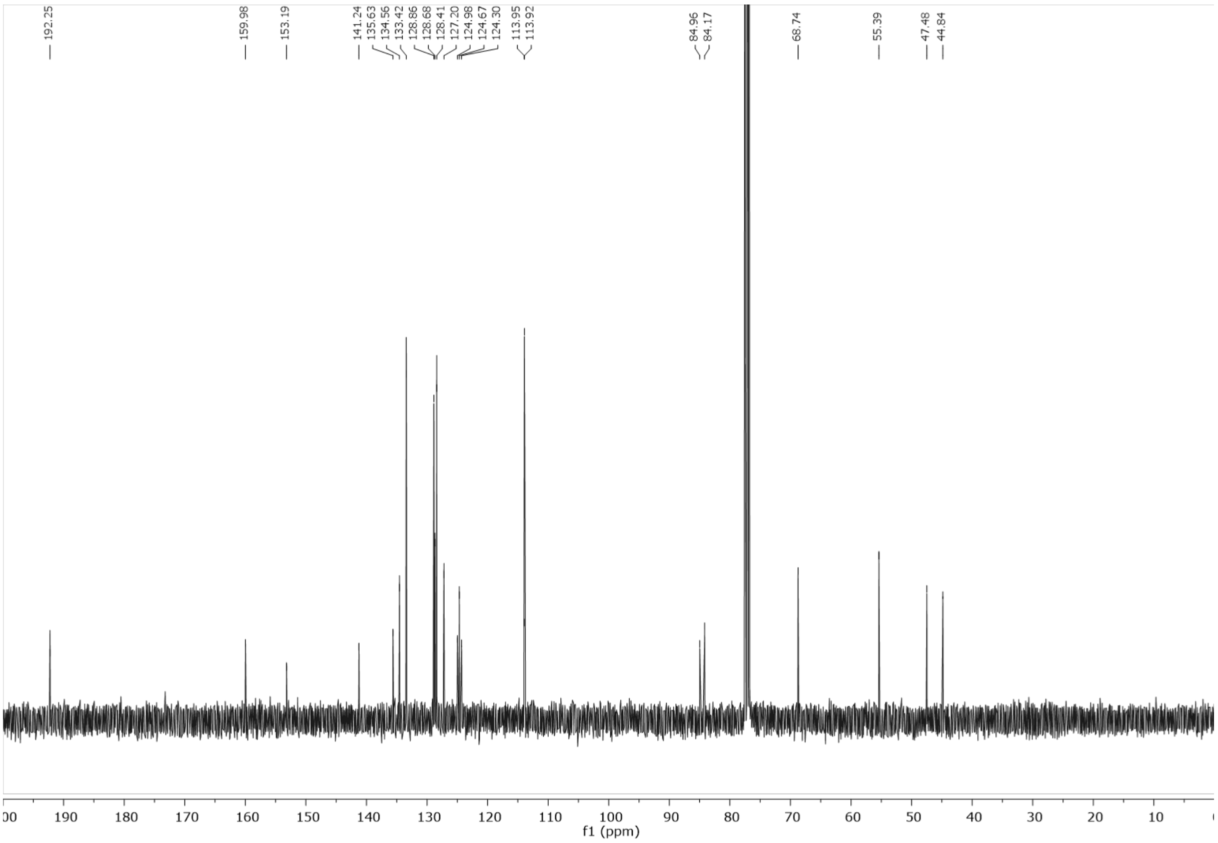

Benzyl 4-oxo-2-(p-tolylethynyl)-3,4-dihydroquinoline-1(2H)-carboxylate (3ac) ($CDCl_3$, ^1H 400 MHz, ^{13}C 100 MHz)

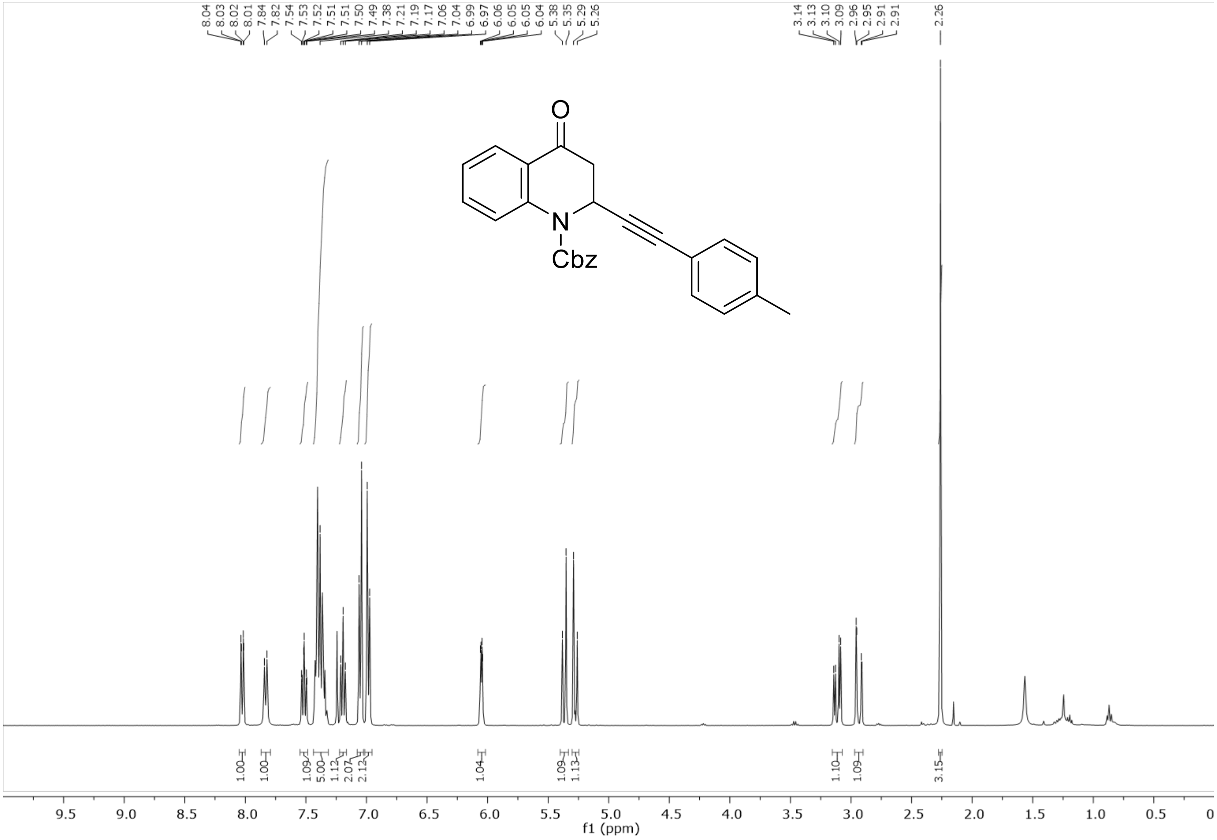

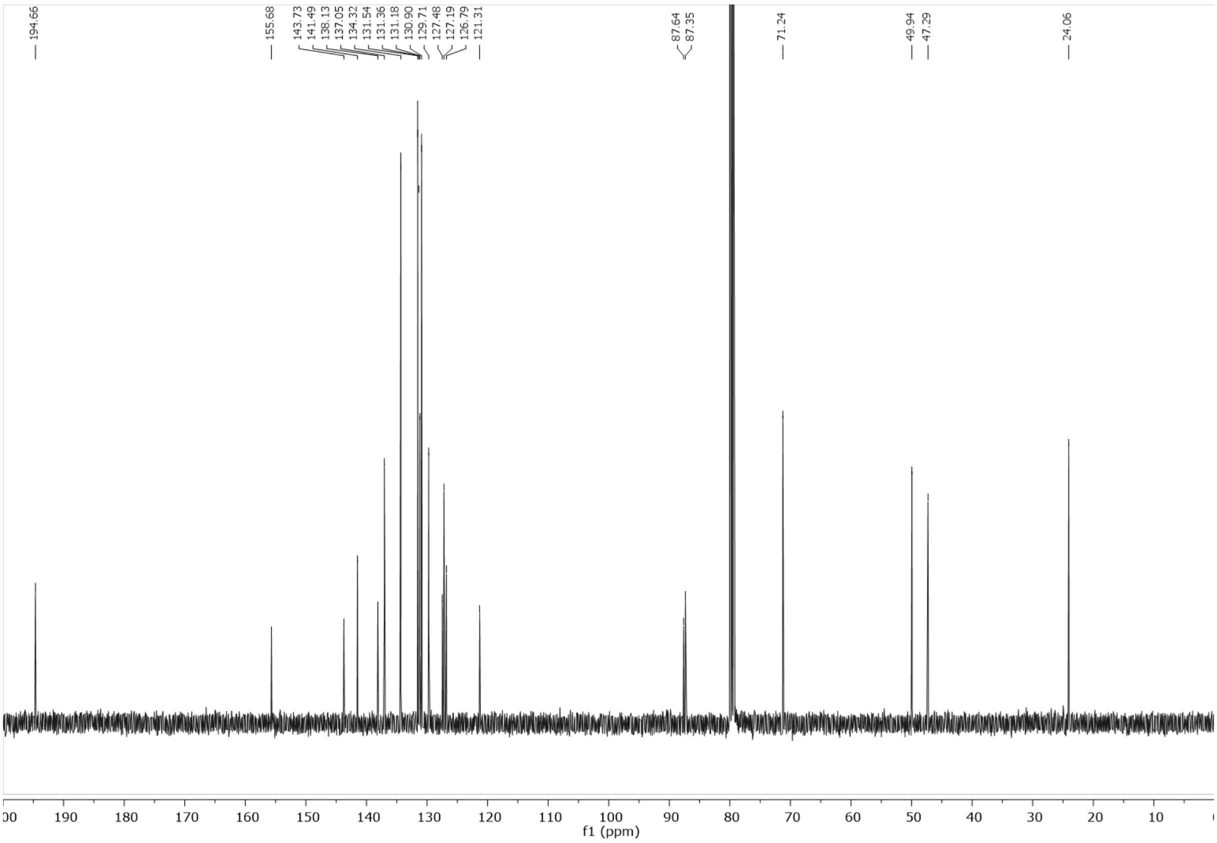

Benzyl 2-((4-fluorophenyl)ethynyl)-4-oxo-3,4-dihydroquinoline-1(2H)-carboxylate (3ad) ($CDCl_3$, ^1H 400 MHz, ^{13}C 100 MHz, ^{19}F 376 MHz)

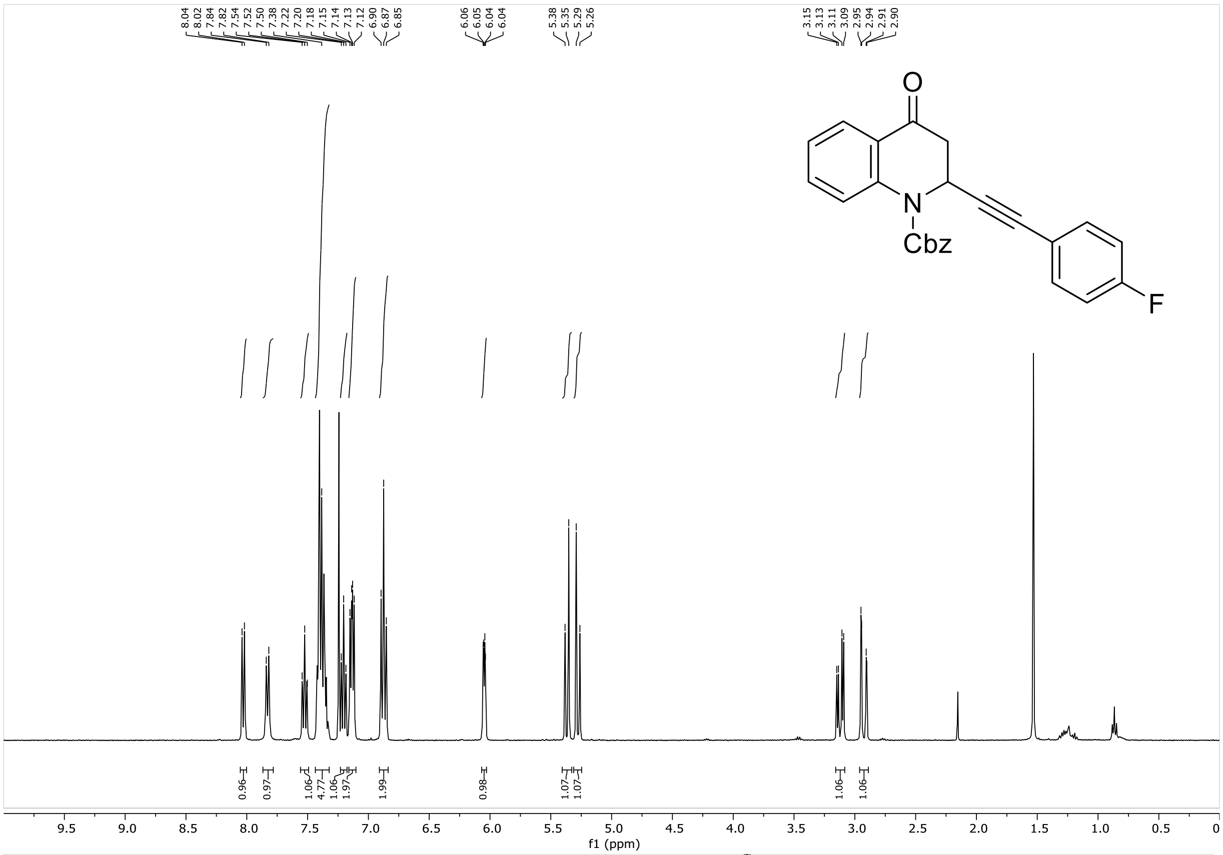

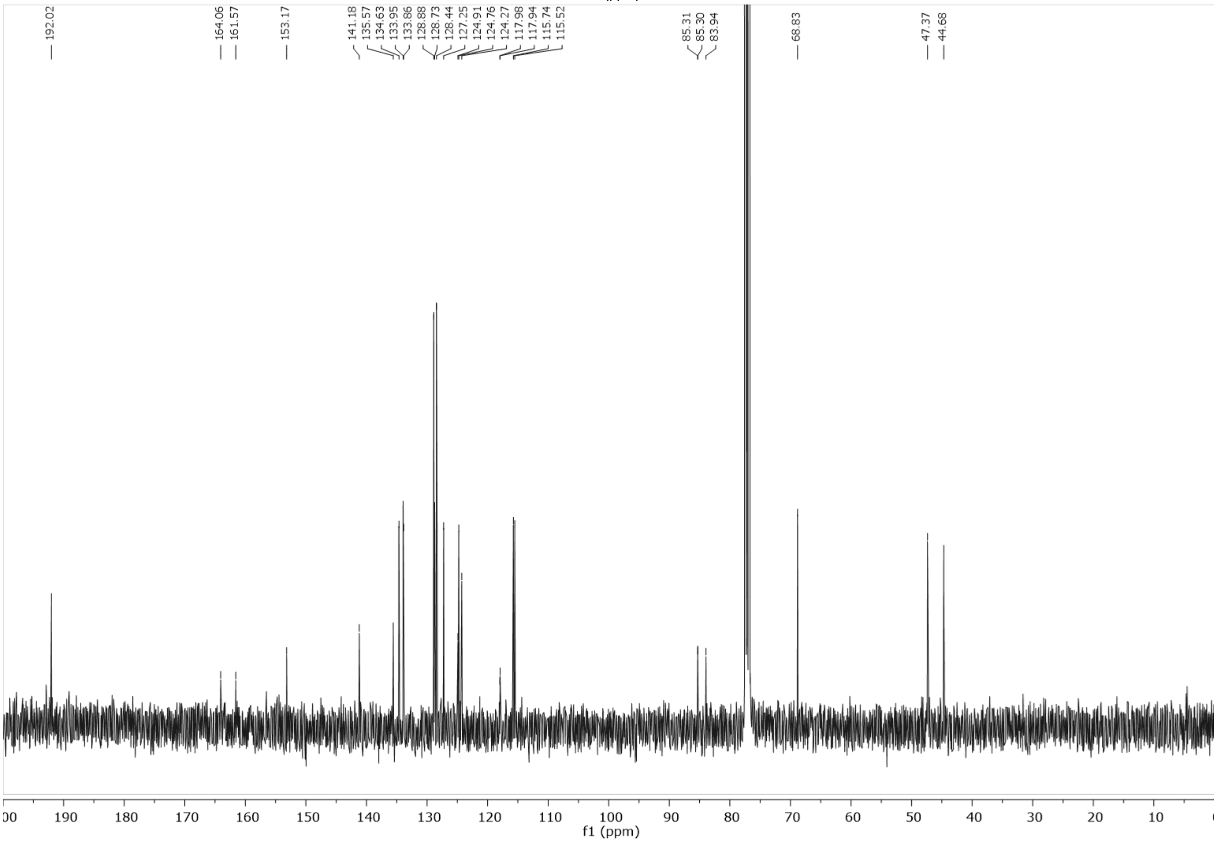

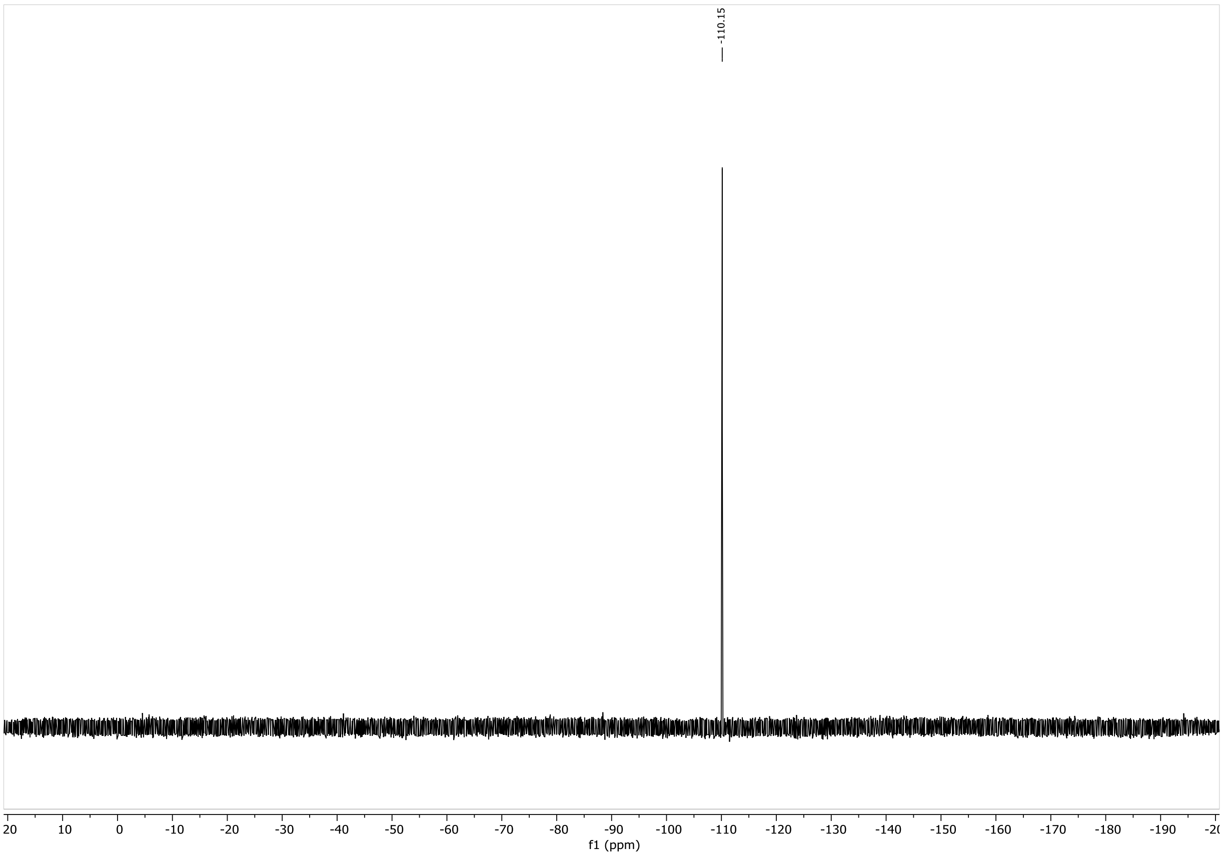
-110.15
20
10
0
-10
-20
-30
-40
-50
-60
-70
-80
-90
-100
-110
-120
-130
-140
-150
-160
-170
-180
-190
f1 (ppm)

Benzyl 4-oxo-2-((4-(trifluoromethyl)phenyl)ethynyl)-3,4-dihydroquinoline-1(2H)-carboxylate (3ae) ($CDCl_3$, ^1H 400 MHz, ^{13}C 100 MHz, ^{19}F 376 MHz)

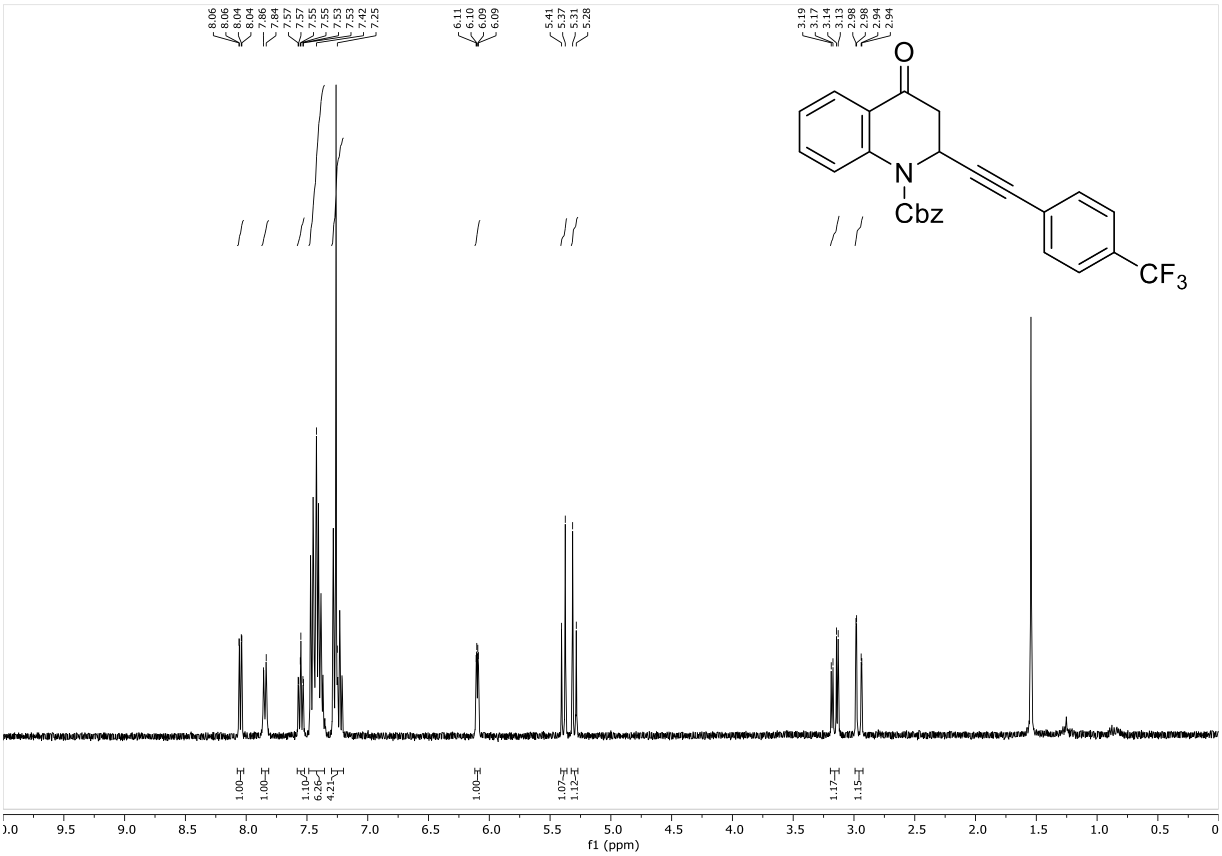

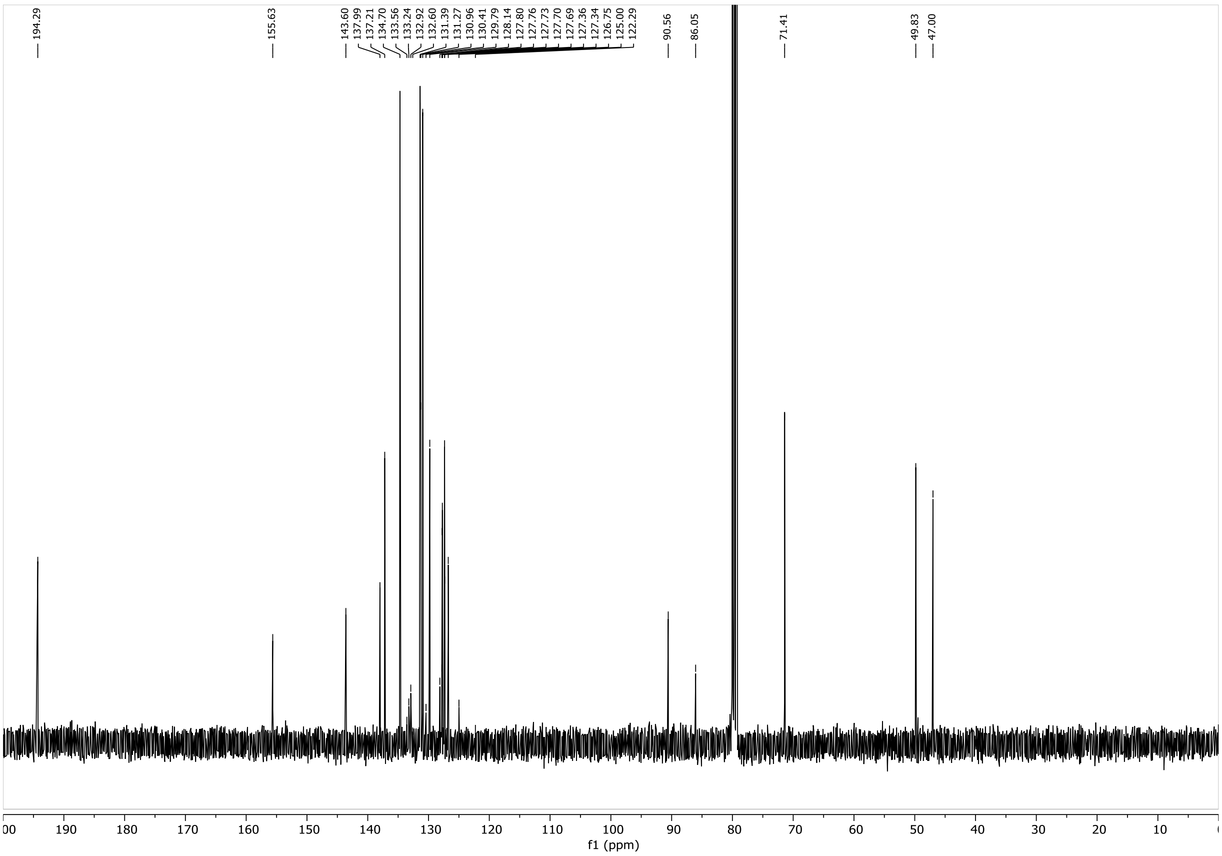

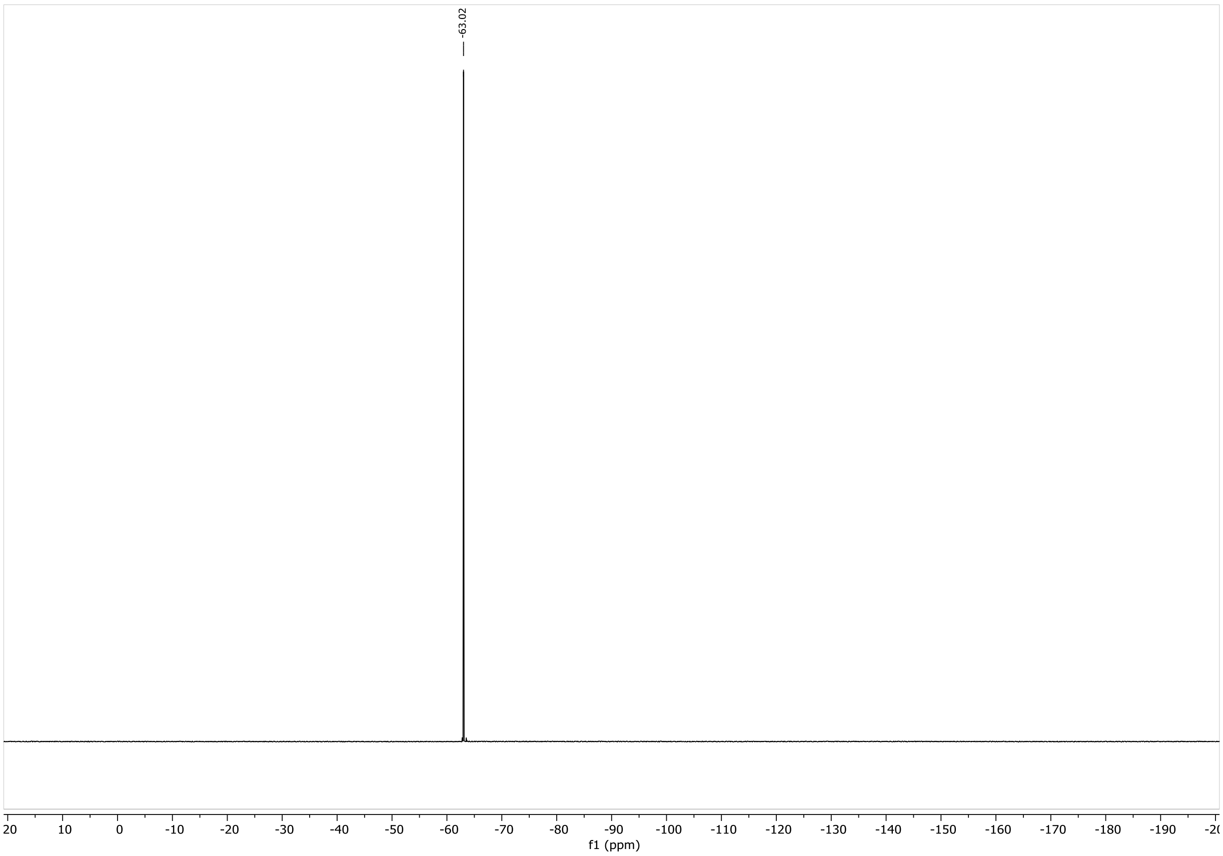

-63.02
20
10
0
-10
-20
-30
-40
-50
-60
-70
-80
-90
-100
-110
-120
-130
-140
-150
-160
-170
-180
-190
f1 (ppm)

(*R*)–Benzyl 2-((3-methoxyphenyl)ethynyl)-4-oxo-3,4-dihydroquinoline-1(2H)-carboxylate (3af) ($CDCl_3$, ^{1}H 400 MHz, ^{13}C 100 MHz)

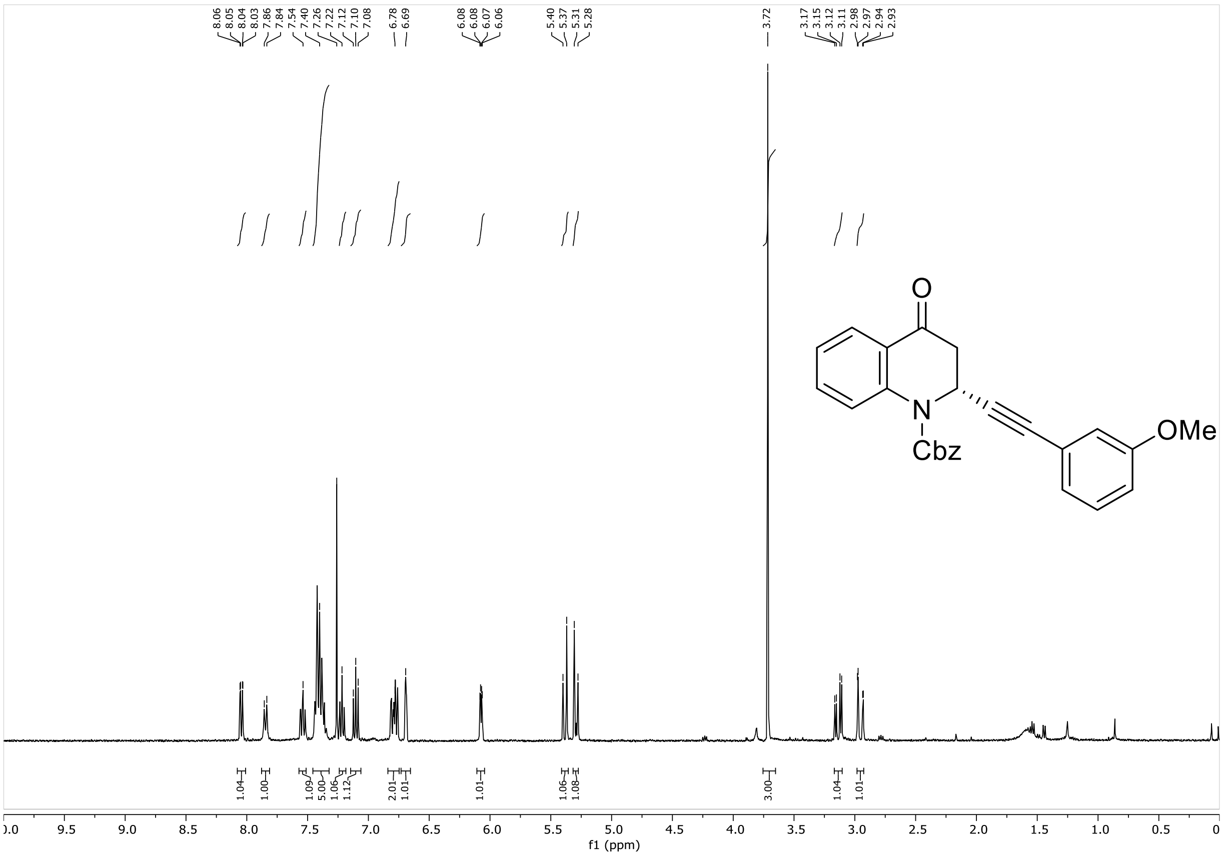

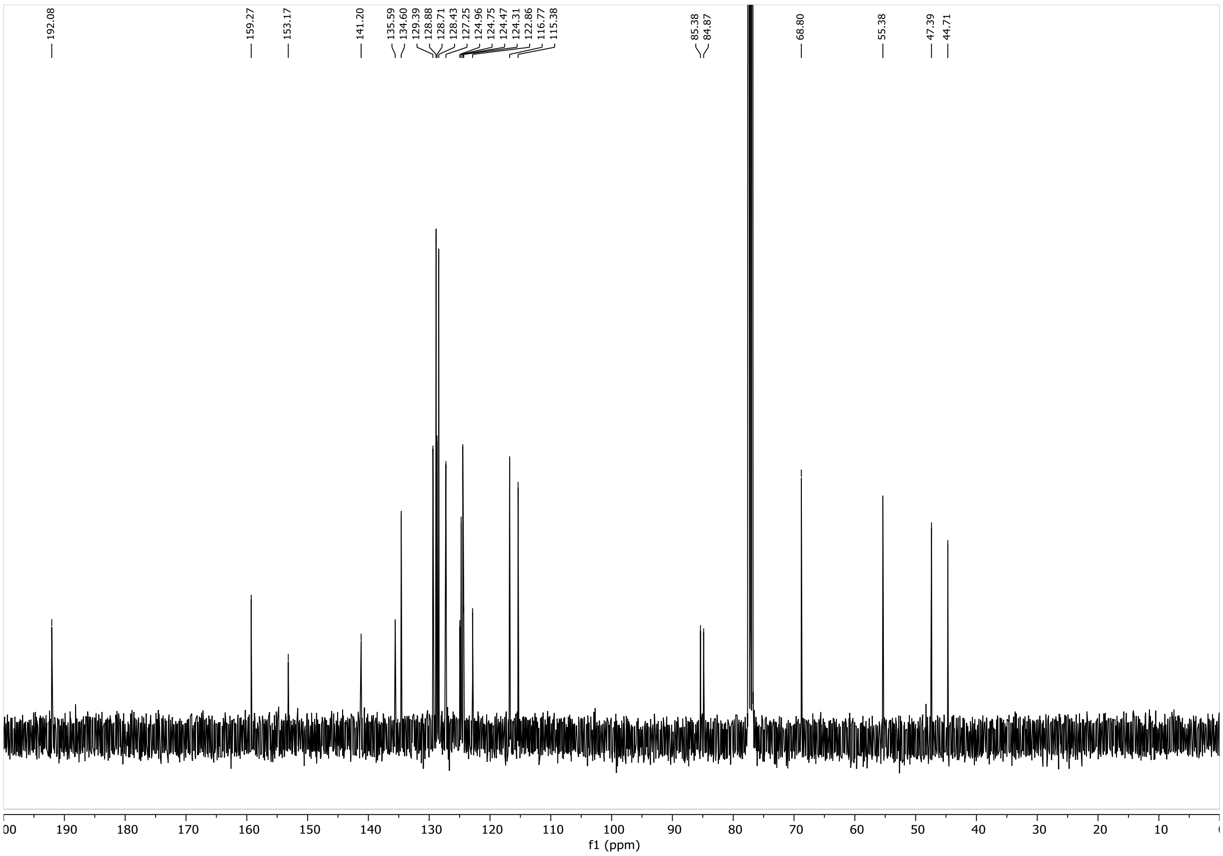

(*R*)–Benzyl 4-oxo-2-(m-tolylethynyl)-3,4-dihydroquinoline-1(2H)-carboxylate (3ag) ($CDCl_3$, ^{1}H 400 MHz, ^{13}C 100 MHz)

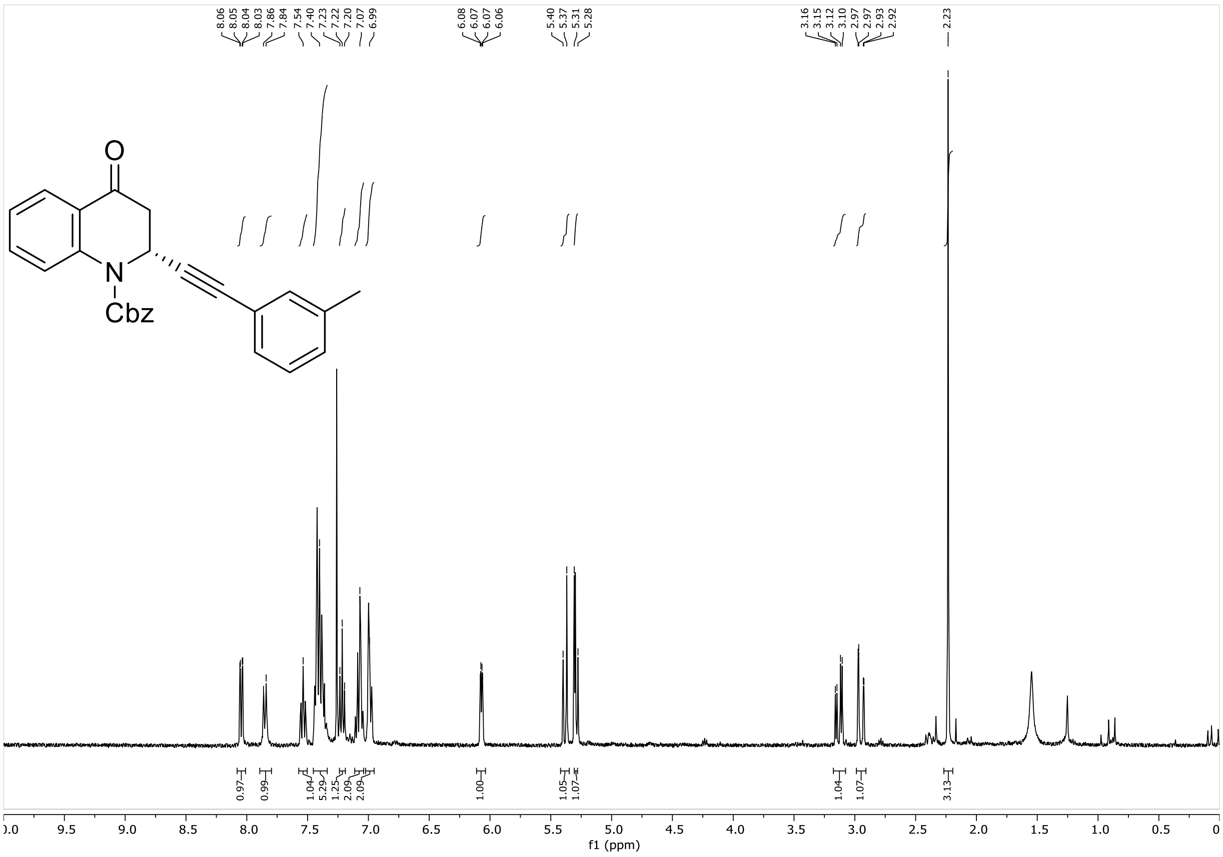

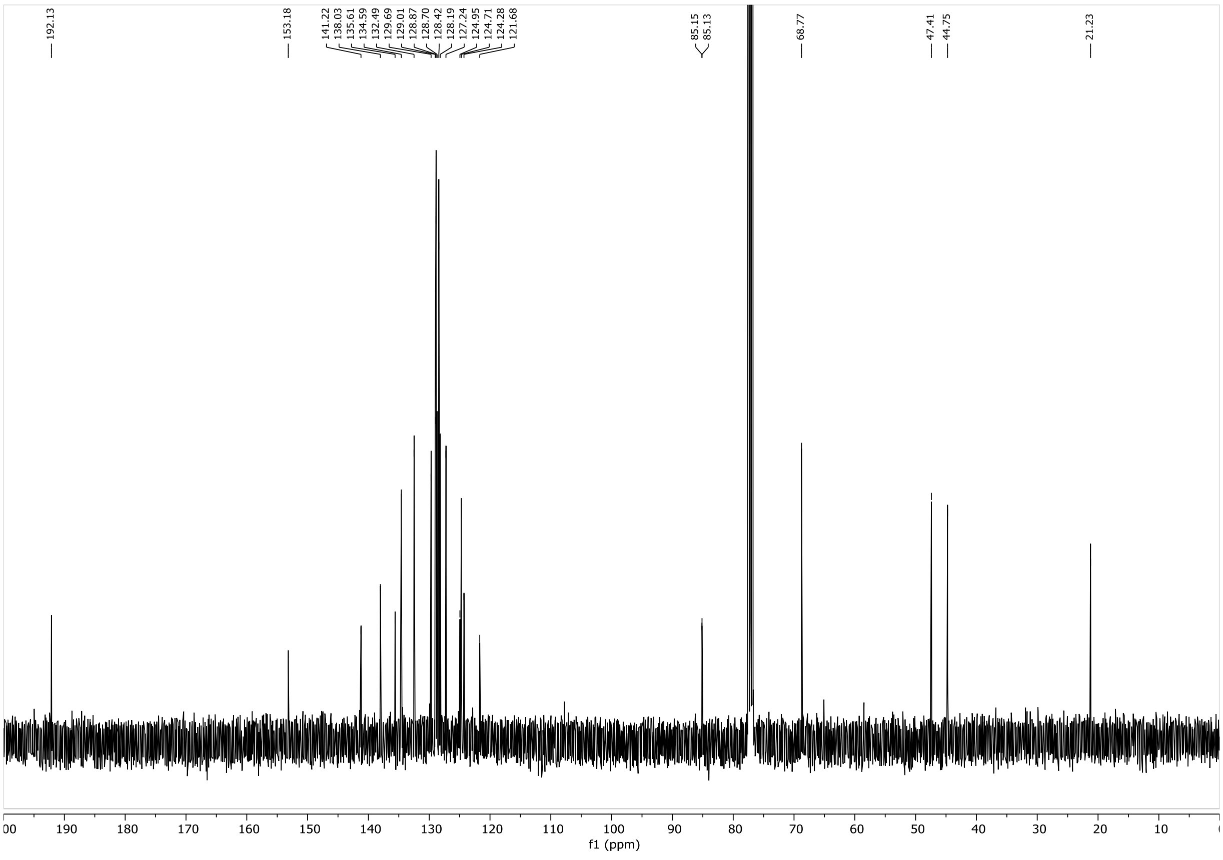

Benzyl 2-((3-chlorophenyl)ethynyl)-4-oxo-3,4-dihydroquinoline-1(2H)-carboxylate (3ah) ($CDCl_3$, ^1H 400 MHz, ^{13}C 100 MHz)

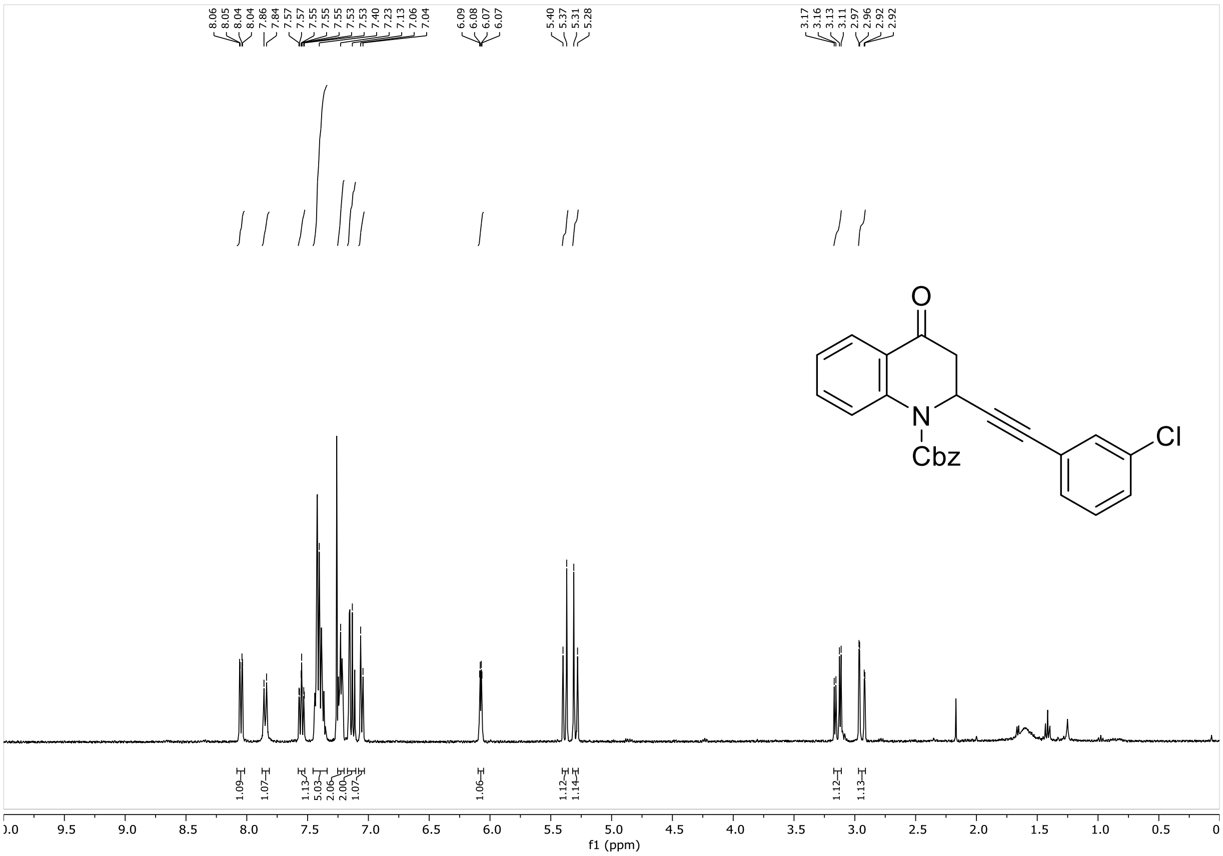

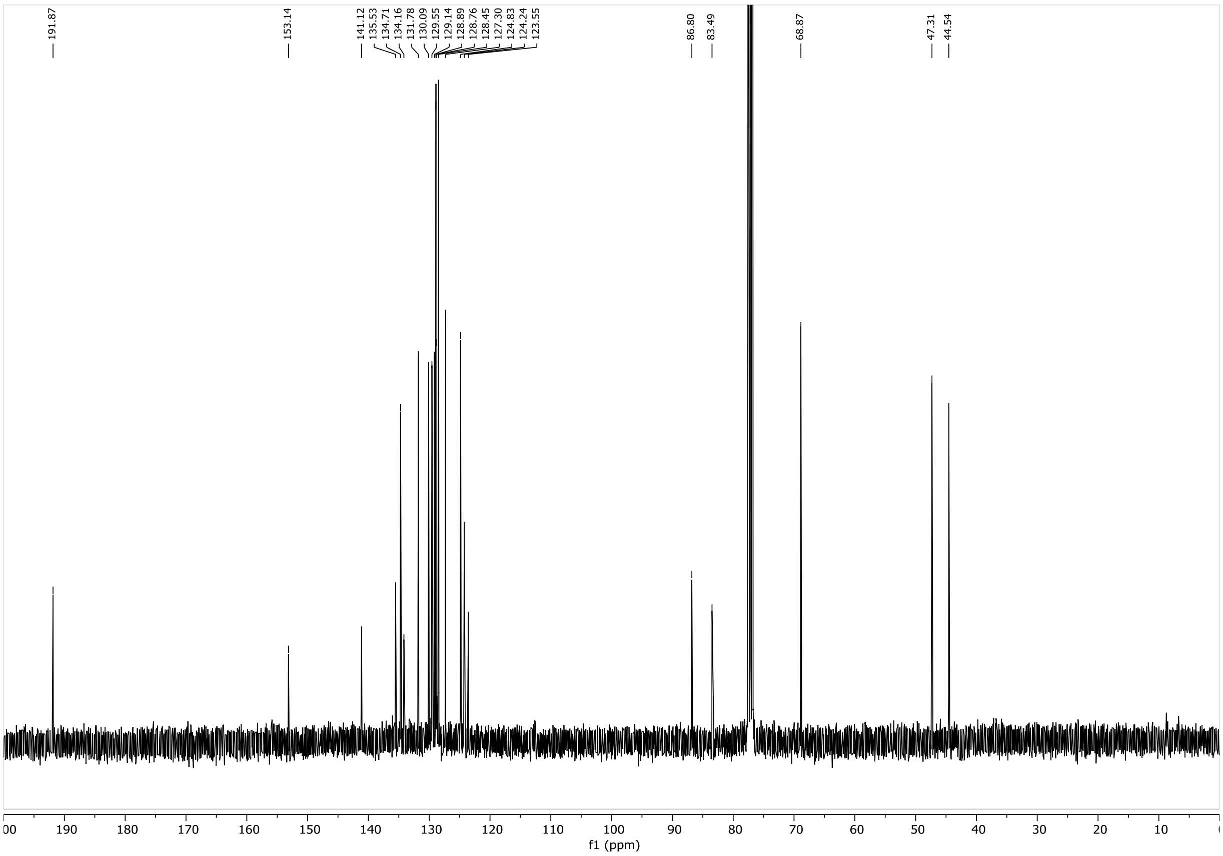

Benzyl 4-oxo-2-((3-(trifluoromethyl)phenyl)ethynyl)-3,4-dihydroquinoline-1(2H)-carboxylate (3ai)
($CDCl_3$, ^{1}H 400 MHz, ^{13}C 100 MHz, ^{19}F 376 MHz)

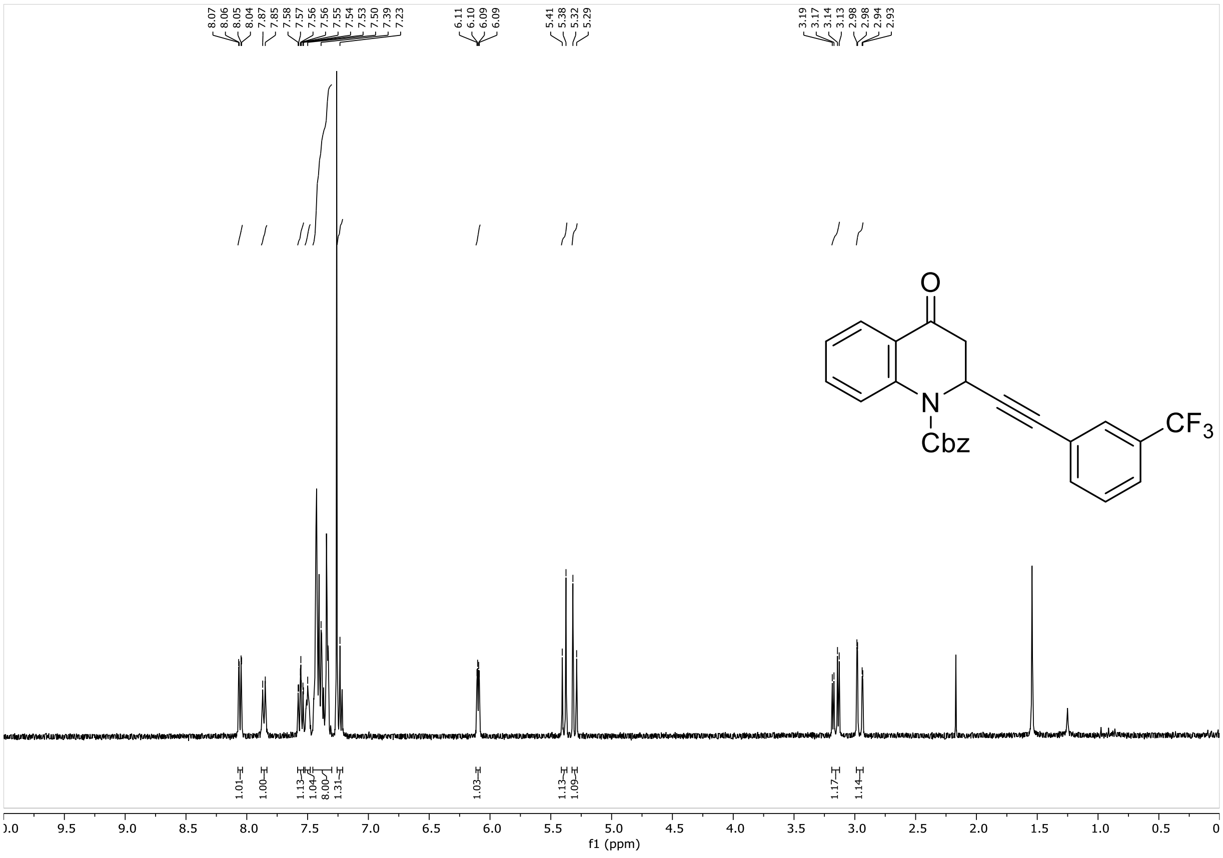

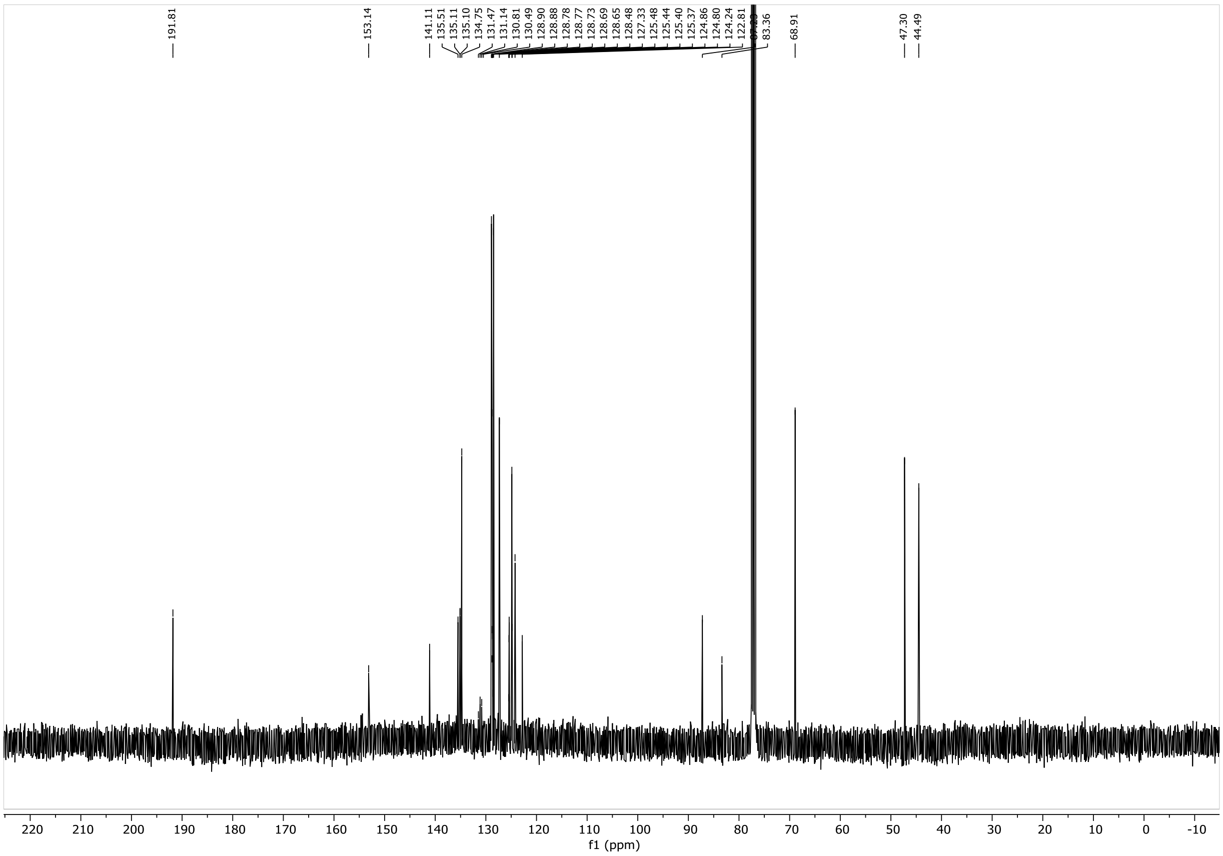
191.81
153.14
141.11
135.51
135.11
135.10
134.75
131.47
131.14
130.81
130.49
128.90
128.88
128.78
128.77
128.73
128.69
128.65
128.48
127.33
125.48
125.44
125.40
125.37
124.86
124.80
124.24
122.81
83.36
68.91
47.30
44.49
f1 (ppm)

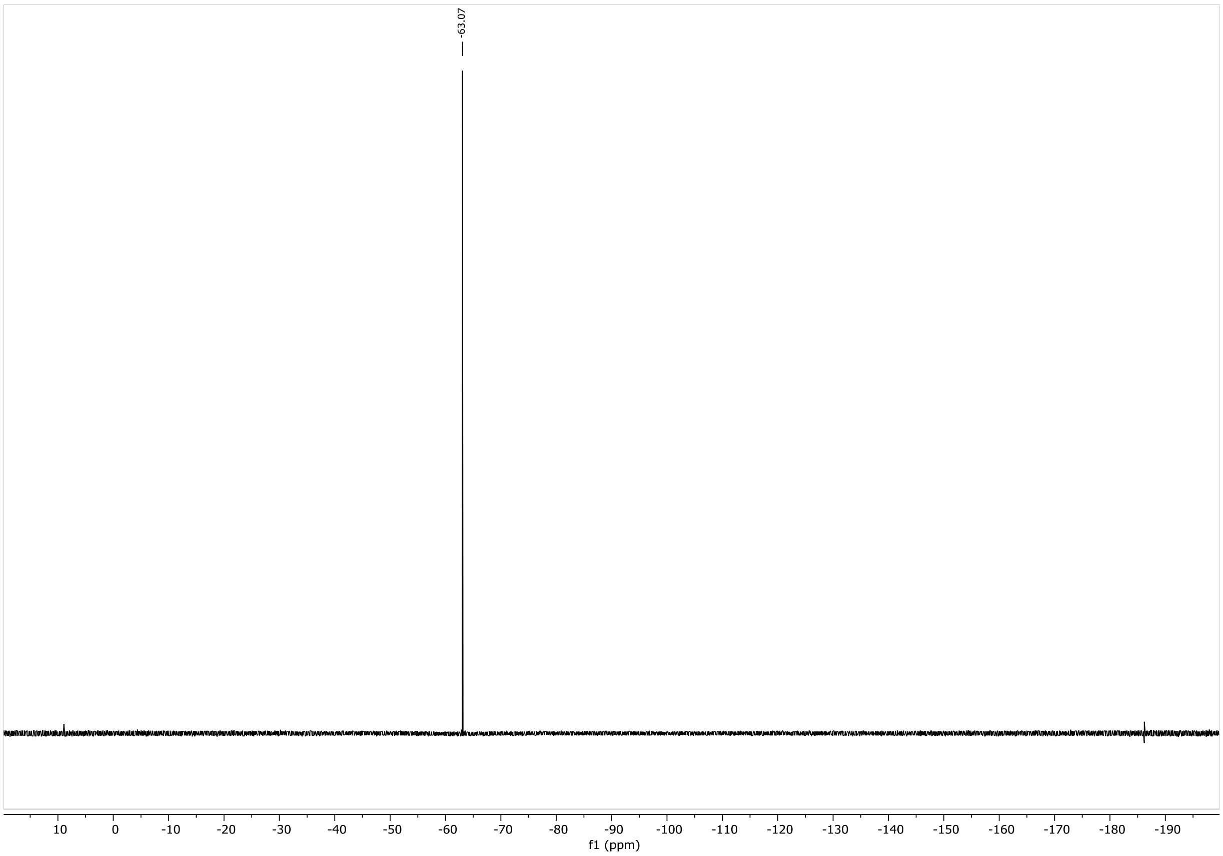
-63.07
f1 (ppm)

(*R*)–Benzyl 4-oxo-2-(o-tolylethynyl)-3,4-dihydroquinoline-1(2H)-carboxylate (3aj) ($CDCl_3$, ^{1}H 400 MHz, ^{13}C 100 MHz)

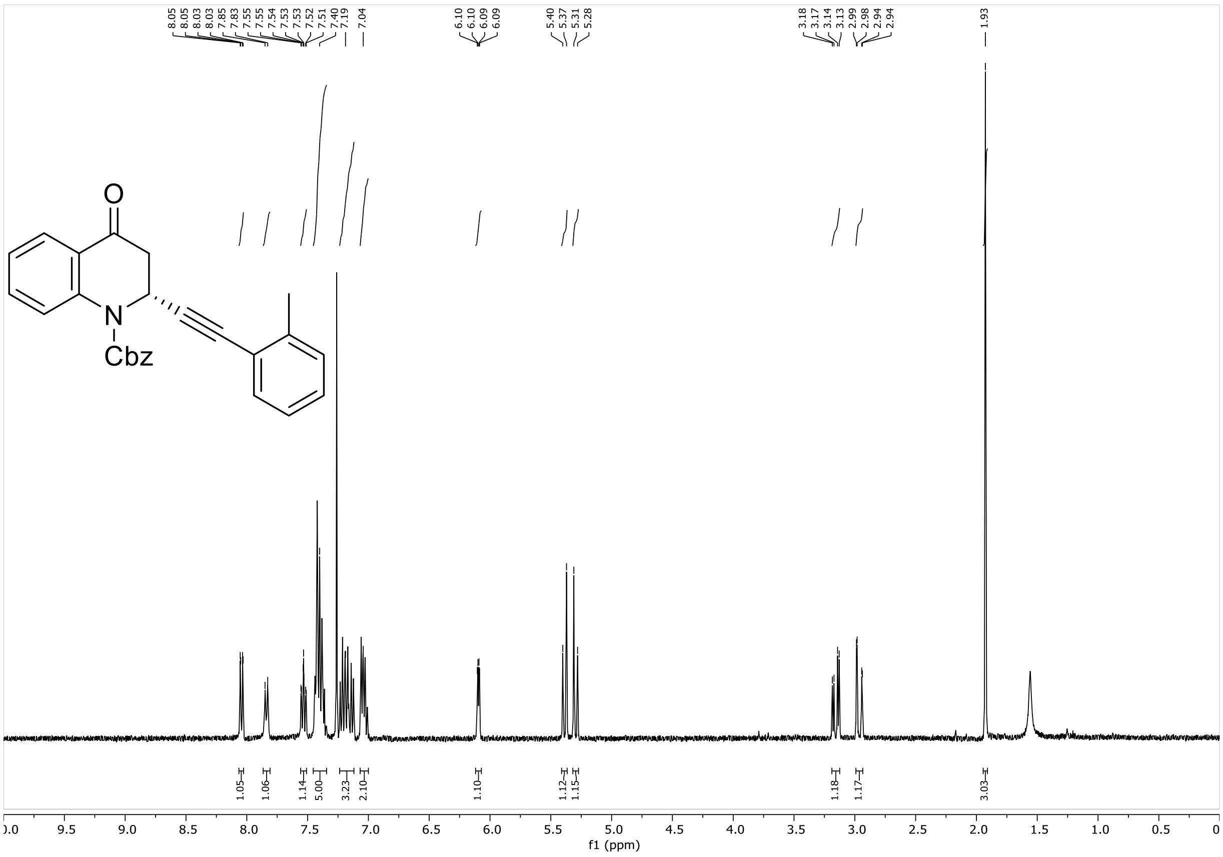

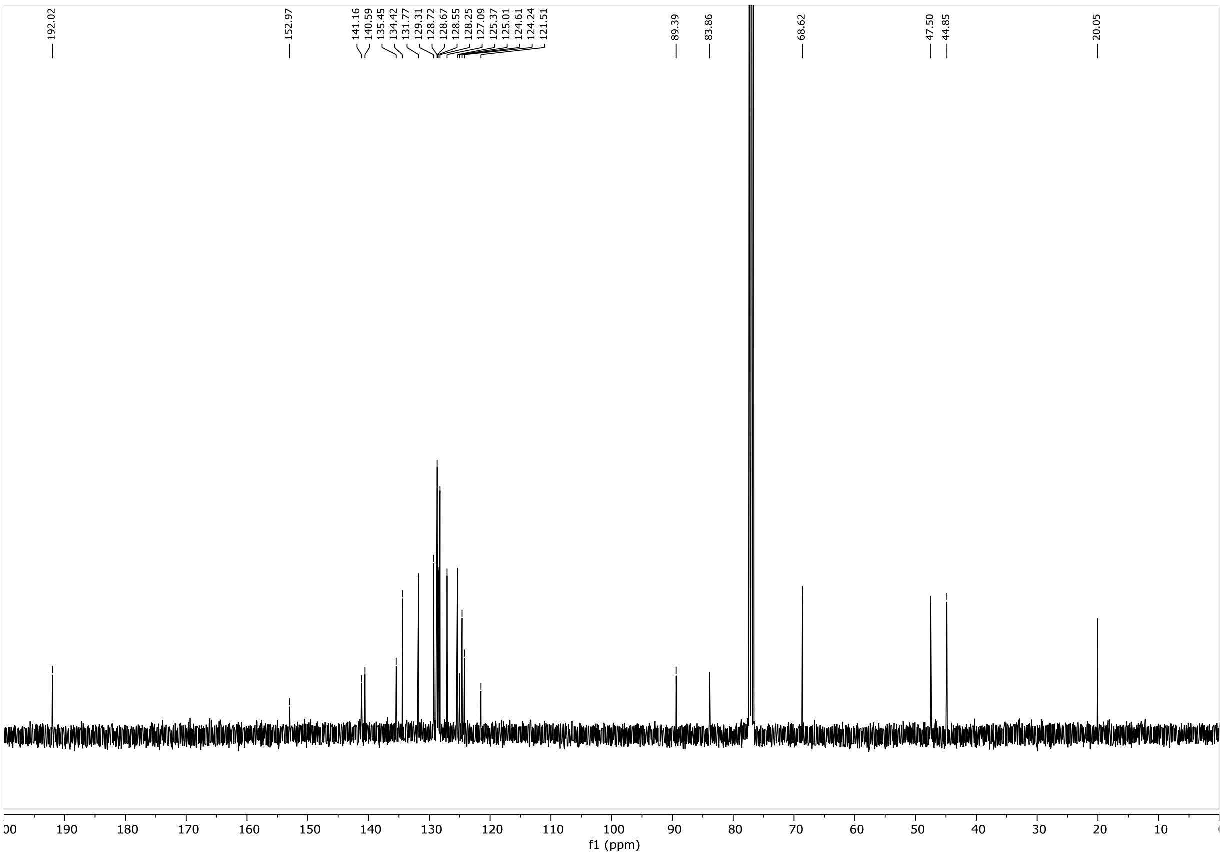

(*R*)–Benzyl 4-oxo-2-(thiophen-3-ylethynyl)-3,4-dihydroquinoline-1(2H)-carboxylate (3ak) ($CDCl_3$, ^1H 400 MHz, ^{13}C 100 MHz)

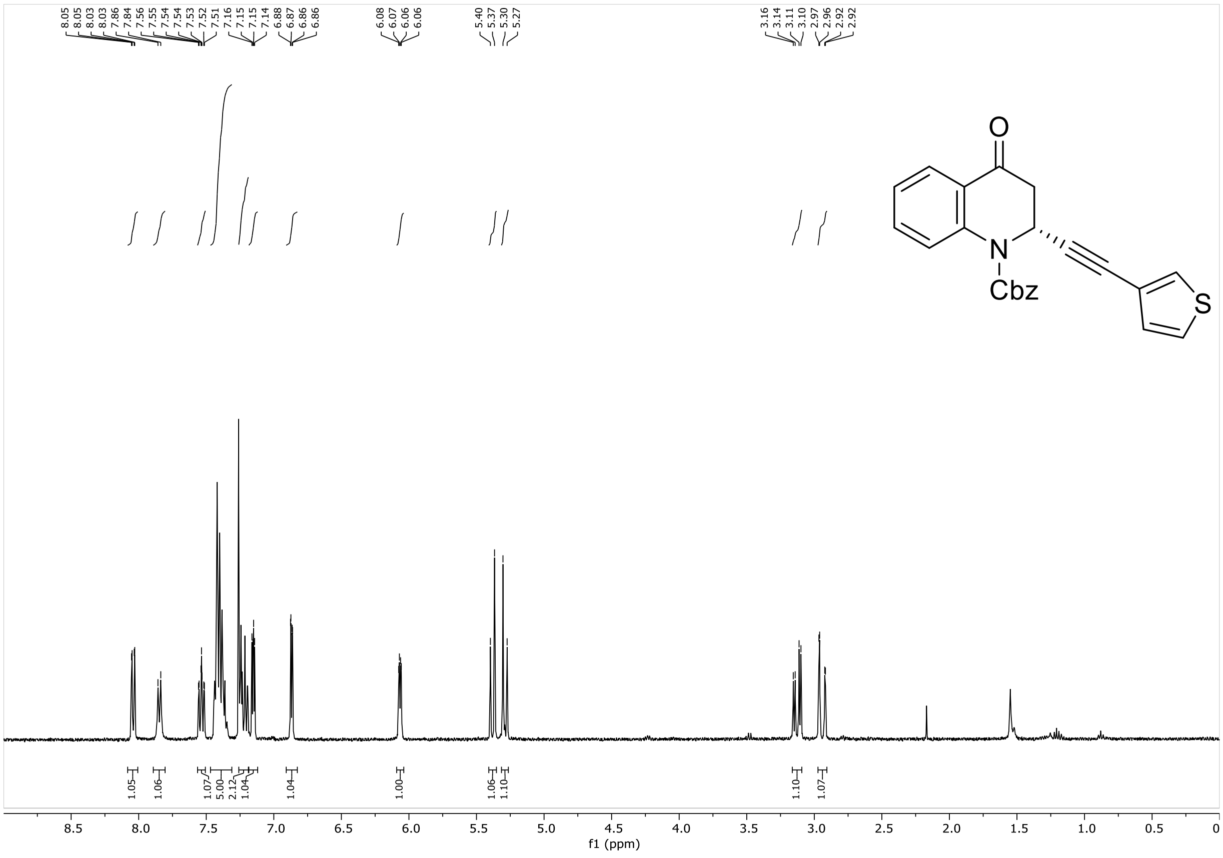

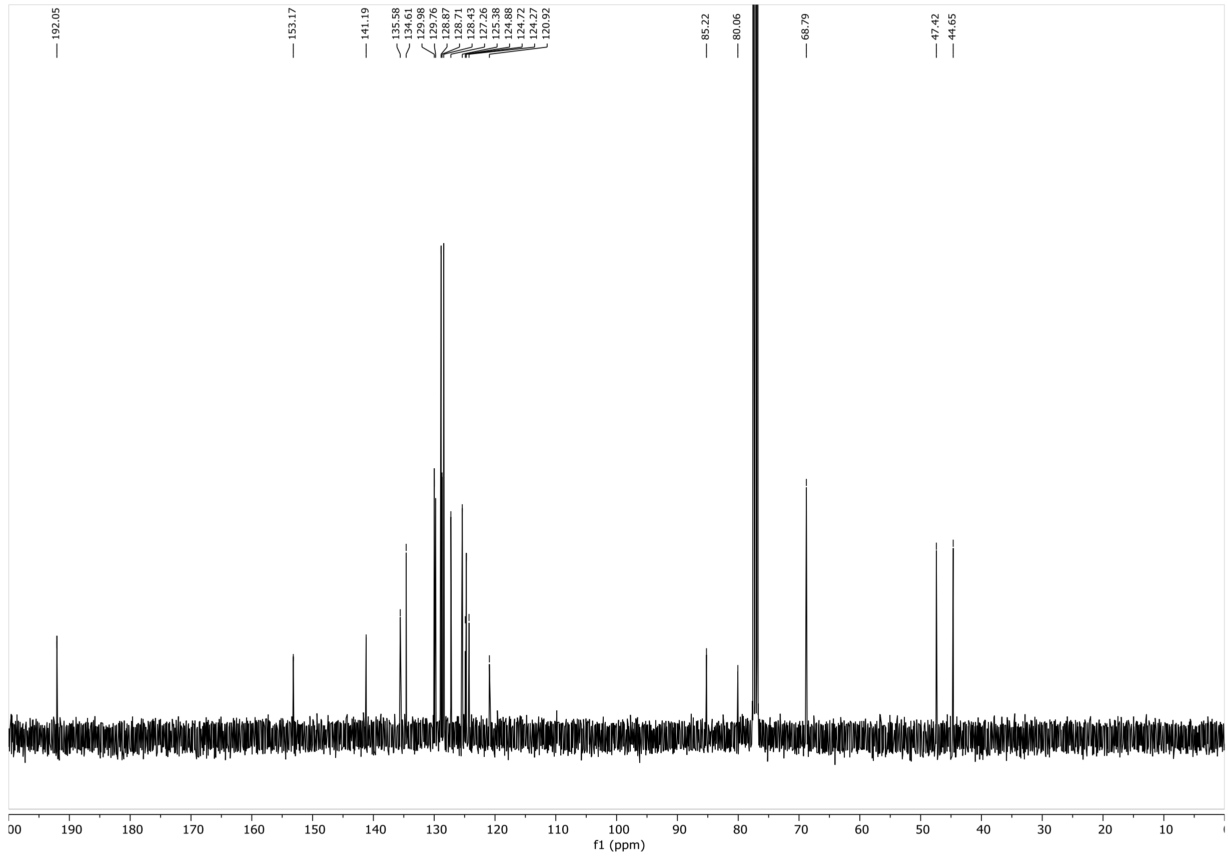

192.05
153.17
141.19
135.58
134.61
129.98
129.76
128.87
128.71
128.43
127.26
125.38
124.88
124.72
124.27
120.92
85.22
80.06
68.79
47.42
44.65
190 180 170 160 150 140 130 120 110 100 90 80 70 60 50 40 30 20 10
f1 (ppm)

Benzyl 2-(cyclopropylethynyl)-4-oxo-3,4-dihydroquinoline-1(2H)-carboxylate (3al) ($CDCl_3$, ^1H 400 MHz, ^{13}C 100 MHz)

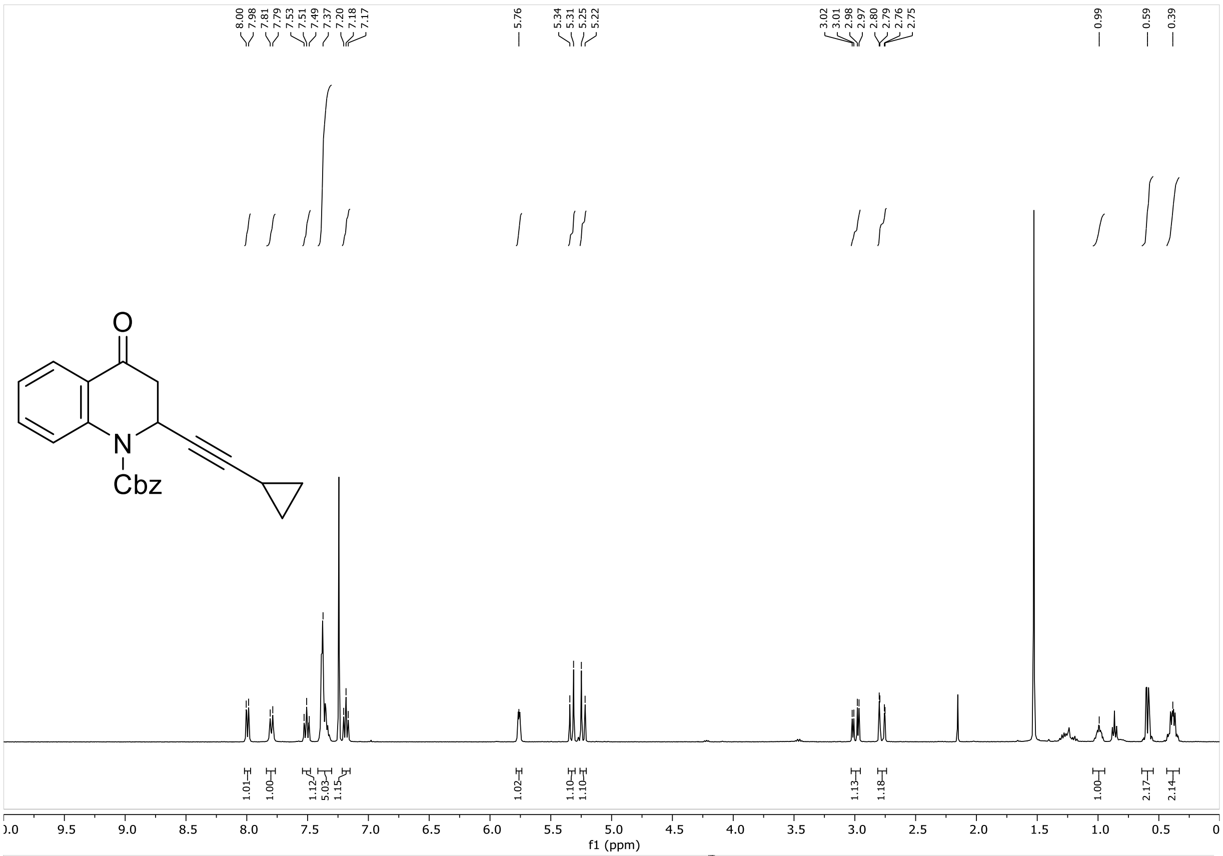

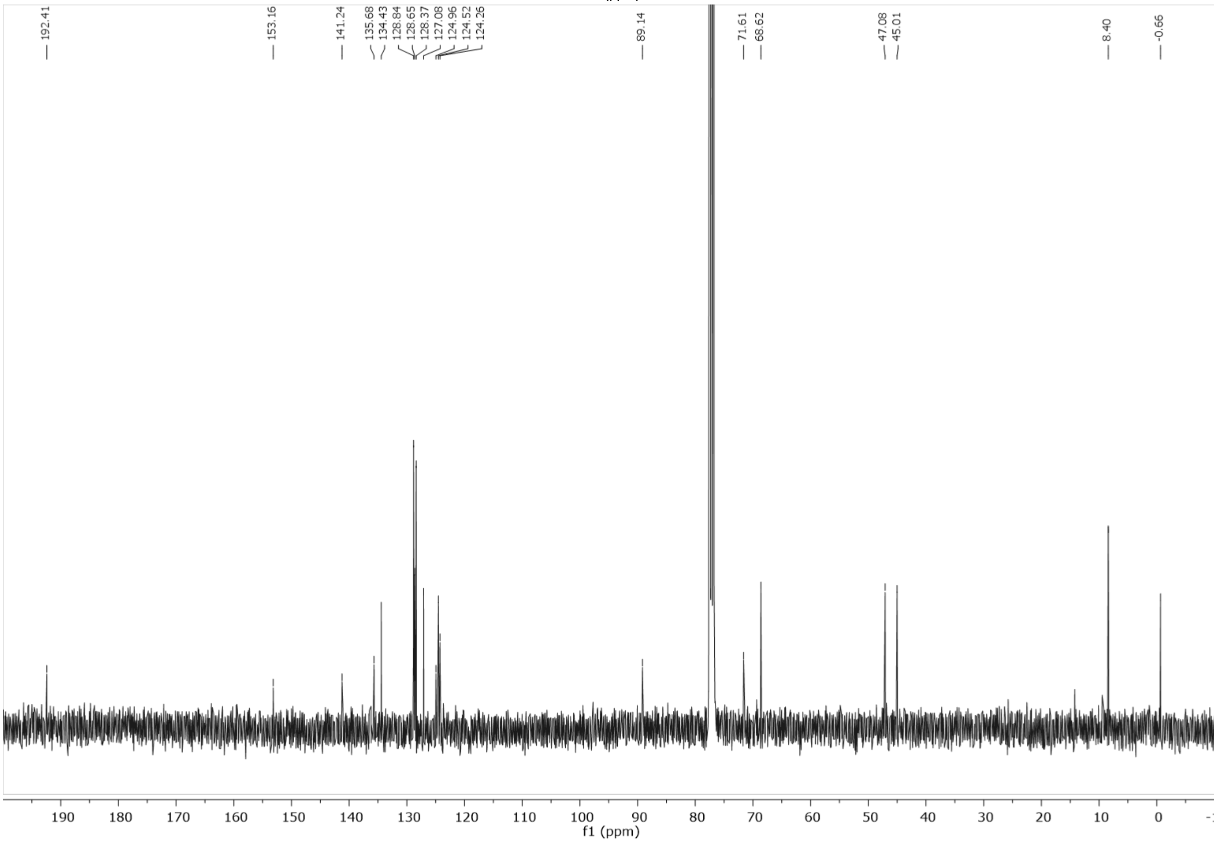

Benzyl 2-(4-methylpent-1-yn-1-yl)-4-oxo-3,4-dihydroquinoline-1(2H)-carboxylate (3am) ($CDCl_3$, ^1H 400 MHz, ^{13}C 100 MHz)

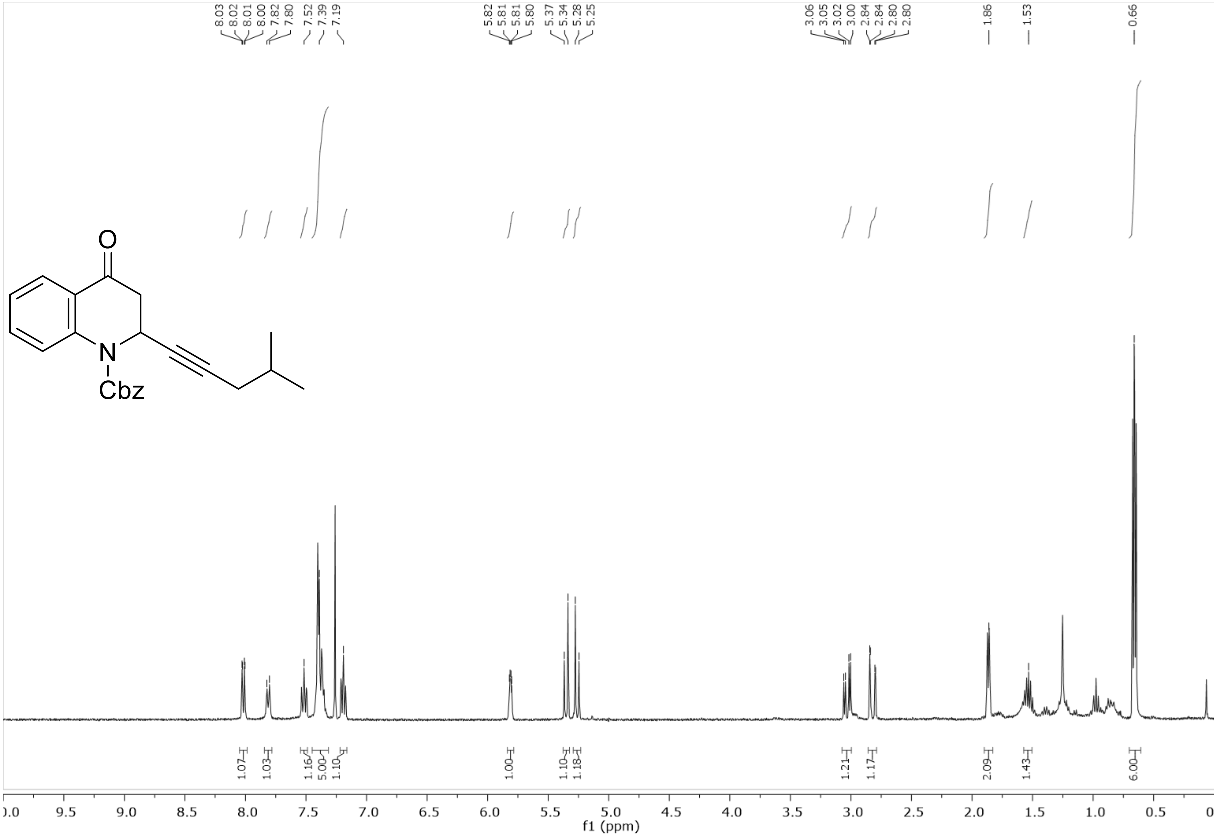

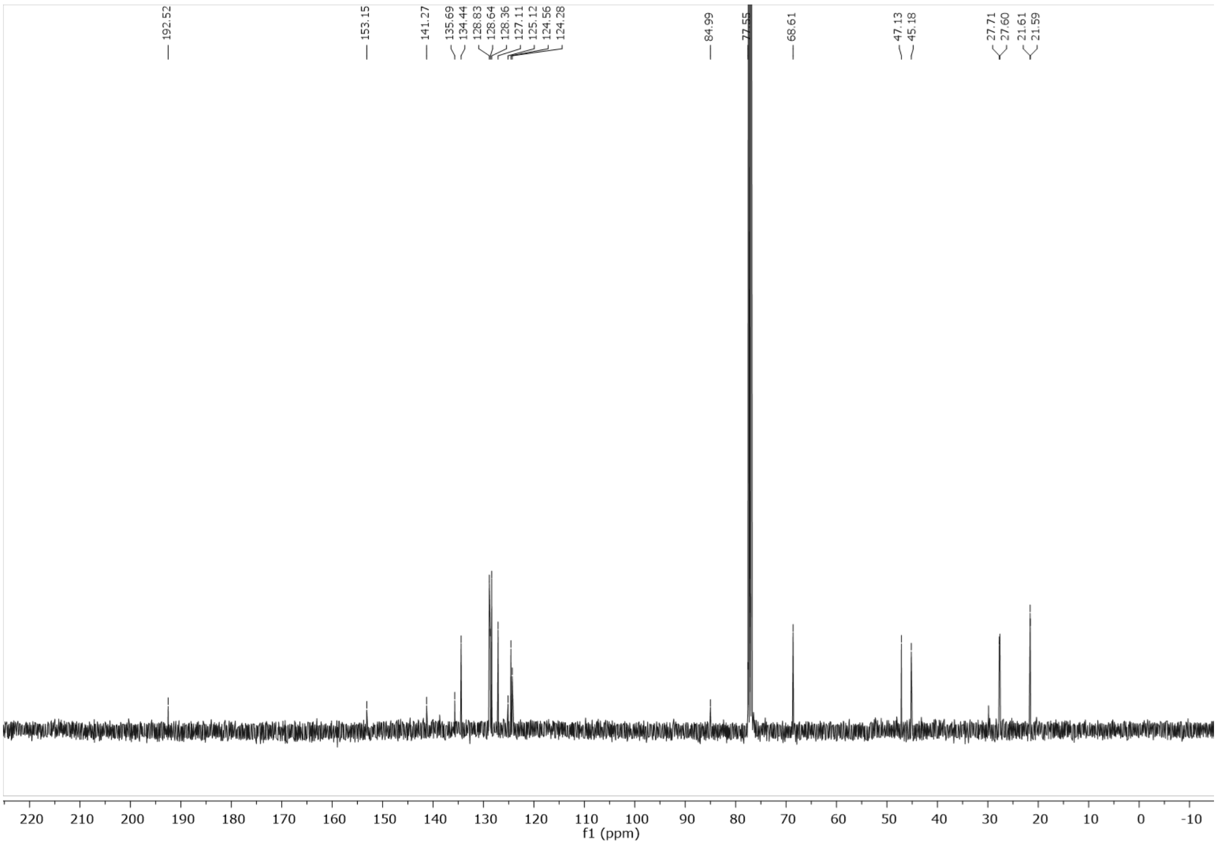

Benzyl 2-(3-methoxy-3-oxoprop-1-yn-1-yl)-4-oxo-3,4-dihydroquinoline-1(2H)-carboxylate (3an) ($CDCl_3$, ^1H 400 MHz, ^{13}C 100 MHz)

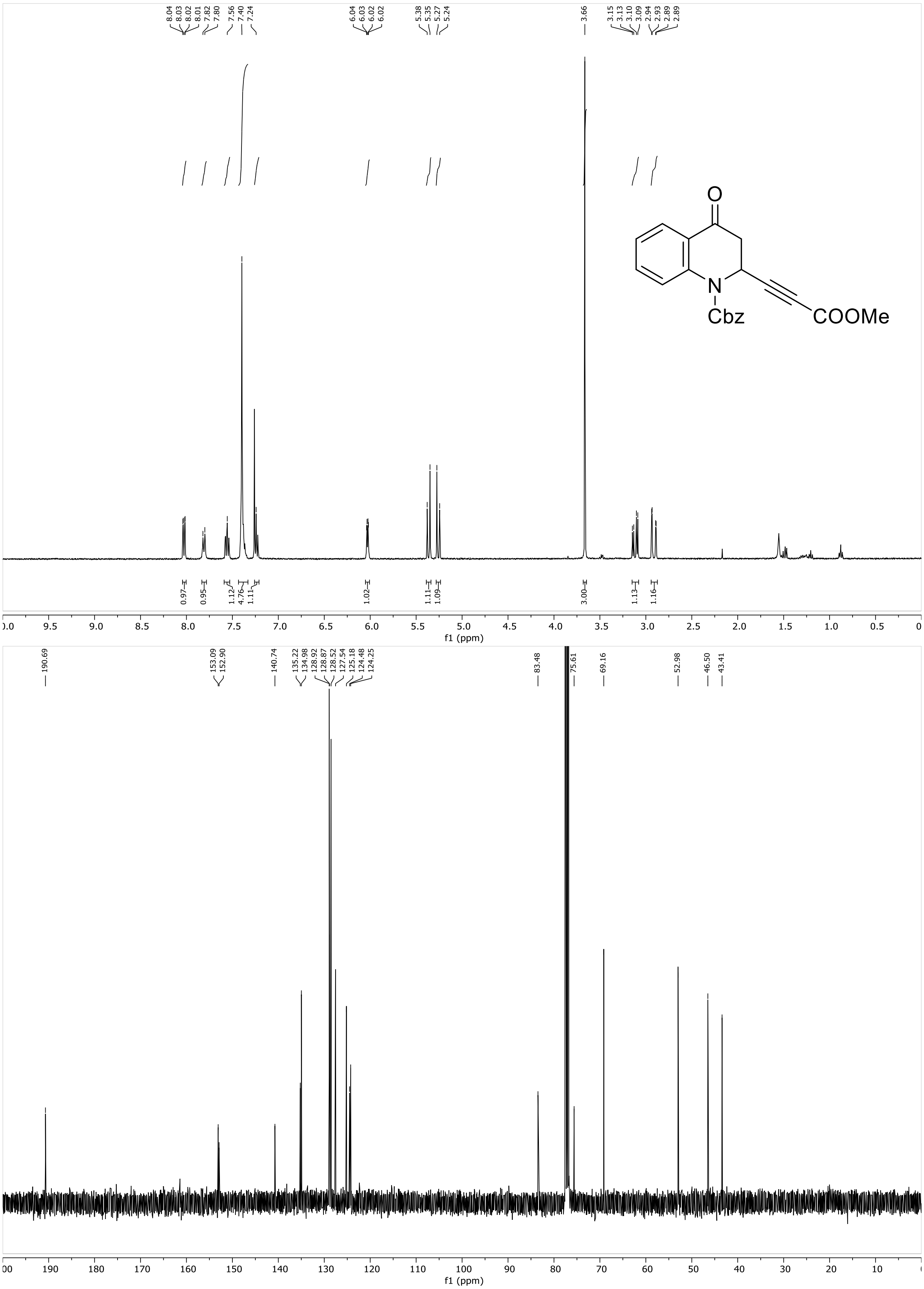

Benzyl 6-methoxy-4-oxo-2-(m-tolylethynyl)-3,4-dihydroquinoline-1(2H)-carboxylate (3eg) ($CDCl_3$, ^1H 400 MHz, ^{13}C 100 MHz)

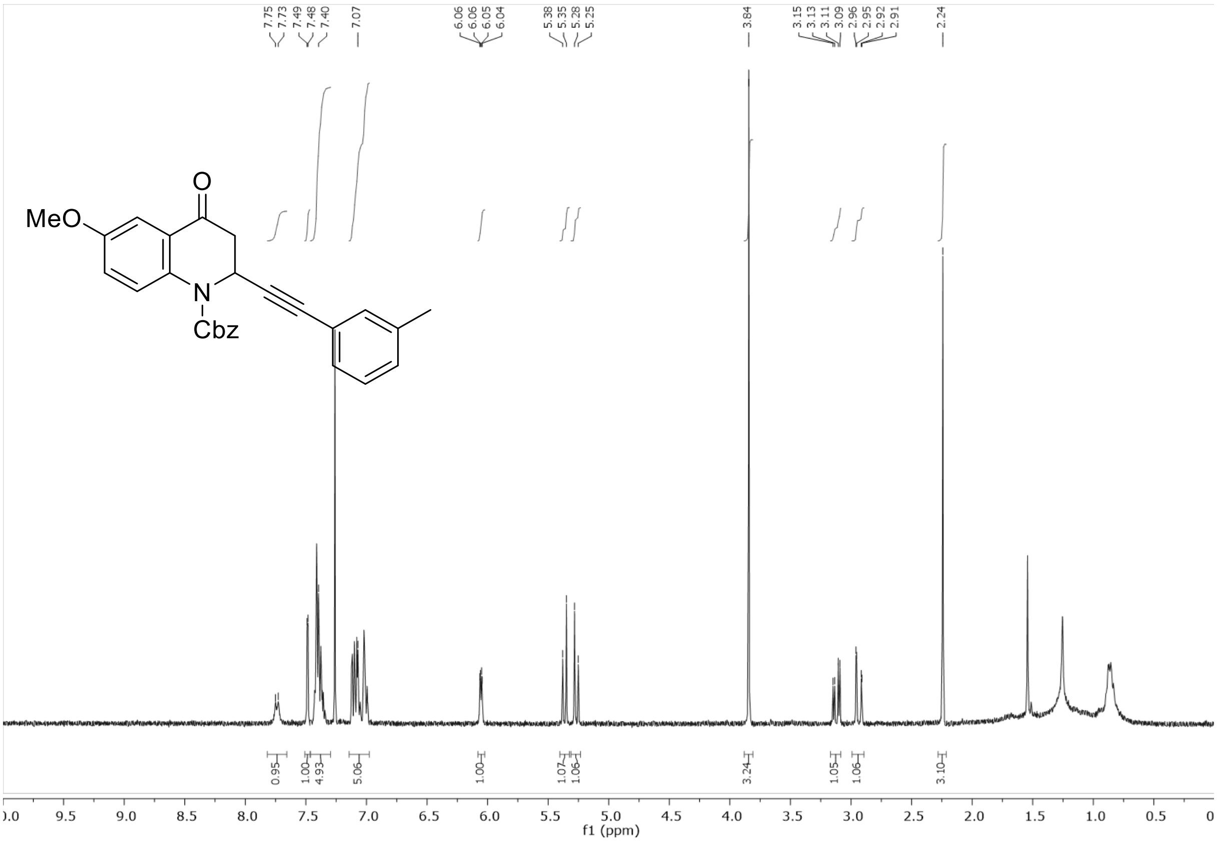

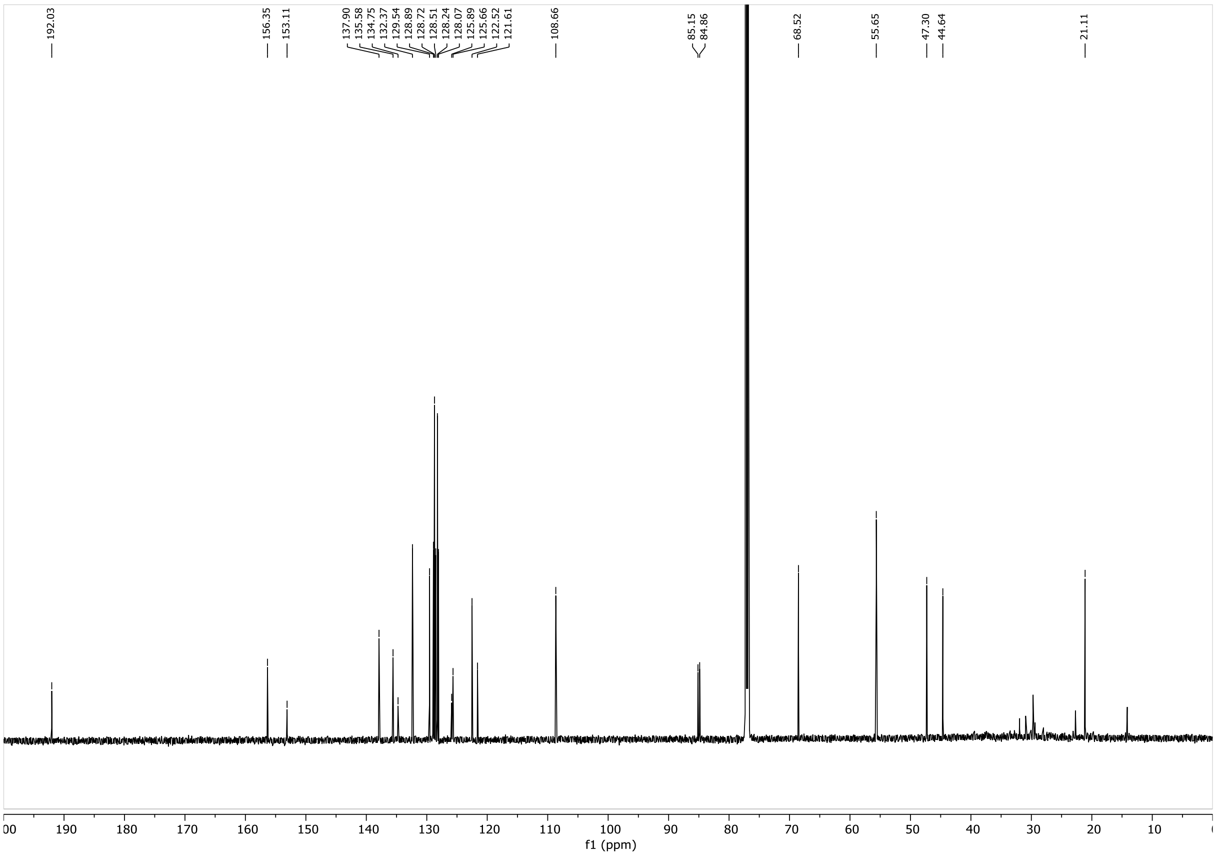

Benzyl 4-oxo-2-(m-tolylethynyl)-6-(trifluoromethyl)-3,4-dihydroquinoline-1(2H)-carboxylate (3fg) ($CDCl_3$, ^1H 400 MHz, ^{13}C 100 MHz, ^{19}F 376 MHz)

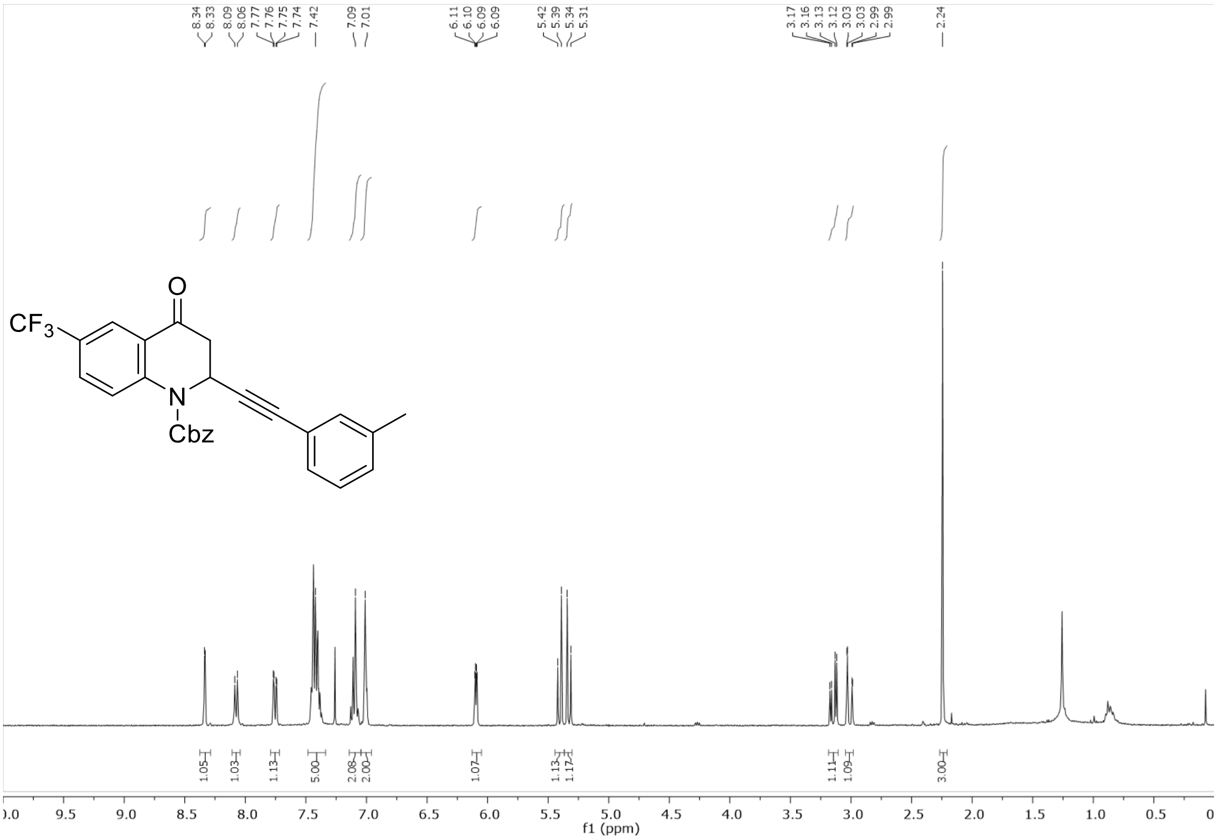

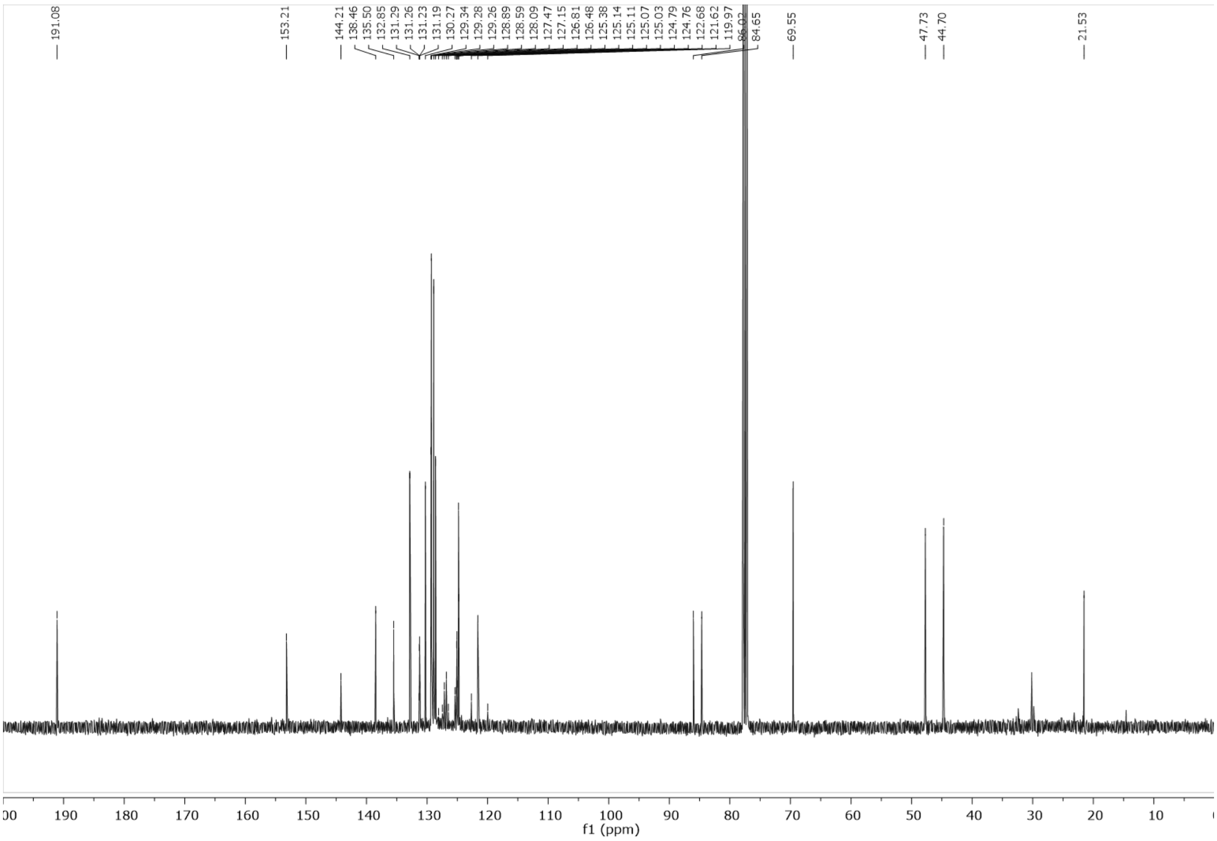

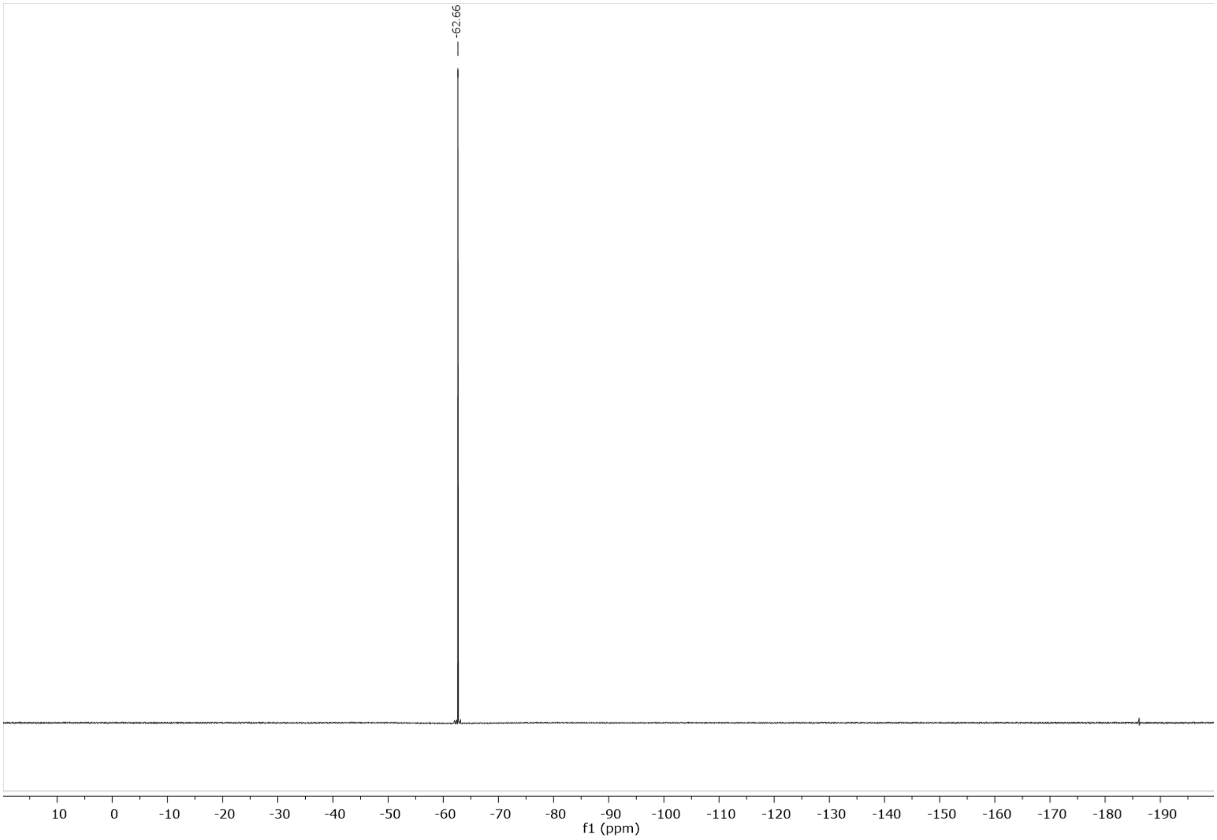

-62.66
10
0
-10
-20
-30
-40
-50
-60
-70
-80
-90
-100
-110
-120
-130
-140
-150
-160
-170
-180
-190
f1 (ppm)

Benzyl 6-bromo-4-oxo-2-(m-tolylethynyl)-3,4-dihydroquinoline-1(2H)-carboxylate (3gg) ($CDCl_3$, ^1H 400 MHz, ^{13}C 100 MHz)

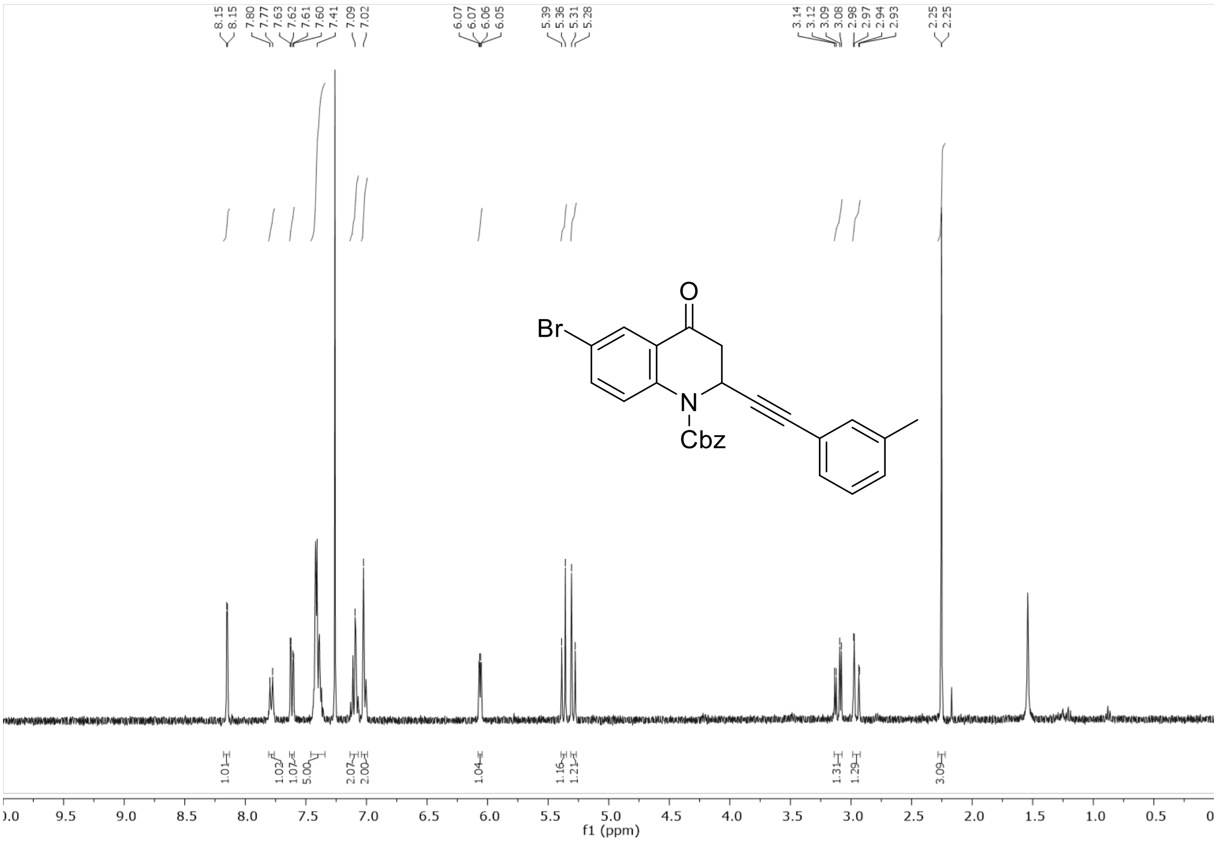

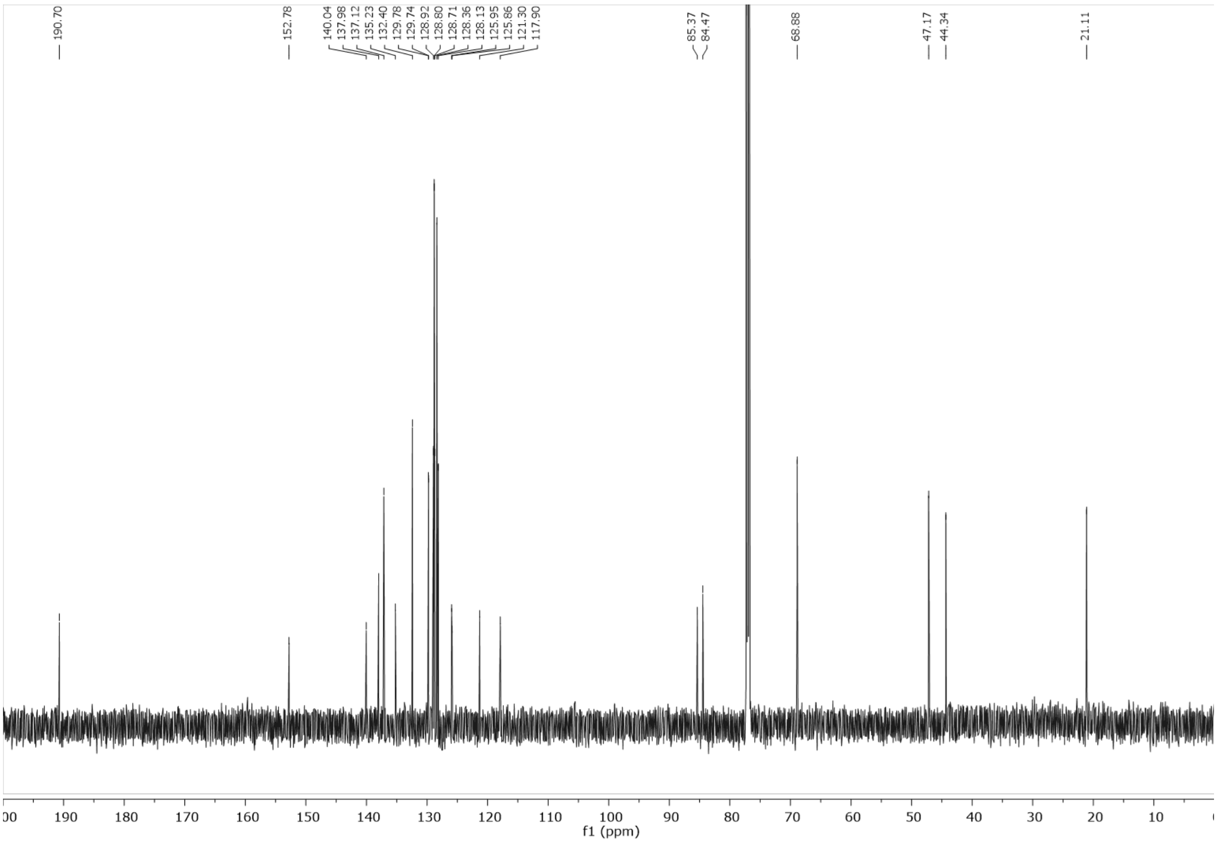

Benzyl 6-methyl-4-oxo-2-(m-tolylethynyl)-3,4-dihydroquinoline-1(2H)-carboxylate (3hg) ($CDCl_3$, ^{1}H 400 MHz, ^{13}C 100 MHz)

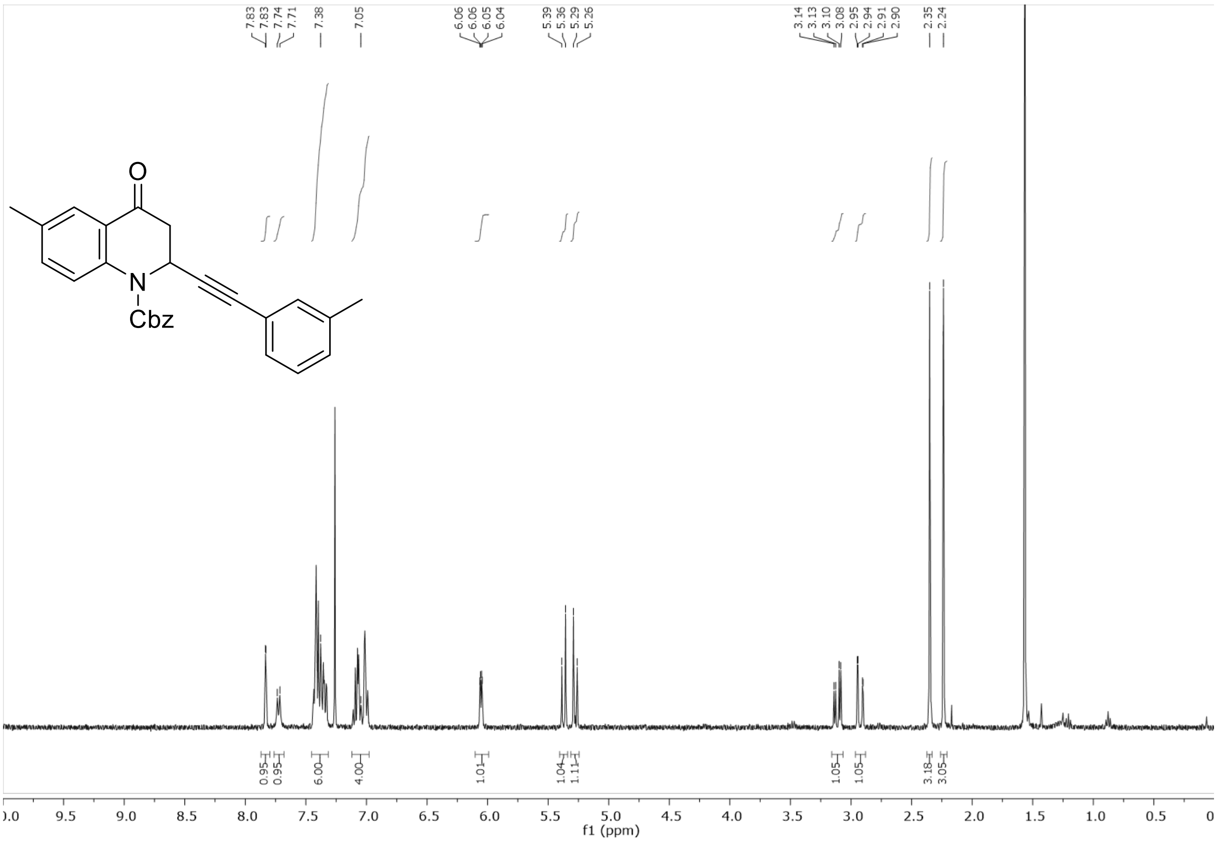

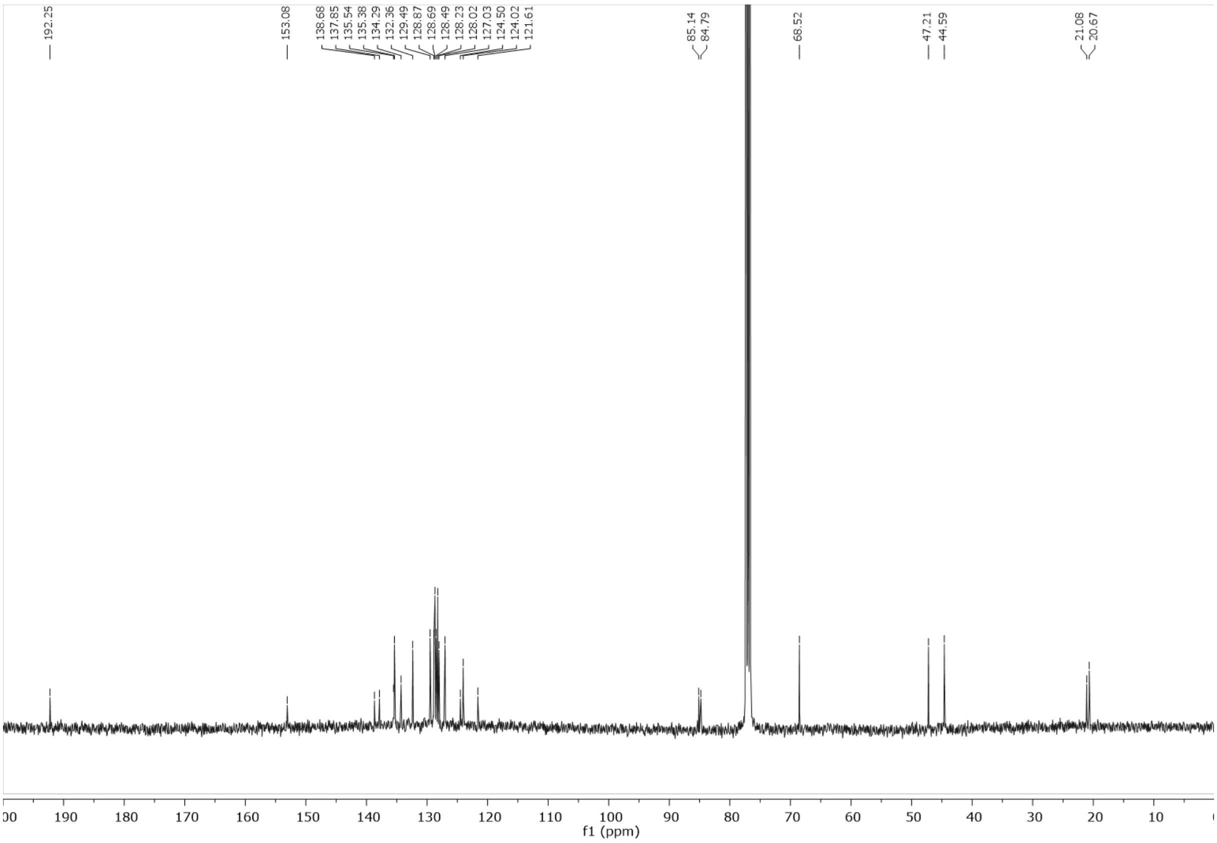

1-Benzyl 6-methyl 4-oxo-2-(m-tolylethynyl)-3,4-dihydroquinoline-1,6(2H)-dicarboxylate (3ig) ($CDCl_3$, ^1H 400 MHz, ^{13}C 100 MHz)

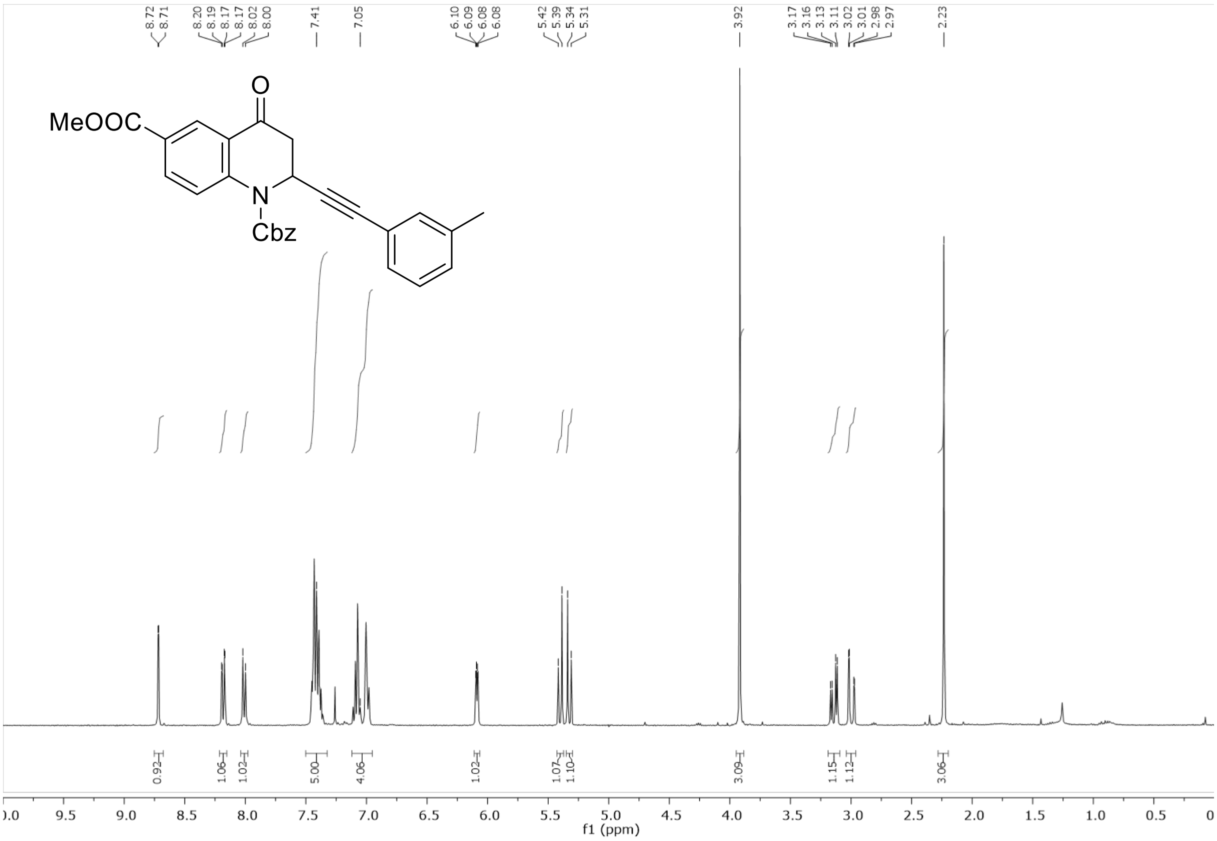

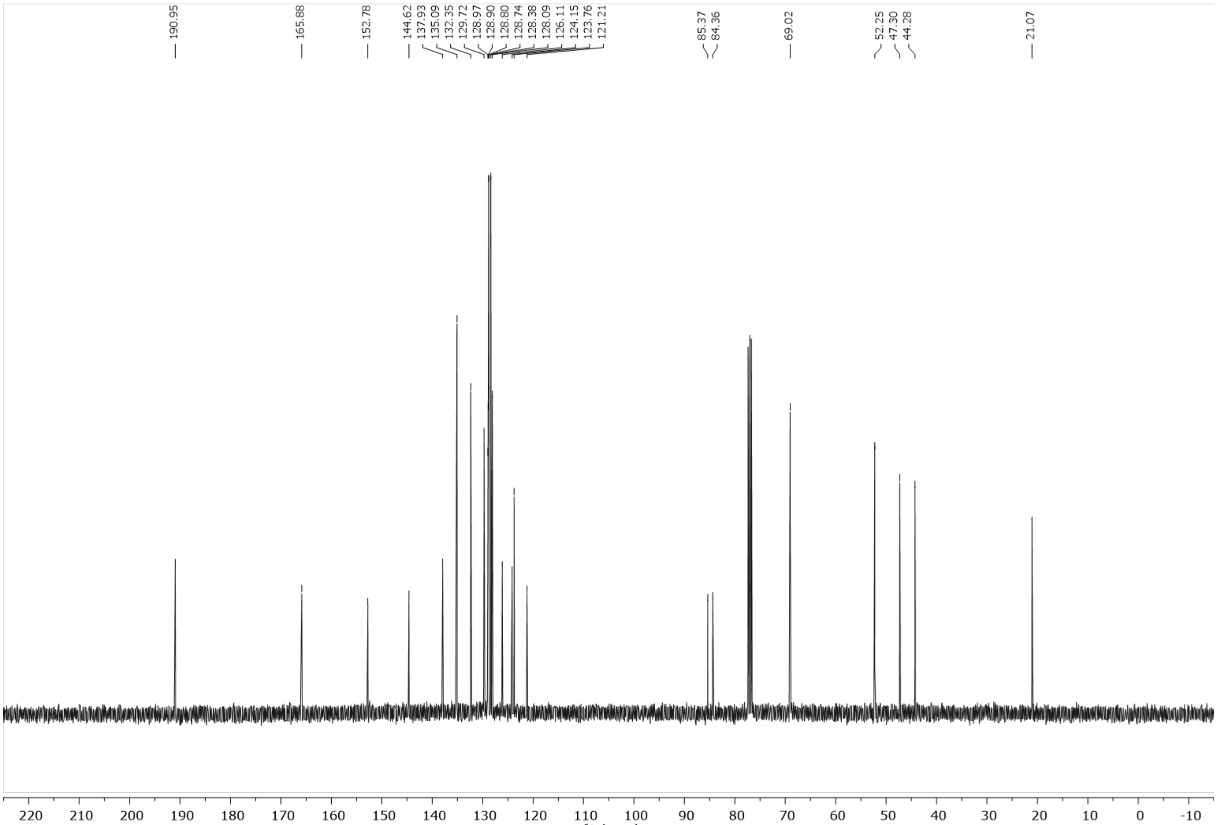

Benzyl 8-oxo-6-(m-tolylethynyl)-7,8-dihydro-[1,3]dioxolo[4,5-g]quinoline-5(6H)-carboxylate (3jg)
($CDCl_3$, ^1H 400 MHz, ^{13}C 100 MHz)

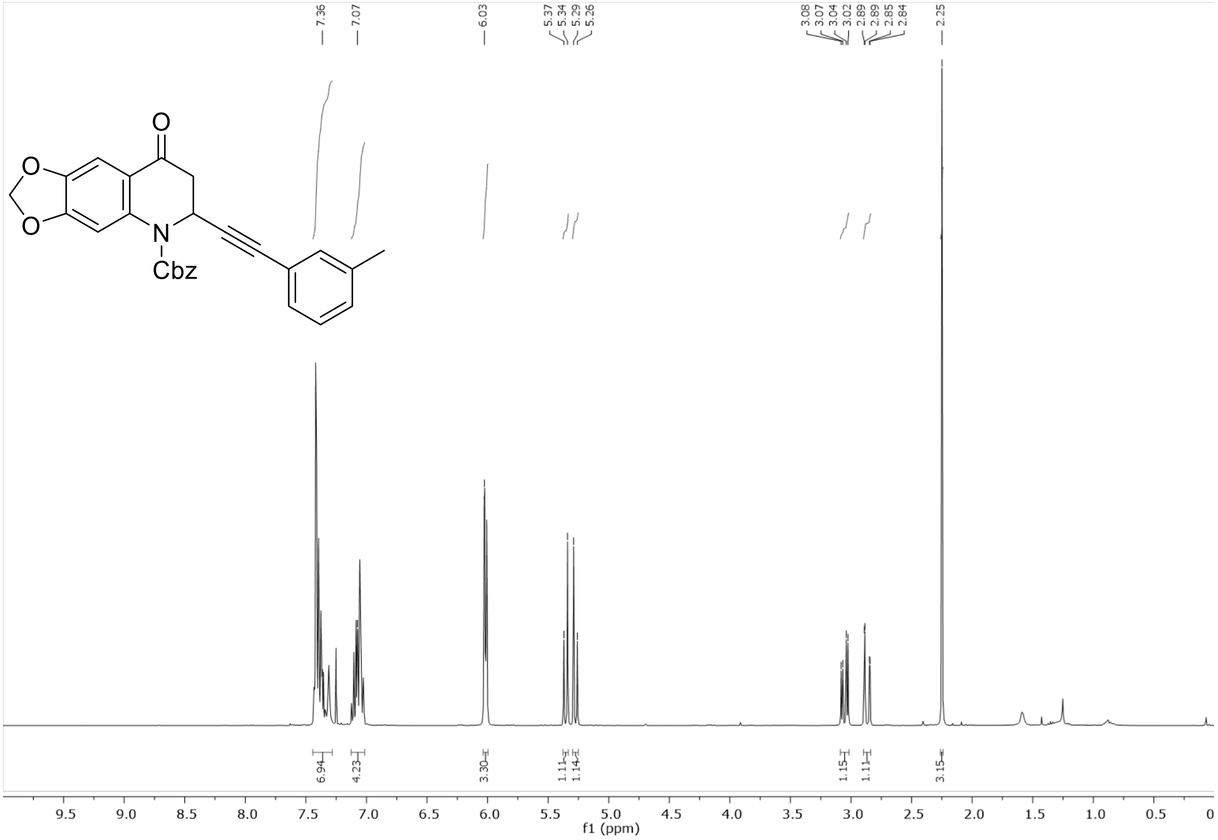

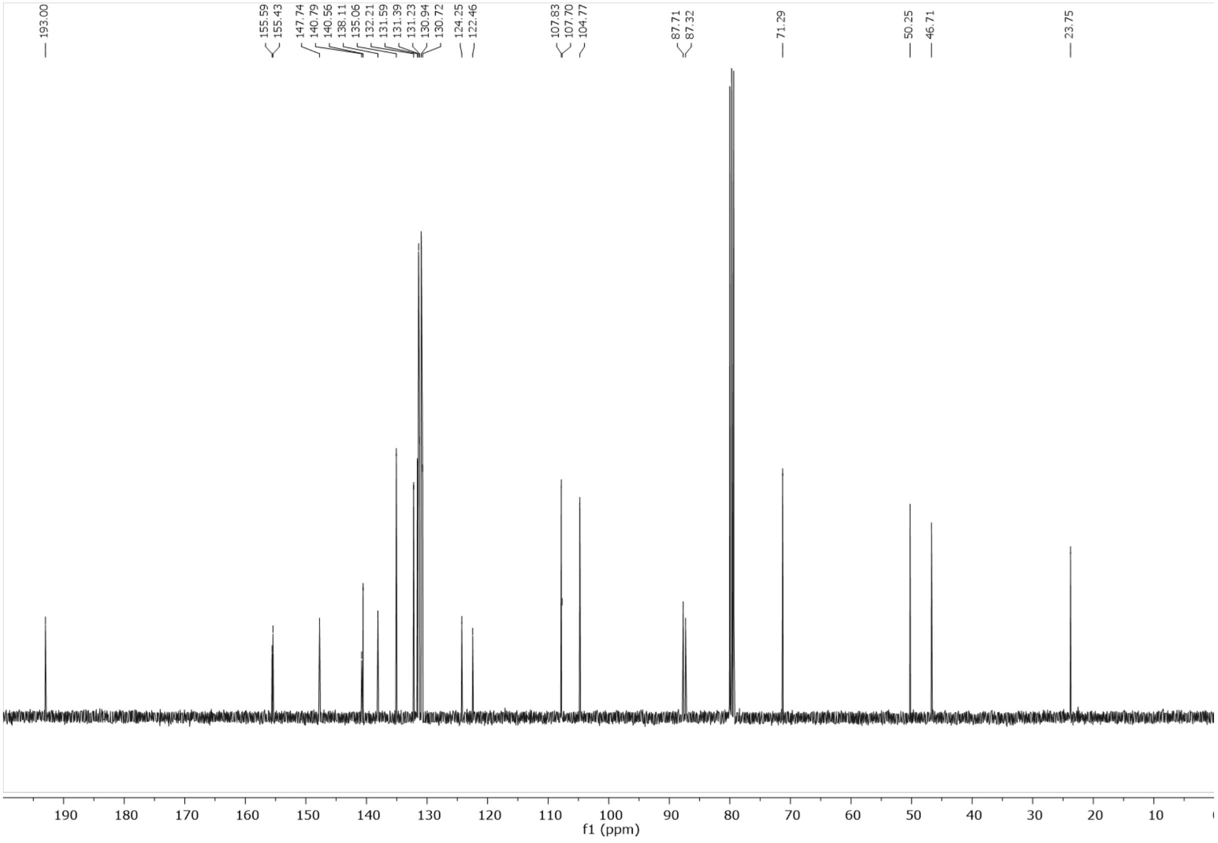

Benzyl 5,7-dimethyl-4-oxo-2-(m-tolylethynyl)-3,4-dihydroquinoline-1(2H)-carboxylate (3kg) ($CDCl_3$, ^1H 400 MHz, ^{13}C 100 MHz)

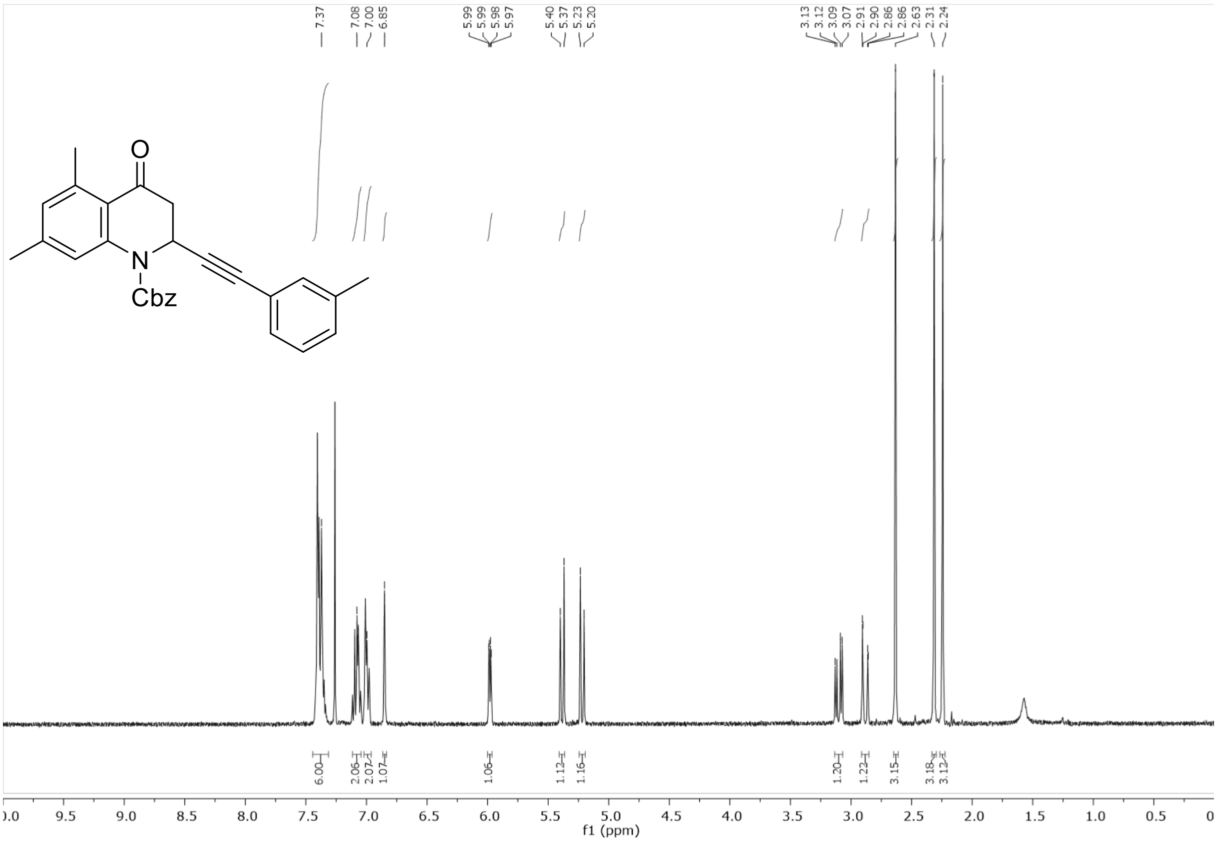

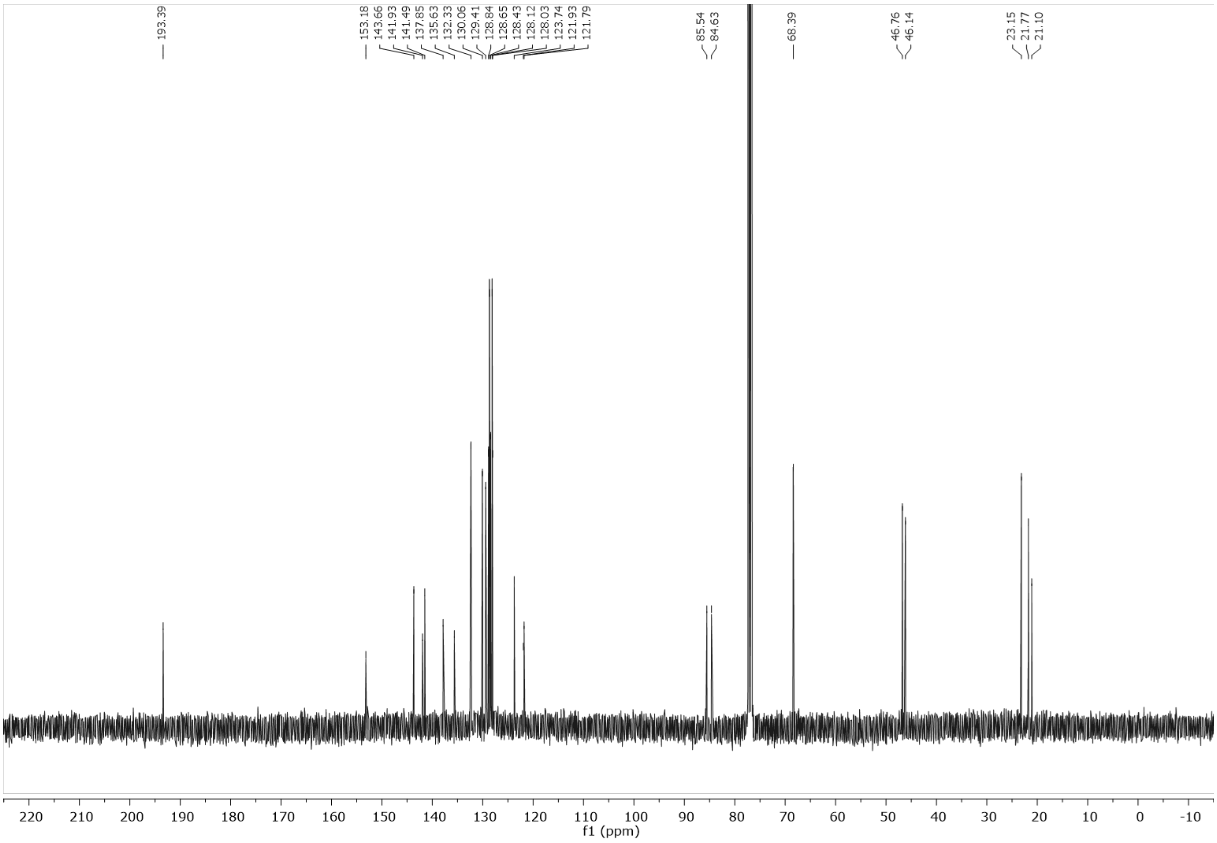

(*R*)–Benzyl 2-((3,4-dimethoxyphenyl)ethynyl)-4-oxo-3,4-dihydroquinoline-1(2H)-carboxylate (3ap) ($CDCl_3$, ^1H 400 MHz, ^{13}C 100 MHz)

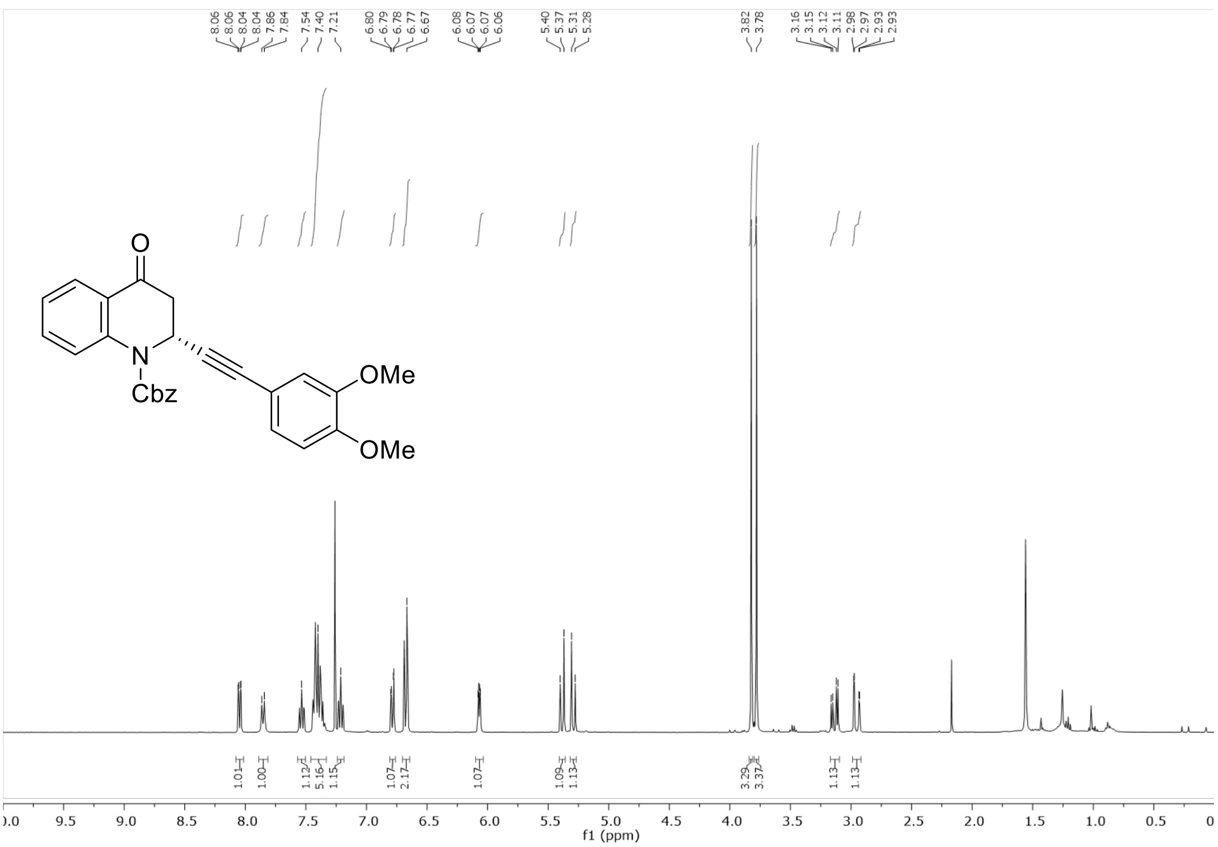

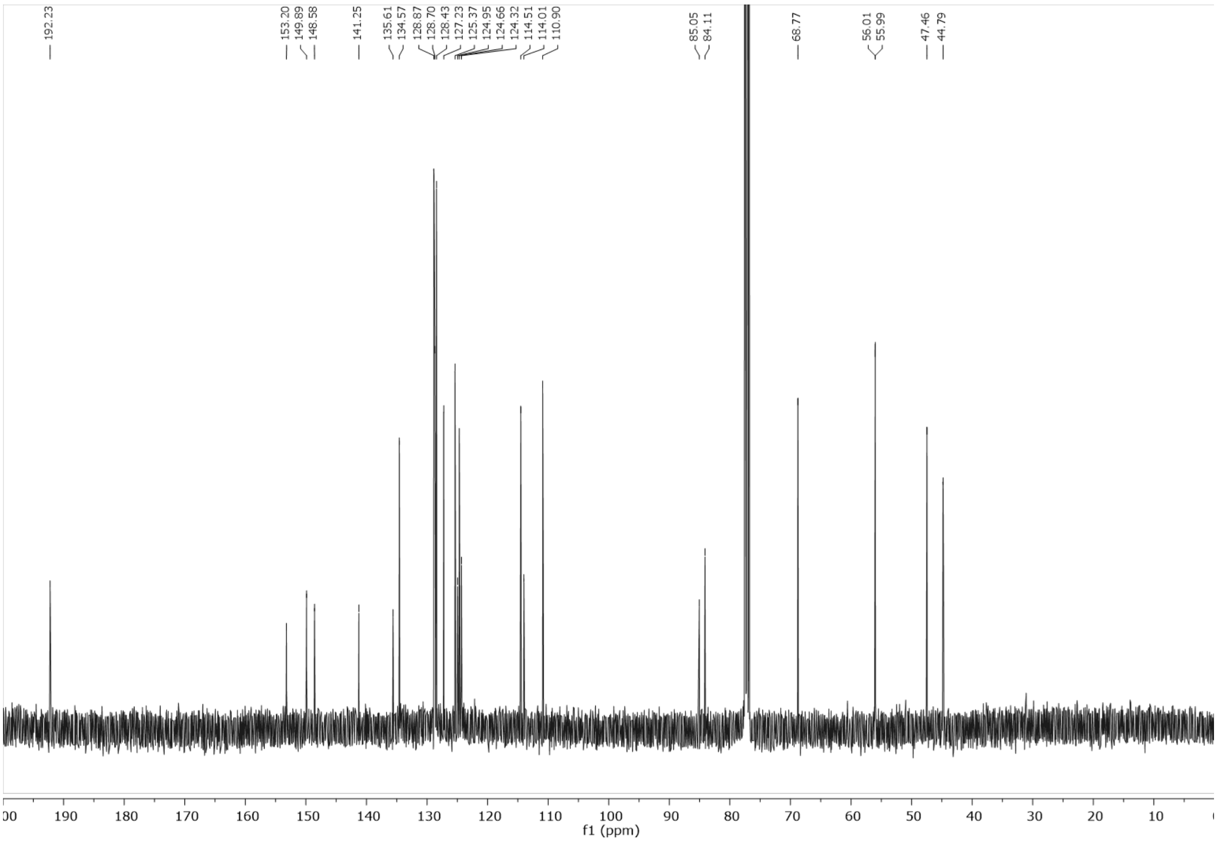

NMR spectra of compounds **4** and **5**

2-(phenylethynyl)-2,3-dihydroquinolin-4(1H)-one (4) ($CDCl_3$, ^1H 400 MHz, ^{13}C 100 MHz)

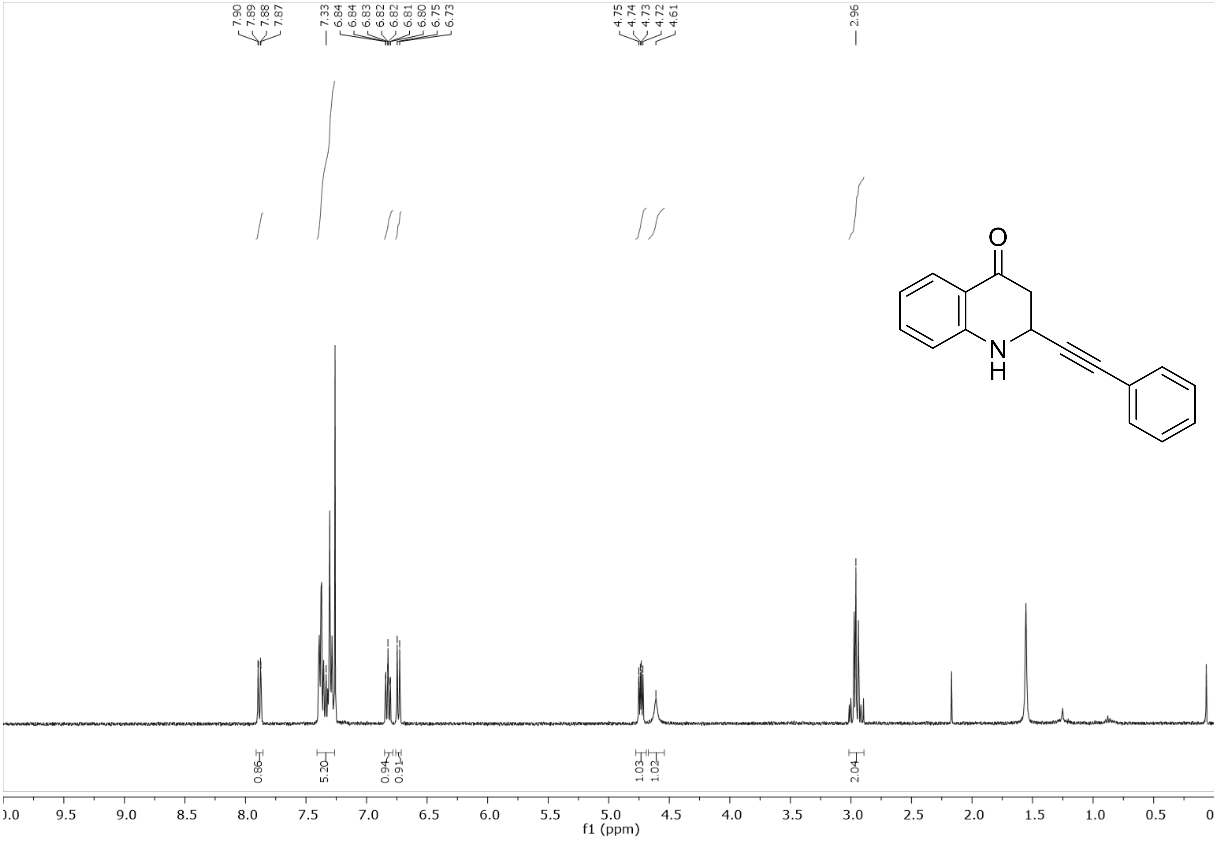

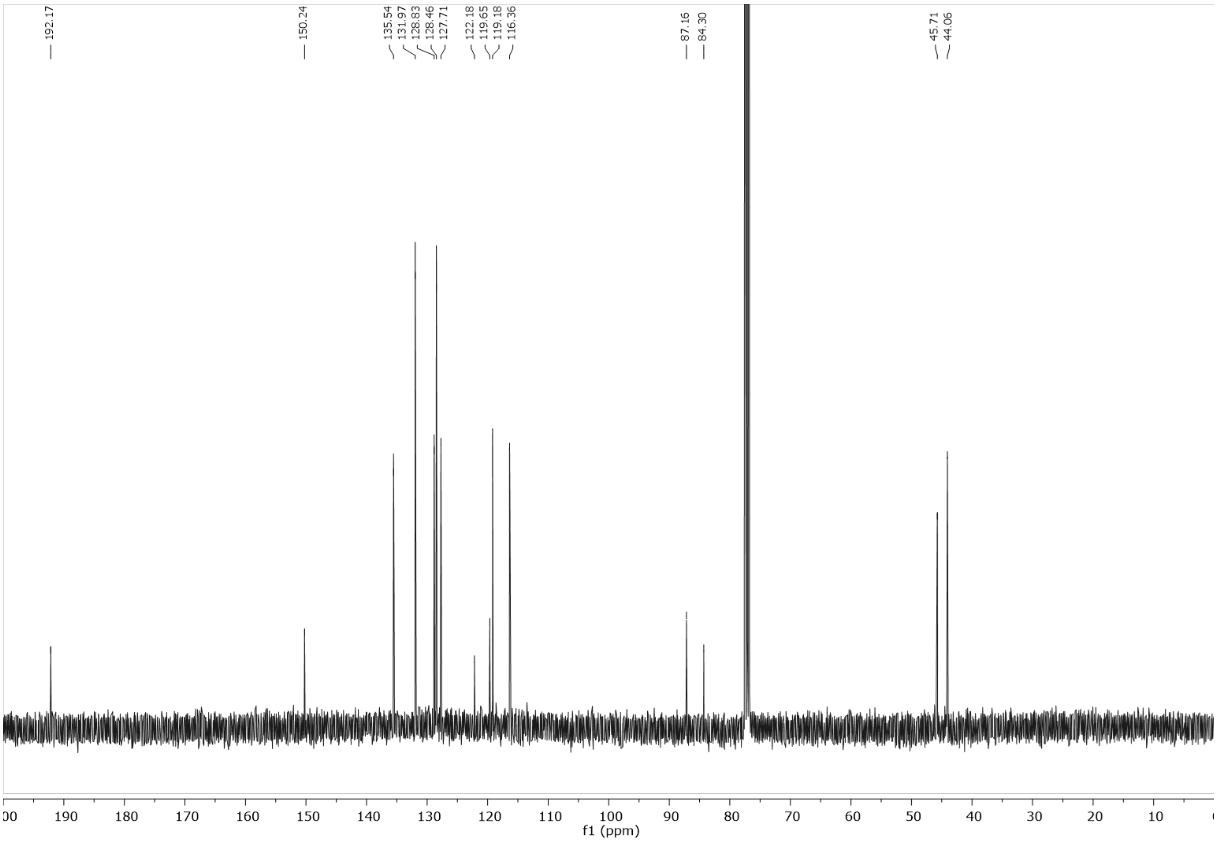

(+)-Cuspareine (5) ($CDCl_3$, ^{1}H 400 MHz, ^{13}C 100 MHz)

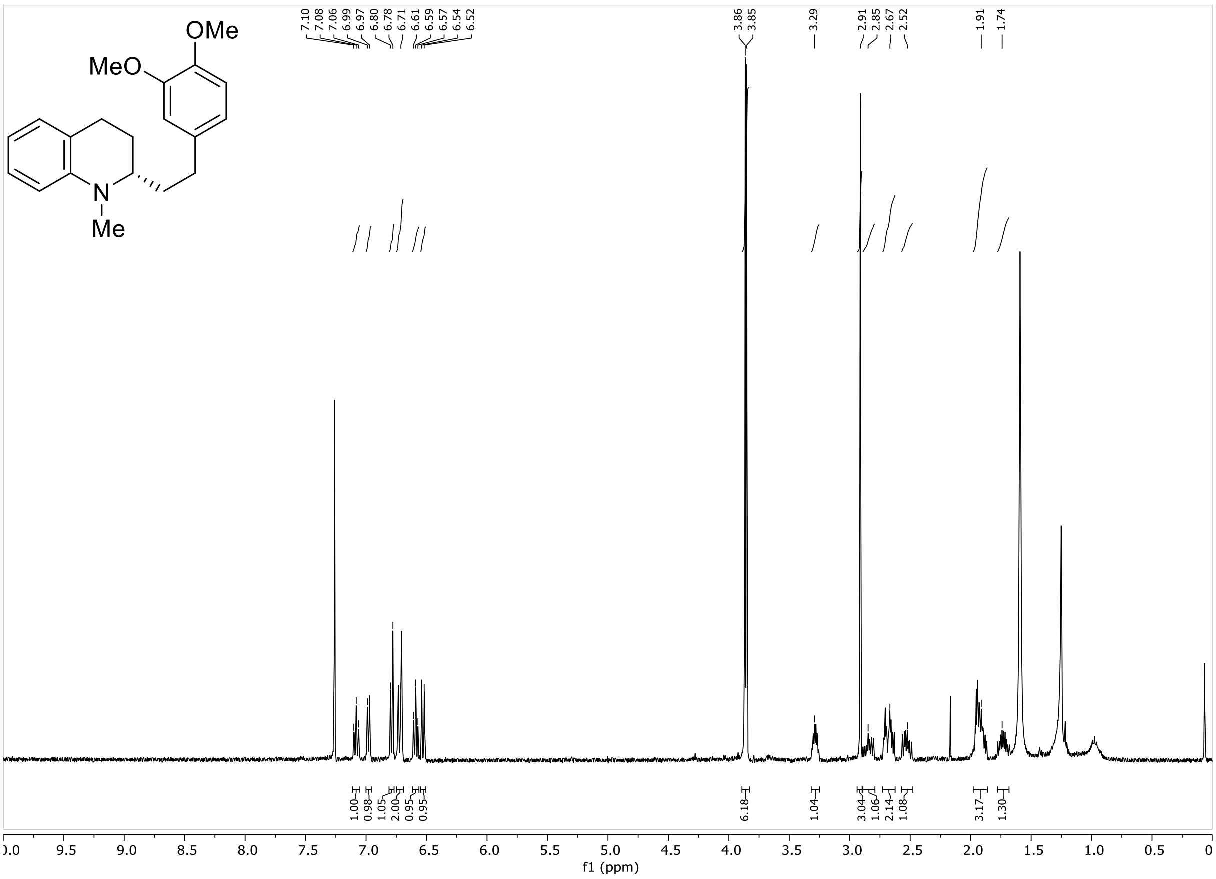

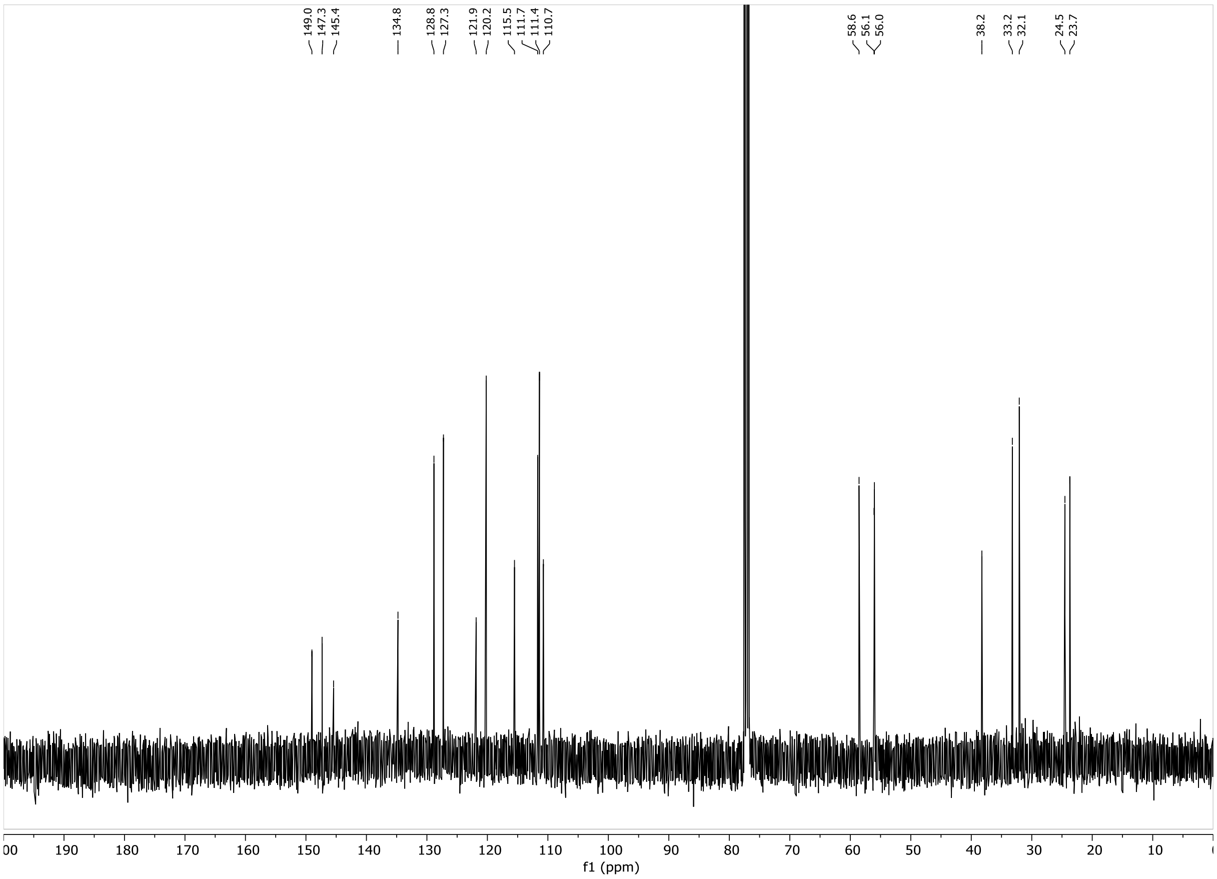
149.0
147.3
145.4
134.8
128.8
127.3
121.9
120.2
115.5
111.7
111.4
110.7
58.6
56.1
56.0
38.2
33.2
32.1
24.5
23.7
f1 (ppm)
